# Multi-modality artificial intelligence-based transthyretin amyloid cardiomyopathy detection in patients with severe aortic stenosis

**DOI:** 10.1007/s00259-024-06922-4

**Published:** 2024-09-23

**Authors:** Isaac Shiri, Sebastian Balzer, Giovanni Baj, Benedikt Bernhard, Moritz Hundertmark, Adam Bakula, Masaaki Nakase, Daijiro Tomii, Giulia Barbati, Stephan Dobner, Waldo Valenzuela, Axel Rominger, Federico Caobelli, George C. M. Siontis, Jonas Lanz, Thomas Pilgrim, Stephan Windecker, Stefan Stortecky, Christoph Gräni

**Affiliations:** 1 Department of Cardiology, Inselspital Bern University Hospital, University of Bern, Freiburgstrasse, Bern, CH – 3010 Switzerland; 2 Biostatistics Unit, Department of Medical Sciences, University of Trieste, Trieste, Italy; 3 University Institute for Diagnostic and Interventional Neuroradiology, Inselspital, Bern University Hospital, University of Bern, Freiburgstrasse, Bern, 3010 Switzerland; 4 Department of Nuclear Medicine, Inselspital Bern University Hospital, University of Bern, Bern, Switzerland

**Keywords:** Transthyretin amyloid cardiomyopathy, Aortic stenosis, TAVI, Artificial intelligence, Radiomics

## Abstract

**Purpose:**

Transthyretin amyloid cardiomyopathy (ATTR-CM) is a frequent concomitant condition in patients with severe aortic stenosis (AS), yet it often remains undetected. This study aims to comprehensively evaluate artificial intelligence-based models developed based on preprocedural and routinely collected data to detect ATTR-CM in patients with severe AS planned for transcatheter aortic valve implantation (TAVI).

**Methods:**

In this prospective, single-center study, consecutive patients with AS were screened with [^99m^Tc]-3,3-diphosphono-1,2-propanodicarboxylic acid ([^99m^Tc]-DPD) for the presence of ATTR-CM. Clinical, laboratory, electrocardiogram, echocardiography, invasive measurements, 4-dimensional cardiac CT (4D-CCT) strain data, and CT-radiomic features were used for machine learning modeling of ATTR-CM detection and for outcome prediction. Feature selection and classifier algorithms were applied in single- and multi-modality classification scenarios. We split the dataset into training (70%) and testing (30%) samples. Performance was assessed using various metrics across 100 random seeds.

**Results:**

Out of 263 patients with severe AS (57% males, age 83 ± 4.6years) enrolled, ATTR-CM was confirmed in 27 (10.3%). The lowest performances for detection of concomitant ATTR-CM were observed in invasive measurements and ECG data with area under the curve (AUC) < 0.68. Individual clinical, laboratory, interventional imaging, and CT-radiomics-based features showed moderate performances (AUC 0.70–0.76, sensitivity 0.79–0.82, specificity 0.63–0.72), echocardiography demonstrated good performance (AUC 0.79, sensitivity 0.80, specificity 0.78), and 4D-CT-strain showed the highest performance (AUC 0.85, sensitivity 0.90, specificity 0.74). The multi-modality model (AUC 0.84, sensitivity 0.87, specificity 0.76) did not outperform the model performance based on 4D-CT-strain only data (p-value > 0.05). The multi-modality model adequately discriminated low and high-risk individuals for all-cause mortality at a mean follow-up of 13 months.

**Conclusion:**

Artificial intelligence-based models using collected pre-TAVI evaluation data can effectively detect ATTR-CM in patients with severe AS, offering an alternative diagnostic strategy to scintigraphy and myocardial biopsy.

**Supplementary Information:**

The online version contains supplementary material available at 10.1007/s00259-024-06922-4.

## Introduction

Transthyretin amyloid cardiomyopathy (ATTR-CM) is an underdiagnosed progressive cardiac disorder caused by the misfolding of transthyretin amyloid protein, leading to the deposition of amyloid fibrils in the extracellular space of cardiac tissues [1]. The amyloid accumulation can result in abnormalities in atrioventricular conduction and stiffening of the myocardial tissue, which ultimately impairs cardiac function, leading to heart failure and impaired prognosis [1, 2]. In addition to the myocardium, amyloid fibrils can affect valve tissue, damaging endothelial cells and eventually causing calcification, particularly of the aortic valves, facilitating the development of aortic stenosis (AS) [1–3]. Recent studies have suggested that the coexistence of severe AS and ATTR-CM is more frequent than previously anticipated and associated with an increased risk of adverse events after transcatheter aortic valve implantation (TAVI) [4–7]. Although TAVI has been shown effective in this high-risk patient population, it is unlikely to achieve sustained improvement in symptoms and prognosis without addressing the underlying cardiomyopathy, for which reason timely diagnosis and subsequent treatment of ATTR-CM are central to the optimal patient management in this specific population [7].

Current ESC [8] and ACC/AHA [9, 10] guidelines propose different algorithms for ATTR-CM diagnosis [1]. Initial assessments typically involve clinical examinations, electrocardiogram (ECG), echocardiography (Echo), and cardiovascular magnetic resonance imaging (CMR) to include or exclude potential patients based on specific symptoms [1]. For example, bilateral carpal tunnel syndrome and peripheral neuropathy as clinical features, low QRS voltage and pseudo-infarct patterns in ECG, apical sparing, or increased atrial/RV wall thickness in echocardiography could serve as red flags for ATTR-CM [1, 11–14]. While these modalities are useful in the preliminary evaluation, they are not specific to ATTR-CM; thus, a final diagnosis often cannot be based entirely on these results [1, 15, 16].

A definitive diagnosis of ATTR-CM may hinge on the histopathological confirmation or proof of a TTR mutation whilst always requiring confirmation of cardiac involvement, e.g., by significant cardiac uptake in scintigraphy [1, 17, 18]. Pathology and genetic testing are invasive and costly, while scintigraphy adds a significant financial and procedural burden, especially for severe AS patients undergoing TAVI who have already undergone extensive examinations [1–3]. Therefore, developing a non-invasive, financially viable method based on available data from preprocedural and routine data would be highly beneficial for detecting ATTR-CM.

Different studies have applied artificial intelligence (AI) to detect and screen for ATTR-CM across different data modalities [1, 19, 20]. AI-driven algorithms employing both deep learning (DL) and machine learning (ML) across clinical, echocardiography, ECG, scintigraphy, and CMR imaging have shown enhanced diagnostic accuracy [1]. Our study’s main aim is to develop and comprehensively evaluate ML models using a pre-procedural and routinely collected TAVI multimodality dataset for detecting ATTR-CM. By evaluating ML algorithms within and across modalities in the same patient cohort, we offer insights into the strengths and limitations of each approach for ML-based ATTR-CM detection using different modalities.

## Materials and methods

**Fig. 1:**
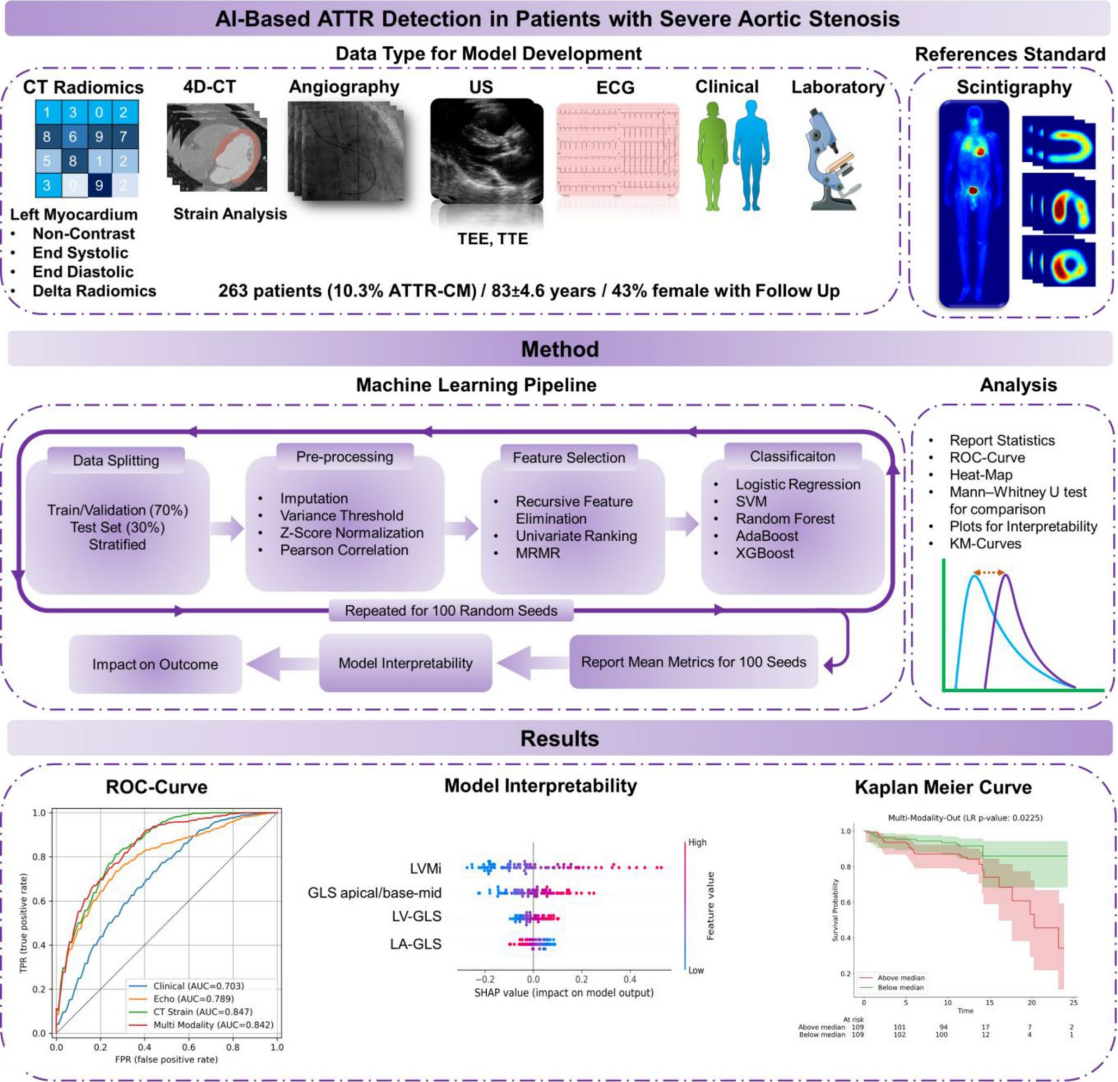
The flowchart of the current study represents the design of the study, starting the phases of data collection, preprocessing, model training, validation, and reporting phases utilized in the current study

Figure 1 presents the study overview, including data collection, preprocessing, model training, validation, testing, and reporting phases utilized in the current study. The study follows the Transparent Reporting of a multivariable prediction model for Individual Prognosis Or Diagnosis + Artificial Intelligence (TRIPOD + AI) statement [21].

### Study design and population

The data for this study were collected from multiple modalities to enable a comprehensive analysis of ATTR-CM detection in patients with severe AS planned for TAVI [22, 23]. Consecutive patients (between August 2019 and 2021) with symptomatic severe AS in the absence of known cardiac or extra-cardiac amyloidosis were referred for TAVI at Bern University Hospital and recruited in the ATTR-AS (Amyloid Transthyretin in Aortic Stenosis, NCT04061213) study (ClinicalTrials.gov: NCT04061213) were considered eligible [22, 23]. The study design was approved by the Bern ethics committee, conducted in accordance with the Declaration of Helsinki, and study participants provided written informed consent before any data collection [22, 23]. Baseline and follow-up clinical data were prospectively recorded in a dedicated database held at the clinical trials unit of Bern University Hospital [22, 23]. These included clinical assessments, laboratory tests, ECG, and echocardiography (transthoracic echocardiography (TTE)). Additionally, left and right heart catheterization and various forms of interventional imaging (integrated transesophageal echocardiography (TEE) and invasive measurements were utilized. The patients underwent diagnostic evaluation with the following advanced cardiac imaging modalities: SPECT and 4D-CT. Clinical characterization of patients is provided in Table 1, and more details are provided in Supplemental Table S1, which has already been published in our previous studies [22, 23]. Clinical follow-up involved standardized interviews, documentation from referring physicians, and hospital discharge summaries. A dedicated clinical event committee collected and adjudicated adverse events based on Valve Academic Research Consortium-2 criteria [22–24].

**Table 1:** Clinical characterization of the current study patient population. More detailed parameters are presented in Supplemental tables S2 and reference [23]

Clinical characteristics	All Participants (*n* = 263)	ATTR-CM Negative (*n* = 236)	ATTR-CM Positive (*n* = 27)	*P*-Value
Clinical				
Sex	M: 56.7% F: 43.3%	M: 84.6% F: 96.5%	M: 15.4% F: 3.5%	0.002
Age (y)	82.7 ± 4.6	82.4 ± 4.5	85.3 ± 4.6	0.002
BMI	26.6 ± 5.2	26.7 ± 5.2	26.0 ± 4.5	0.53
BSA (m^2)	1.85 ± 0.22	1.8 ± 0.2	1.9 ± 0.2	0.52
CAD	41.4%	40.7%	48.1%	0.46
Intervention imaging				
Aortic valve stenosis AVA echocardiography (cm^2)	0.7 ± 0.29	0.68 ± 0.26	0.89 ± 0.43	< 0.001
Laboratory				
Biomarker NT-proBNP (ng/L)	Median: 2834 (564–3490)	Median: 1140 (514–2947)	Median: 4462 (1925–6301)	< 0.001
Creatinine level (mmol/L)	95.3 ± 34.2	94.1 ± 33.3	105.6 ± 40.7	0.10
CT				
Cardiac CT calcium score aortic valve	Median: 2481 (1642–3750)	Median: 2545 (1675–3757)	Median: 2213 (1285–3659)	0.86
Cardiac CT Time gap to scintigraphy	Median: 1 (0–1)	Median: 1 (0–1)	Median: 1 (0–1)	0.37
Dose-length product (mG · cm)	973 ± 415.6	959.3 ± 422.6	1088.1 ± 336.4	0.13
Contrast agent dose (mL)	86.7 ± 12.7	86.8 ± 12.7	86.3 ± 13.3	0.85
Echocardiography				
LVEF (%)	53.7 ± 12.0	54.3 ± 12.1	50.2 ± 11.2	0.36
LV mass (g)	223 ± 70.2	217 ± 70.3	267 ± 55.2	0.06
LV mass index (g/m^2)	120 ± 36.9	117 ± 37.6	141 ± 24.9	0.049
LV septal thickness (mm)	13.6 ± 2.8	13.4 ± 2.8	14.5 ± 2.8	0.38
LV posterior wall thickness (mm)	11.6 ± 2.2	11.4 ± 2.2	13.1 ± 1.8	0.046
CT Global Strain (%)				
LV GLS	-14.3 ± 4.7	-14.7 ± 4.8	-11.2 ± 3.2	< 0.001
LV GRS	49.5 ± 25.4	50.2 ± 25.8	42.9 ± 21.0	0.16
LV GCS	-17.6 ± 6.3	-17.9 ± 6.4	-15.3 ± 5.2	0.04
RV GLS	-18.4 ± 7.2	-18.7 ± 7.2	-16.2 ± 6.9	0.09
LA GLS	14.2 ± 9.9	14.9 ± 10	8.3 ± 7.2	0.001

### ATTR-CM diagnosis

As part of the ATTR-AS (NCT04061213) study, all patients underwent [^99m^Tc]-3,3-diphosphono-1,2-propanodicarboxylic acid ([^99m^Tc]-DPD) scintigraphy for ATTR-CM screening [22, 23]. Approximately 3 h post intravenous injection of 700 ± 70 MBq [^99m^Tc]-DPD, whole-body planar images were acquired (15 cm/min) using a dual-head hybrid SPECT/CT system (Intevo; Siemens Healthineers) equipped with low-energy high-resolution (LEHR) collimators [22, 23]. The images were reconstructed using a high-order low-pass Butterworth filter (order of 5) and a zoom of 1.0, using a 256 × 256 matrix size [22, 23]. Following planar imaging, a SPECT/CT scan of the thorax was carried out using a step-and-shoot method adjusted for body contour (32 steps each 30 s, zoom of 1.0, 256 matrix size) [22, 23]. Then, SPECT images were reconstructed using an iterative algorithm (OSEM, 4 subsets, 8 iterations), supplemented by a 12-mm Gaussian filter [22, 23]. Additionally, a low-dose CT scan was conducted for attenuation correction, using 130 kV with CareDose, a pitch of 1.2, a rotation time of 0.6 s, and a collimation of 16 × 0.6 [22, 23]. The [^99m^Tc]-DPD scintigraphy results were interpreted as positive for participants exhibiting moderate to high myocardial tracer uptake (Perugini grade 2 or 3) and negative for those with no or low uptake (Perugini grade 0 or 1), as assessed by nuclear medicine physicians and cardiac imaging cardiologist (F.C., A.R., and C.G.) all with > 10 years of experience in nuclear cardiology [22, 23]. More information on data was provided in [22, 23].

### Data preparation and image processing

Advanced image processing techniques were utilized to extract CT strain and LV mass and function information from 4D contrast-enhanced CT (CCT) images; more details on the acquisition and processing of 4D CCT were previously published [22, 23]. Radiomics features were extracted from the left ventricle (LV) myocardium using CT images, including non-contrast images and images from the diastolic and systolic phases of contrast-enhanced 3D-CT. Delta radiomics were calculated using the diastolic and systolic phases of CCT. Segmentation of the LV was initially provided by an automatic approach and subsequently evaluated and modified as needed for different images. Various radiomics features, including intensity, shape, and second and higher-order features, were extracted using the Image Biomarker Standardization Initiative (IBSI) [25, 26] consensus Python library [27] with bin discretization set to 64 and an isotropic voxel size of 1 mm³.

We extracted the following number of features from each modality: 101 features from clinical, 13 from laboratory, 18 from ECG, 34 from echocardiography, 34 from invasive measurements, 6 from interventional imaging, 76 from CT strain, and 420 from radiomics (end-systolic, end-diastolic, non-contrast, and delta phases). The dataset was initially split into a training (70%) and a hold-out test set (30%) with stratification regarding the ATTR-CM status. Missing data within the dataset was imputed using an iterative imputation technique (applying a round-robin approach).

### Machine-learning algorithm

Z-score normalization was applied to the features to ensure uniformity in scale. Features exhibiting low variance (below a threshold of 0.99) were discarded. Subsequently, features with high correlation (Pearson correlation coefficient higher than 0.95) were grouped, and only the most predictive feature from each group was retained. Following this initial preprocessing, various feature selection algorithms, including Recursive Feature Elimination (RFE), Univariate Ranking (UniVa), and Minimum Redundancy Maximum Relevance (MRMR), were employed on the feature set to select informative features. Using the selected features, a variety of classifier models were trained, including logistic regression (LR), support vector machine (SVM), random forest (RF), AdaBoost, and XGBoost.

### Parameters and hyperparameters optimization

All ML model development steps, including preprocessing and feature selection, were performed exclusively on the training set and validation (70%) to process and select important features. Using these selected features, classifier parameters and hyperparameters were optimized using grid search on the training set to build the optimized model. Subsequently, the developed models were evaluated on the hold-out test set. This approach ensured that there was no possibility of information leakage between the training and test sets. The entire process (from data splitting to model evaluation) was repeated 100 times with a random seed to assess the robustness of the models.

### Evaluation and statistics

The SHAP (SHapley Additive exPlanations) model is utilized to interpret the outputs of ML models, providing insights into the contribution of each feature to the ATTR-CM prediction. The performance of these models was assessed using different metrics, including balanced accuracy (sensitivity + specificity)/2), receiver operating characteristic area under the curve (ROC-AUC), sensitivity, and specificity. The Mann–Whitney U test was employed to evaluate differences in performance metrics for statistical comparison across different models and modalities. Additionally, Kaplan-Meier survival curves were plotted for the diagnostic model output and their features to visualize the impact on patient outcomes over time, with p-values calculated from the log-rank (LR) test to determine the statistical significance of observed differences. All ML was implemented by Scikit-learn in the Python programming language [28], and all models and code are publicly available in the GitHub repository (https://github.com/AI-in-Cardiovascular-Imaging/ML_pipeline_tabular).

## Results

### Study population

Out of 489 patients initially assessed, 91 were ineligible, and 83 did not consent [22, 23]. Thus, 315 patients consented and were enrolled [22, 23]. From this cohort, 51 were excluded due to the absence of 4D-CCT, and one was excluded due to lack of correct image phases [22, 23]. Finally, 263 patients (83 ± 4.6years, 114 females) who underwent [^99m^Tc]-DPD scintigraphy and had available data from multiple modalities, including 4D-CCT, were included in the analysis [22, 23]. ATTR-CM was confirmed in 27 (10.3%) of these patients [22, 23]. Among those diagnosed with ATTR-CM, 22 underwent genetic testing, which revealed that 21 (95%) had wild-type ATTR, and 1 (5%) exhibited a transthyretin mutation (Val40Met) [22, 23]. The mean (standard deviation) follow-up for all causes of mortality and cardiovascular mortality was 13 (5) months, and it was available for 218 patients (23 ATTR-CM).

### Diagnostic performance

**Fig. 2:**
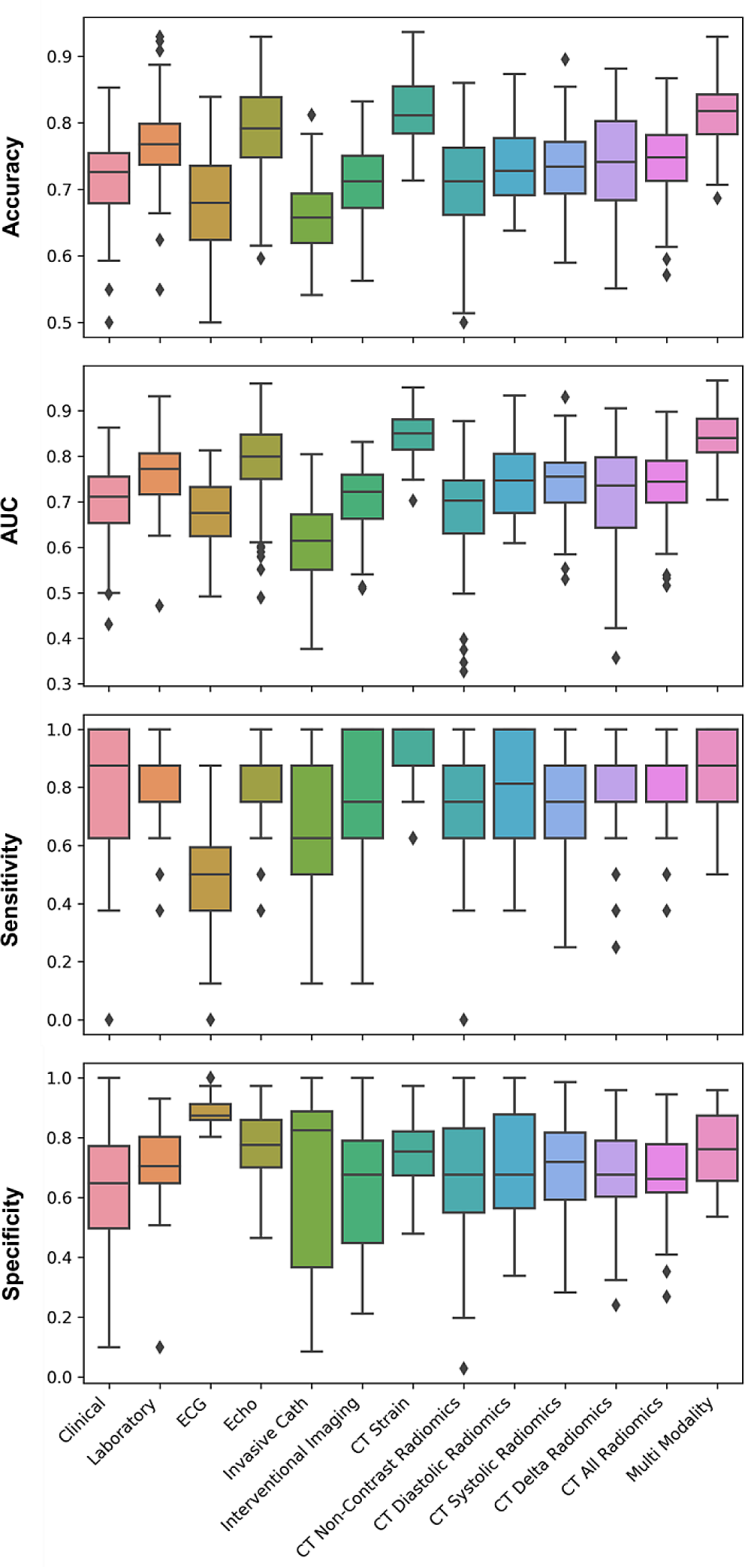
Comparative analysis of different metrics, including Accuracy, AUC, Sensitivity, and Specificity for the best-performing models in each modality, evaluated across 100 iterations. Clinical: RFE + LR, Laboratory: UniVa + LR, ECG: RFE + AdaBoost, Echo: UniVa + SVM, Invasive Cath: MRMR + LR, Interventional Imaging: UniVa + LR, CT Non-Contrast Radiomics: RFE + LR, CT Diastolic Radiomics: UniVa + LR, CT Systolic Radiomics: UniVa + LR, CT Delta Radiomics: UniVa + LR, CT All Radiomics: UniVa + LR, CT Strain: RFE + LR, Multi-Modality: RFE + LR. Classifiers include Logistic Regression (LR), Support Vector Machine (SVM), Random Forest (RF), AdaBoost (AdaBo), and eXtreme Gradient Boosting (XGB). Feature selection methods featured are Recursive Feature Elimination (RFE), Univariate Analysis (UniVa), and Minimum Redundancy Maximum Relevance (MRMR)

Figure 2 summarizes the performance of the diagnostic modalities (the best-performing ML algorithm in each modality) evaluated through different metrics as detailed in supplemental Table S2. Supplemental Figs. S1–S4 provide the p-values for comparing these modalities across different metrics.

### Diagnostic performance of conventional first-line diagnostic tests

ECG data showed a low performance with a mean (standard deviation) ROC-AUC of 0.67 (0.08) (sensitivity of 0.45 (0.16), and specificity of 0.89 (0.04)). Clinical data demonstrated a moderate discriminatory performance with ROC-AUC of 0.70 (0.09) (sensitivity of 0.81 (0.18) and specificity of 0.63 (0.18)). The laboratory data showed moderate results, with a ROC-AUC value of 0.76 (0.07) (sensitivity of 0.82 (0.13) and specificity of 0.72 (0.11)). Echocardiography resulted in acceptable performance with ROC-AUC of 0.79 (0.09) (sensitivity of 0.80 (0.15) and specificity of 0.78 (0.11)).

### Diagnostic performance of the CT radiomics

In the CT radiomics, CT non-contrast radiomics showed the lowest performance with a mean (standard deviation) ROC-AUC of 0.68 (0.11) (sensitivity of 0.76 (0.19), specificity of 0.66 (0.21)). The performances of CT diastolic, CT systolic, and CT delta radiomics were similar, with no statistically significant differences between the metrics of diagnostic performance (p-value > 0.05). Although the combined evaluation of all CT radiomics features resulted in a mean (standard deviation) of ROC-AUC of 0.74 (0.07) (sensitivity of 0.81 (0.14), specificity of 0.68 (0.13)), it did not outperform the individual systolic and diastolic radiomics (p-value > 0.05).

### Diagnostic performance of the CT strain

The highest discriminatory performances were observed for CT strain, yielding the highest diagnostic accuracy compared to other modalities. CT strain achieved the highest ROC-AUC of 0.85 (0.05) (sensitivity of 0.90 (0.11), specificity of 0.74 (0.11)).

### Diagnostic performance of invasive modalities

The lowest performances were observed for Invasive Cath (ROC-AUC of 0.61 (0.09), sensitivity of 0.64 (0.25), and specificity of 0.67 (0.28)). Interventional Imaging showed a ROC-AUC of 0.70 (0.08), sensitivity of 0.79 (0.18), and specificity of 0.63 (0.19).

### Diagnostic performance of the multi-modality

The multi-modality approach in which features from different diagnostic modalities were jointly considered, yielded a high mean (standard deviation) ROC-AUC of 0.84 (0.06) (sensitivity of 0.87 (0.13), specificity of 0.76 (0.12)). This is comparable to CT strain, indicating that while combining features can achieve high diagnostic performance, it does not statistically significantly outperform CT strain.

### The best model and modality for identifying ATTR-CM in patients with severe AS

Between different modalities, four models from CT strain (Manual + LR, RFE + LR, UniVa + AdaBo, and MRMR + SVM) and one model from multi-modality (RFE + LR) showed the highest performances, with no statistically significant differences between these models.

### ROC and heatmap plots in different models and modalities

Figures 3 and 4 present the ROC curves of the top three models across different modalities. Supplemental Figs. S5–S17 provide ROC curves for each model for 100-time repetition. Supplemental Figs. S18–S25 present heatmaps that compare these models in terms of p-values for different metrics across each modality. Figure 5 shows heatmaps of different feature selection and classifier combinations for different metrics within the echocardiography, CT strain, and multi-modality model. The corresponding heatmaps for the rest of the modalities examined are shown in supplemental Figs. S26–S35.

**Fig. 3:**
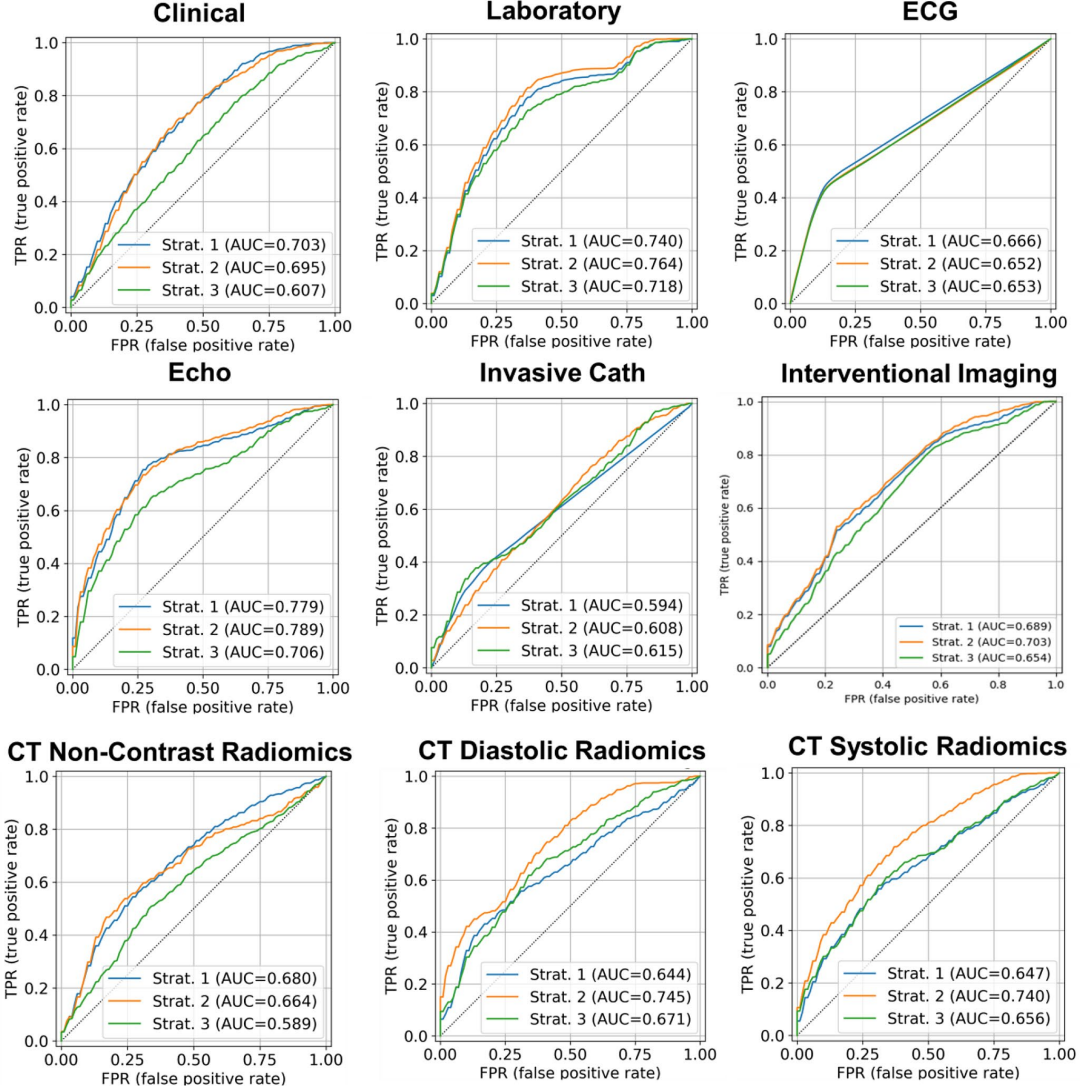
ROC curve of best-performing models in each modality. Clinical: Strat. 1 (RFE + LR), Strat. 2 (UniVa + LR), Strat. 3 (MRMR + LR); Laboratory: Strat. 1 (RFE + LR), Strat. 2 (UniVa + LR), Strat. 3 (MRMR + LR); ECG: Strat. 1 (RFE + AdaBo), Strat. 2 (UniVa + AdaBo), Strat. 3 (MRMR + AdaBo); Echo: Strat. 1 (RFE + LR), Strat. 2 (UniVa + SVM), Strat. 3 (MRMR + LR); Invasive Cath: Strat. 1 (RFE + AdaBo), Strat. 2 (UniVa + LR), Strat. 3 (MRMR + LR); Interventional Imaging: Strat. 1 (RFE + LR), Strat. 2 (UniVa + LR), Strat. 3 (MRMR + LR); CT Non-Contrast Radiomics: Strat. 1 (RFE + LR), Strat. 2 (UniVa + LR), Strat. 3 (MRMR + LR); CT Diastolic Radiomics: Strat. 1 (RFE + LR), Strat. 2 (UniVa + LR), Strat. 3 (MRMR + LR); CT Systolic Radiomics: Strat. 1 (RFE + LR), Strat. 2 (UniVa + LR), Strat. 3 (MRMR + LR). Classifiers include Logistic Regression (LR), Support Vector Machine (SVM), Random Forest (RF), AdaBoost (AdaBo), and eXtreme Gradient Boosting (XGB). Feature selection methods featured are Recursive Feature Elimination (RFE), Univariate Analysis (UniVa), and Minimum Redundancy Maximum Relevance (MRMR)

**Fig. 4:**
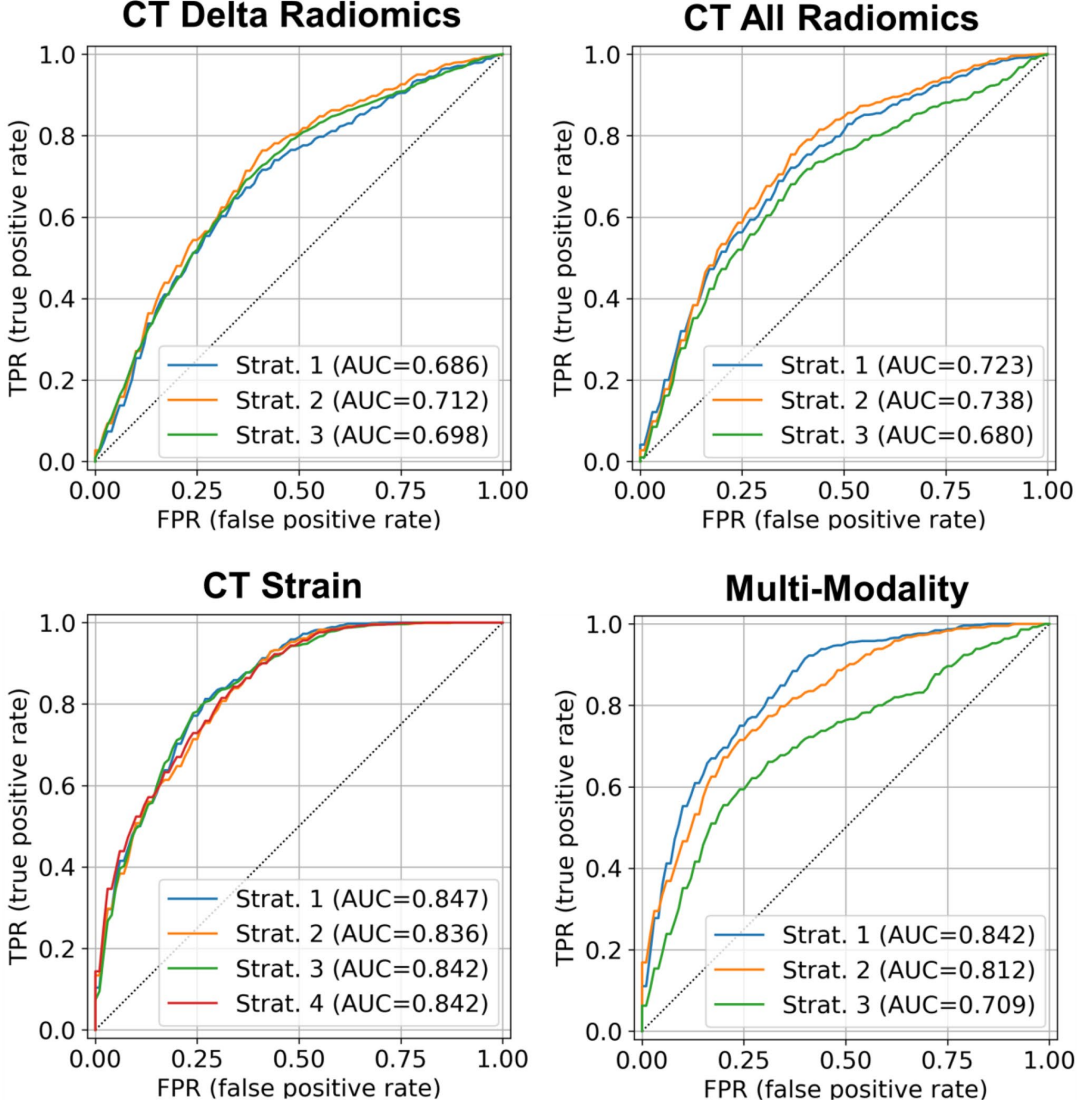
CT Delta Radiomics: Strat. 1 (RFE + LR), Strat. 2 (UniVa + LR), Strat. 3 (MRMR + AdaBo); CT All Radiomics: Strat. 1 (RFE + LR), Strat. 2 (UniVa + LR), Strat. 3 (MRMR + LR); CT Strain: Strat. 1 (Manual + LR), Strat. 2 (RFE + LR), Strat. 3 (UniVa + AdaBo) Strat. 4 (MRMR + SVM); Multi-Modality: Strat. 1 (RFE + LR), Strat. 2 (UniVa + LR), Strat. 3 (MRMR + LR). Classifiers include Logistic Regression (LR), Support Vector Machine (SVM), Random Forest (RF), AdaBoost (AdaBo), and eXtreme Gradient Boosting (XGB). Feature selection methods featured are Recursive Feature Elimination (RFE), Univariate Analysis (UniVa), and Minimum Redundancy Maximum Relevance (MRMR)

**Fig. 5:**
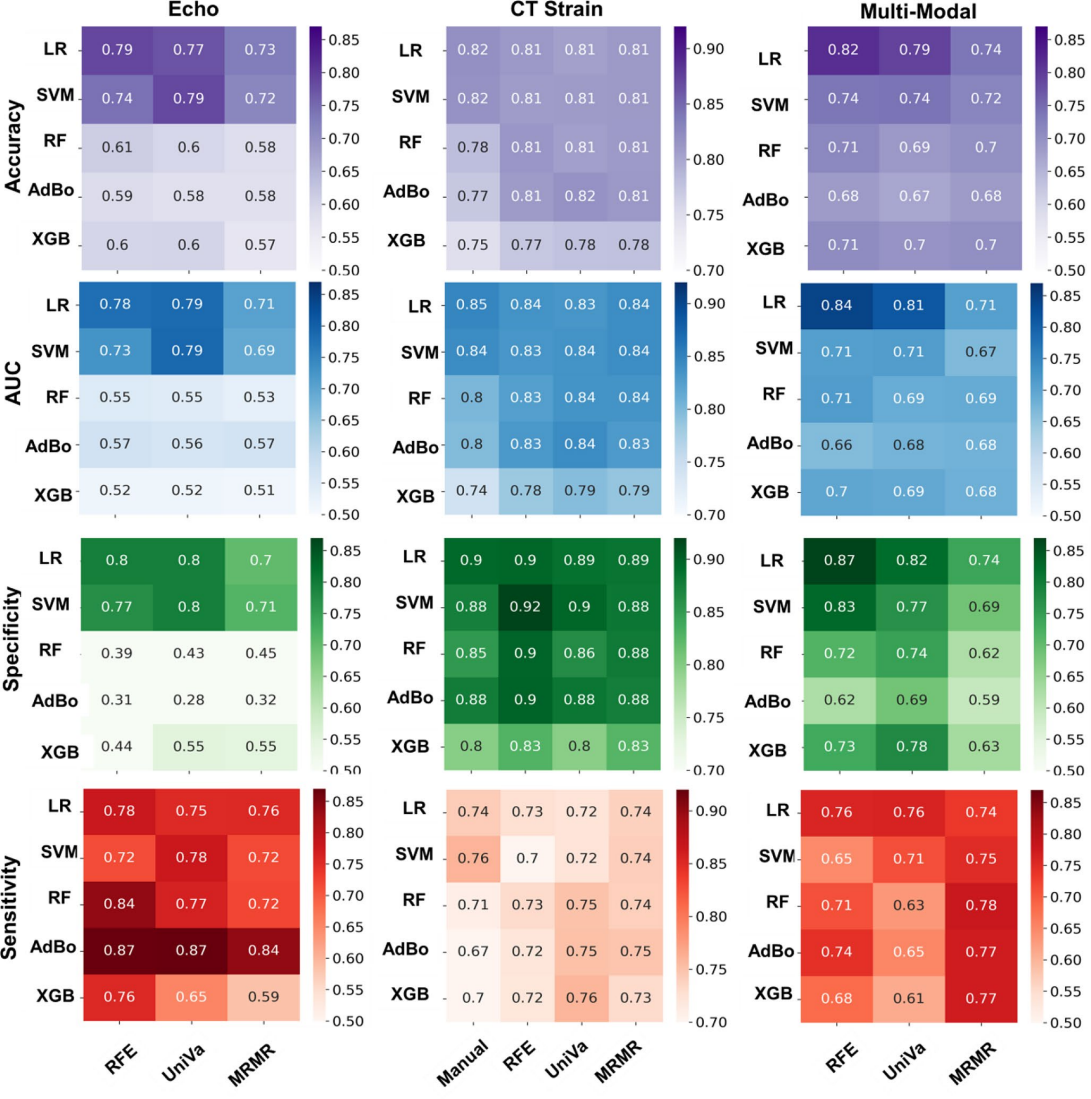
Heat maps displaying various metrics for echocardiography (Echo), CT strain, and Multi-Modal data. Classifiers include Logistic Regression (LR), Support Vector Machine (SVM), Random Forest (RF), AdaBoost (AdaBo), and eXtreme Gradient Boosting (XGB). Feature selection methods featured are Recursive Feature Elimination (RFE), Univariate Analysis (UniVa), and Minimum Redundancy Maximum Relevance (MRMR)

### Interpretability in top models

**Fig. 6:**
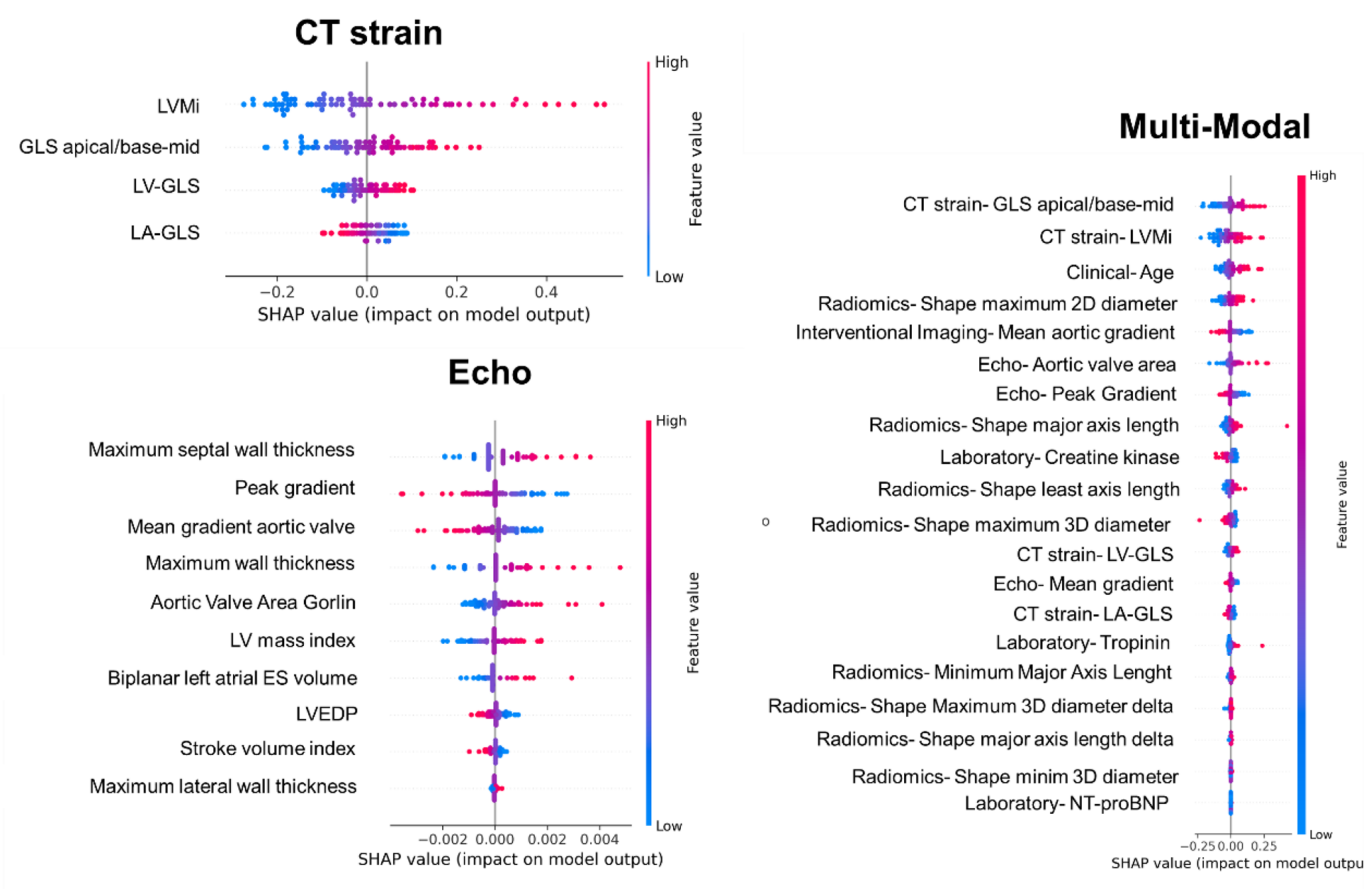
SHAP (SHapley Additive exPlanations) summary plot displaying the impact of various features across Echo, CT Strain, and Multi-Modality models. This visualization highlights the contribution of individual features to each model’s predictive performance for ATTR-CM detection. LVMi: Left ventricular mass (end-diastolic) index to gr/m2, GLS apical/base-mid: Left ventricular global longitudinal strain apical /base and mid, LV-GLS: Left ventricular global longitudinal strain (%), LA-GLS: Left atrial Global longitudinal strain (%), Mean gradient aortic valve: Mean gradient aortic valve [mmHg], Maximum Septal Wall Thickness: maximum septal wall thickness of the left ventricle [mm], Peak Gradient: peak pressure gradient of the aortic valve [mmHg], Mean Gradient: mean pressure gradient of the aortic valve [mmHg], Biplanar Left Atrial ES volume: Biplanar LAESVi [ml / BSA in m2], LV mass index: left ventricular mass index LVMi [g/m2], LVEDP: estimated left ventricular end-diastolic pressure [mmHg], Maximum lateral wall thickness: maximum lateral wall thickness of the left ventricle [mm], NT-proBNP: N-terminal pro B-type natriuretic peptide [pg/ml]

Figure 6 presents the SHAP summary of the top three models, including echocardiography, CT strain, and multi-modality, andsupplemental Figs. S36–S38 present the other top models in each modality. The SHAP plots illustrate the relative importance of each feature in each model, providing insights into the decision-making processes underlying the feature-outcome relationships in each modality toward ML model interpretability. For instance, in the echocardiography model, features such as the mean gradient of the aortic valve and maximum septal wall thickness had the highest impact. Decreasing the mean gradient of the aortic valve and increasing the maximum septal wall thickness positively influenced the ML model’s output toward ATTR-CM positive diagnosis. In the CT strain model, increasing feature values like LV global longitudinal strain apical /base and mid (GLS apical/base-mid), LV global longitudinal strain (LV-GLS, %), end-diastolic LV mass index (LVMi, g/m^2^) and decreasing in left atrial global longitudinal strain (LA-GLS, %) has a positive influence on model output. In the multi-modality model, in addition to the CT strain and echocardiographic features, other variables such as age, radiomics features, and laboratory data contributed to the model’s outcomes.

### Prognostication information of diagnostic features

**Fig. 7:**
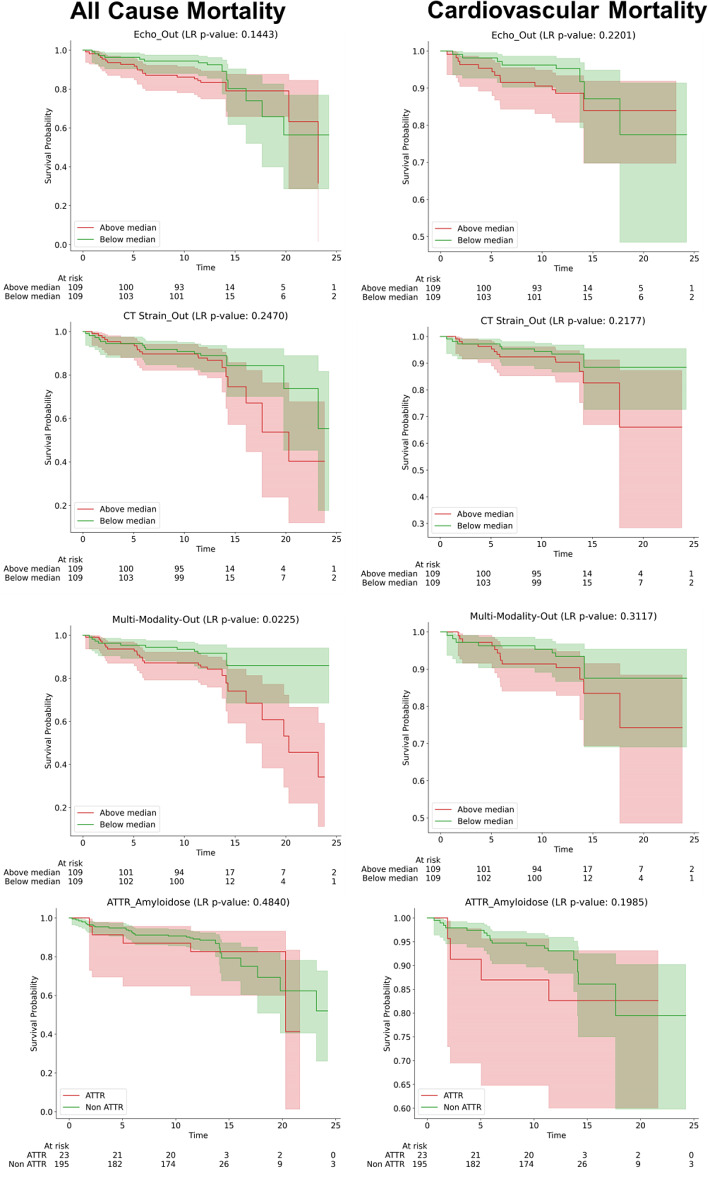
Kaplan-Meier curves for all-cause mortality and cardiovascular mortality, stratified based on the median value of the outputs of the diagnostic model for ATTR-CM and the ground truth of ATTR-CM. The stratification categories are above and below the median ML output values, illustrating survival probabilities over time for each group with the x-axis in months

Prognostication based on ATTR-CM status did not show significant prognostic information (indicated by an LR p-value > 0.05). Among the different diagnostic ML models, only the multi-modality model provided prognostic information (p-value = 0.007) for distinguishing between low- and high-risk groups for all-cause mortality (Fig. 7). Selected features from different modalities provided valuable prognostic information to differentiate between low and high-risk groups in all causes and cardiac-specific mortality, as illustrated in the Kaplan-Meier plots of these features in Supplemental Figs. S39–S40.

## Discussion

In the current study, we comprehensively evaluated the performance of ML approaches based on preprocedural and routinely collected data from different contemporary diagnostic modalities to predict ATTR-CM in patients with severe AS planned for TAVI. These modalities included clinical assessment, ECG and echocardiography, as well as more advanced imaging processing techniques such as CT strain and CT radiomics. We employed a wide range of standardized ML algorithms to predict ATTR-CM using single and multi-modality data. While echocardiography demonstrated good performance, CT strain exceeded it in accuracy. The multi-modality model did not outperform the CT strain-only data. Specific features from different modalities provided prognostic information in severe AS patients for all-cause and cardiac-specific mortality. The presence of ATTR-CM was not shown to be an outcome predictor.

Current diagnostic standards for establishing an ATTR-CM diagnosis, such as biopsy or bone scintigraphy [8–10], introduce additional costs and burdens, and are not included in the standard clinical practice for patients with severe AS undergoing TAVI procedures [1–3]. Although scintigraphy is becoming the gold standard for ATTR-CM detection and provides prognostic information [29–31], it presents other challenges, such as radiation exposure and delays in diagnosis due to the low deployment of nuclear medicine centers. Moreover, scintigraphy imaging can yield negative results if amyloid deposition is minimal at the time of examination [1–3]. Therefore, ATTR-CM is likely underdiagnosed in this cohort [1–3], highlighting the need for non-invasive and cost-effective diagnostic tools [1]. Our study presents new evidence that utilizing ML to integrate preprocedural and routinely collected data aids in detecting concomitant ATTR-CM in patients with severe AS. Using the existing data for ATTR-CM detection could also enhance prognostication in this patient group.

Several studies have been employed to detect ATTR-CM using AI in different modalities [1, 32–34]. Previous studies using the echocardiographic images and DL model [32] and handcrafted echocardiographic parameters and ML [34] reported an average AUC of 0.87 (5-fold- cross-validation (CV)) and 0.82 (0.95, 0.76, 0.78, and 0.80 on the four external tests) in detecting cardiac amyloidosis and wild-type ATTR-CM, respectively. Another study [33] employed automated tools using ECG and echocardiography to detect wild-type ATTR-CM. The DL was externally tested at four centers, with 441 (AUC: 0.91), 369(AUC: 0.89), 229(AUC: 1.0), and 239(AUC: 0.96) patients [33]. In our study, using only the ECG modality did not yield good performance, whereas the echocardiographic modality provided results comparable to previous studies. Despite our limited dataset, focusing on patients with severe AS and overlapping symptoms made constructing high-performance models challenging. Thus, this should be considered when comparing study results, as model performance is influenced by the specific cohort used for development and evaluation, not just the metrics [35].

In a previous study [36], [^99m^Tc]-HMDP scintigraphy was utilized to detect ATTR-CM. They [36] focused on classifying ATTR-CM based on Perugini grades using DL models. They [36] reported an AUC of 0.87 for multiclass classification, 0.94 for the binary comparison of grade < 2 vs. grade ≥ 2, and 0.89 for grade < 3 vs. grade 3 using a 5-fold CV. Another study [37] developed a DL model using scintigraphy images ([^99m^Tc]-DPD/[^99m^Tc]-HMDP) to detect a Perugini grade of ≥ 2 in ATTR-CM patients. They reported an AUC of 0.99 in both the development and external test phases, demonstrating high diagnostic accuracy. In our study, we did not use scintigraphy as an input for detecting ATTR-CM but rather as the ground truth, with most positive cases confirmed by pathology and genetic tests. As scintigraphy is not routinely implemented in clinical practice among patients with severe AS undergoing TAVI due to additional cost, our study demonstrates the feasibility of detecting ATTR-CM in this cohort using preprocedural and routinely collected data with good performance. This model could be used for initial screening with available data for this cohort, allowing suspected cases to undergo scintigraphy for confirmation.

The diagnosis performance of ML and DL was investigated [38] using CMR images, and they reported an AUC of 0.98 for DL and 0.95 for ML for ATTR-CM detection. Other [39] conducted a study using CMR sequences to diagnose cardiac amyloidosis automatically. They [39] employed binary classification approaches to analyze single 2D slices using DL and used averaged voting across all slices for comprehensive patient-wise analysis. They reported AUC scores of 0.96 for LGE, 0.93 for MOLLI, and 0.91 for CINE. In our study, we did not use the CMR dataset due to its limited availability for TAVI patients. However, future studies could integrate this modality to evaluate new model performance.

In a recent study [40] contrast-enhanced CT radiomics features of 30 patients were used to differentiate cardiac amyloidosis from severe AS. Due to the small data size, they used a leave-one-out CV and reported accuracy, sensitivity, and specificity of 0.93. In another study [41] CT radiomics features evaluated for detecting cardiac amyloidosis in AS patients who underwent TAVI. Using a 7-fold CV, they reported an AUC of 0.92 for radiomics and 0.96 when combining radiomics with clinical information. Our study evaluated the performance of various CT radiomics features, achieving a moderate AUC of 0.75. Compared to previous radiomics studies, our dataset was larger, and we enhanced the reliability of our results by repeating the entire process 100 times with random seeds to avoid any bias in the chosen test set. This approach is essential in ML studies with small to medium-sized datasets because achieving high performance in a single repetition could be potentially due to a random split that favors easier cases in the test set, which may not realistically reflect real-world scenarios.

In each ML model, we used SHAP analysis to understand the top model’s decision-making. We observed that these features and decisions align with previous clinical findings, which makes the model more rational and reliable [1]. In the Echo, the ML model showed that decisions for detecting ATTR-CM are based on a combination of decreasing gradient, increasing wall thickness, and increasing LV mass and volume. The selected features and their behavior align with clinical symptoms in ATTR-CM patients, as the amyloid fibrils lead to an increased myocardial thickness, consequently decreasing the LV stroke volume [1, 6, 42]. While these features alone cannot fully represent ATTR-CM, their combined effects and the varying weights assigned to them could form a robust diagnostic model. While wall thickness, especially maximum septal wall thickness, was a key feature in the echocardiography model, it did not contribute significantly in the multimodality model, where other features were more influential. A recent study suggested [43] that wall thickness is not correlated with ATTR-CM, which aligns with our finding that in the presence of other features, such as CT strain, the importance of wall thickness decreases.

In the CT strain modality ML modeling, we used automated and manual feature selection based on our previous study [23]. However, there was no statistically significant difference between the manually selected and the automated ones, and we achieved the highest performance using CT strain analysis for different modalities. Previously [23], we employed conventional standard statistical methods to evaluate models, reporting different cutoffs in AUC of 0.89 with internal bootstrapping (sensitivity of 0.96 and 0.77, and specificity of 0.58 and 0.85). However, in the current study using standardized ML approaches, we achieved an ROC-AUC of 0.85 ± 0.05 with a sensitivity of 0.90 ± 0.11 and a specificity of 0.74 ± 0.11. Although the AUC is slightly lower than the previous study [23], the ML model improved the performance of ATTR-CM detection by considering both sensitivity and specificity. High sensitivity and low specificity could be impractical in clinical settings due to the high number of false positives; thus, a model simultaneously minimizes false positives and false negatives is preferable. Additionally, conventional statistical models often risk information leakage through internal bootstrapping and data splitting, potentially inflating performance metrics. In contrast, our standardized ML development approach avoided any information leakage, leading to more realistic and superior performance (considering both sensitivity and specificity simultaneously) with CT strain compared to our previous studies [23]. Our CT strain models demonstrated that combining features indicative of myocardial contractility and wall motion abnormalities could create a high-performance predictive model for detecting ATTR-CM. Although CT strain may not be routinely collected, our analysis indicates that the top contributing features in the multimodality model are derived from CT strain. Attempts to build a model using only routinely collected data did not yield satisfactory performance, showing the importance of CT strain in accurate diagnosis of ATTR-CM. Considering advancements in CT scanner technology, which significantly reduce acquisition time and radiation dose [44], as well as the necessity of pre-TAVI CT images and previous guideline [45] recommendation, 4D-CT could potentially be acquired routinely in the future. This model could seamlessly be integrated into clinical routine, providing an additional tool that uses available information to identify and alert clinicians to high-risk patients for ATTR-CM as it might change the clinical decision for TAVI versus surgical therapy.

Although the multi-modality model did not outperform the CT strain-only models, it incorporates additional features such as the radiomics of the LV myocardium, where an increase in value tends to indicate a diagnosis of ATTR-CM. Moreover, other features like age/troponin and creatine kinase (CK) were found to have positive and negative impacts on the model’s output, respectively. Additionally, selected features from various modalities were useful in differentiating between low and high-risk groups for all-cause and cardiac-related mortality. Although overall prognostic assessments based on ATTR-CM did not show significant performance, the multi-modality model output was effective in distinguishing between different mortality risk groups. This shows the potential benefits of integrating multiple diagnostic modalities to enhance the accuracy of prognostic assessments and provide new biomarkers. A DL model developed [46] for ATTR-CM detection in scintigraphy, demonstrated that the outputs of the diagnostic models could serve as markers for prognosis and discriminate between high and low-risk groups for overall mortality. Another study [2] showed that a diagnostic model using ECG in severe AS patients undergoing TAVI indicated that the diagnostic DL model’s output could predict all-cause mortality, major adverse cardiac events, and hospitalization due to heart failure. Our results align with these earlier studies, demonstrating the potential of diagnostic models to prognosticate and offer new biomarkers. The Kaplan-Meier curves are plotted based on the output of the models, stratified by the median value of the diagnostic model for ATTR-CM detection. We hypothesize that false positive cases, which may include patients with severe conditions resembling ATTR-CM, lead to worse outcomes due to impaired cardiac function. This suggests that our model may capture additional prognostic information not accounted for in the binary classification of ATTR-CM from different features of various modalities. Future studies should evaluate the prognostication performance of this model on TAVI patient cohorts.

In this study, we implemented multiple ML algorithms that yielded different performance results, which may arise from the specific characteristics of each model. The LR often outperformed other models in different modalities, making it advantageous for clinical use due to its simplicity and greater interpretability. However, in some cases, such as with CT strain, which is the best modality for ATTR-CM detection, models like AdaBoost, SVM, and RF performed similarly well. The lower performance of complex models like XGBoost could be due to overfitting in the training set, given their high number of parameters. By using an untouched test set for evaluation, we ensured an unbiased comparison and selected the most reliable performing model.

Gathering a comprehensive dataset encompassing multiple modalities for assessing ATTR-CM in AS is highly challenging, as the concordance of ATTR-CM is not routinely evaluated in clinical practice. Although our dataset may be considered medium-sized compared to previous clinical ML studies in ATTR-CM [32, 38–41], the methodology we applied ensures that the generated results are robust and repeatable. Furthermore, the clinical objective of our study is not to replace scintigraphy with our ML model but to use the model to detect potential cases, which can then be confirmed through scintigraphy. This approach has the potential to enhance clinical workflow for AS patients undergoing TAVI procedures. One of the main limitations of this study is its reliance on a single-center and unbalanced dataset. However, we employed various approaches, including stratified splitting, avoiding any information leakage between the training and testing sets, and repeating the experiment with a random seed to provide more realistic and robust results. Although we tried data augmentation techniques such as SMOTE in the training set, it did not improve the model’s performance, and we continued with the original data. Moreover, we have made our code and model publicly available to support open-source practices and the reproducibility of the study. Domain shift in ML studies can occur due to variations in data acquisition methods (i.e. changing in the scanner), population characteristics, and changes over time. These shifts can impact model performance and should be carefully monitored, even in single-centre studies. Future studies should evaluate and validate our models’ performance in larger, prospective, and external datasets.

## Conclusion

In the current study, we implemented ML to evaluate the efficacy of various modalities for predicting ATTR-CM in patients with severe AS undergoing TAVI. While echocardiography, CT strains, and multi-modality demonstrated high diagnostic performance, with CT strain being the highest-performing modality, the multi-modality model did not outperform CT strain alone. Other modalities, including LV radiometric features on CT scans, showed moderate performance for detecting ATTR-CM. Moreover, some diagnostic features could provide more insights for prognostication in severe AS. Our study demonstrates that applying ML to routine pre-TAVI data can effectively detect concomitant ATTR-CM in patients with severe AS, presenting a potential alternative to scintigraphy or invasive biopsies.

## Electronic supplementary material

Below is the link to the electronic supplementary material.

Supplementary Material 1

## Data Availability

The datasets generated during and/or analyzed during the current study are available from the corresponding author upon reasonable request. All code and developed models are available in the following GitHub repository: https://github.com/AI-in-Cardiovascular-Imaging/ML_pipeline_tabular.
