# Supplementary material for: Multi-modality artificial intelligence-based transthyretin amyloid cardiomyopathy detection in patients with severe aortic stenosis
Source: Eur J Nucl Med Mol Imaging. 2024 Sep 23;52(2):485–500. doi: 10.1007/s00259-024-06922-4 (PMC11732884; doi:10.1007/s00259-024-06922-4)
Supplement: Supplementary file 1 — Supplementary Material 1 [file 259_2024_6922_MOESM1_ESM.docx]

**Supplemental Data**

**Supplemental Tables**

.

**Supplemental Table 1:** Clinical characterization from our previous study [1].

| Clinical characteristics | All Participants (n=263) | ATTR-CM Negative (n=236) | ATTR-CM Positive (n=27) | P-Value |
| --- | --- | --- | --- | --- |
| Clinical | | | | |
| Sex | M: 56.7%  F: 43.3% | M: 84.6%  F: 96.5% | M: 15.4%  F: 3.5% | 0.002 |
| Age (y) | 82.7 ± 4.6 | 82.4 ± 4.5 | 85.3 ± 4.6 | 0.002 |
| BMI | 26.6 ± 5.2 | 26.7 ± 5.2 | 26.0 ± 4.5 | 0.53 |
| BSA (m^2) | 1.85 ± 0.22 | 1.8 ± 0.2 | 1.9 ± 0.2 | 0.52 |
| Arterial hypertension | 87.5% | 86.9% | 92.6% | 0.52 |
| Diabetes mellitus | 28.5% | 29.2% | 22.2% | 0.44 |
| CAD | 41.4% | 40.7% | 48.1% | 0.46 |
| Prior PCI | 12.9% | 12.7% | 14.8% | 0.87 |
| Prior CABG | 7.6% | 7.6% | 7.4% | 0.97 |
| History of atrial fibrillation | 35.4% | 34.3% | 44.4% | 0.30 |
| Stroke and/or TIA | 15.2% | 15.3% | 14.8% | 0.99 |
| PPM implanted | 8.4% | 7.6% | 14.8% | 0.26 |
| History of polyneuropathy | 3.4% | 3.4% | 3.7% | 0.99 |
| History of spinal stenosis | 6.5% | 5.5% | 14.8% | 0.08 |
| History of carpal tunnel syndrome | 6.1%) | 5.1% | 14.8% | 0.07 |
| Dyspnea on exertion (NYHA II or III) | 91.6%) | 91.5% | 92.6% | 0.99 |
| Intervention imaging | | | | |
| Aortic valve stenosis AVA echocardiography (cm^2) | 0.7 ± 0.29 | 0.68 ± 0.26 | 0.89 ± 0.43 | <0.001 |
| LVEDP (mm Hg) | 19.7 ± 8.5 | 19.9 ± 8.7 | 17.5 ± 6.3 | 0.18 |
| Mean transvalvular gradient echocardiography (mm Hg) | 37.6 ± 16.1 | 39.0 ± 16.1 | 25.5 ± 9.6 | <0.001 |
| Laboratory | | | | |
| Biomarker NT-proBNP (ng/L) | Median: 2834 (564–3490) | Median: 1140 (514–2947) | Median: 4462 (1925–6301) | <0.001 |
| Creatinine level (mmol/L) | 95.3 ± 34.2 | 94.1 ± 33.3 | 105.6 ± 40.7 | 0.10 |
| eGFR (mL/min) | 61.5 ± 18.2 | 62.0 ± 18.1 | 57.2 ± 18.9 | 0.19 |
| eGFR <30 mL/min | 17 (6.5%) | 15 (6.4%) | 2 (7.4%) | 0.69 |
| CT | | | | |
| Cardiac CT calcium score aortic valve | Median: 2481 (1642–3750) | Median: 2545 (1675–3757) | Median: 2213 (1285–3659) | 0.86 |
| Cardiac CT Time gap to scintigraphy | Median: 1 (0–1) | Median: 1 (0–1) | Median: 1 (0–1) | 0.37 |
| Dose-length product (mG · cm) | 973 ± 415.6 | 959.3 ± 422.6 | 1088.1 ± 336.4 | 0.13 |
| Contrast agent dose (mL) | 86.7 ± 12.7 | 86.8 ± 12.7 | 86.3 ± 13.3 | 0.85 |
| Echocardiography | | | | |
| LVEF (%) | 53.7 ± 12.0 | 54.3 ± 12.1 | 50.2 ± 11.2 | 0.36 |
| LV EDV (mL) | 101 ± 30.5 | 101 ± 29.8 | 101 ± 36.0 | 0.98 |
| LV EDV/BSA (mL/m^2) | 54.3 ± 15.1 | 54.6 ± 15.0 | 53.1 ± 17.0 | 0.83 |
| LV mass (g) | 223 ± 70.2 | 217 ± 70.3 | 267 ± 55.2 | 0.06 |
| LV mass index (g/m^2) | 120 ± 36.9 | 117 ± 37.6 | 141 ± 24.9 | 0.049 |
| LV septal thickness (mm) | 13.6 ± 2.8 | 13.4 ± 2.8 | 14.5 ± 2.8 | 0.38 |
| LV posterior wall thickness (mm) | 11.6 ± 2.2 | 11.4 ± 2.2 | 13.1 ± 1.8 | 0.046 |

| Volumetric Assessments | | | | | |
| --- | --- | --- | --- | --- | --- |
| LV EDV (mL) | 161.1 ± 49.4 | 159.2 ± 48.0 | 177.8 ± 58.5 | 0.06 |  |
| LV EDV/BSA (mL/m^2) | 87.0 ± 23.3 | 86.1 ± 22.6 | 94.5 ± 28.0 | 0.07 |  |
| LV SV/BSA (mL/m^2) | 46.1 ± 12.4 | 46.5 ± 12.7 | 42.3 ± 9.1 | 0.09 |  |
| LV EF (%) | 54.9 ± 15.4 | 55.7 ± 15.4 | 47.8 ± 14.2 | 0.01 |  |
| LV EF <51% | 96 (36.5%) | 80 (33.9%) | 16 (59.3%) | 0.01 |  |
| LV mass (g) | 139.5 ± 39.8 | 135.8 ± 38.4 | 171.4 ± 38.4 | <0.001 |  |
| LV mass index (g/m^2) | 75.3 ± 19.0 | 73.4 ± 18.3 | 91.2 ± 18.5 | <0.001 |  |
| RV EDA (cm^2) | 26.3 ± 7.0 | 25.9 ± 6.9 | 29.3 ± 7.3 | 0.02 |  |
| RV FAC (%) | 33.9 ± 11.6 | 34.3 ± 11.6 | 29.9 ± 11.3 | 0.06 |  |
| LA ESV (mL) | 108 ± 38.5 | 107.4 ± 38.6 | 116.1 ± 37.6 | 0.27 |  |
| LA ESV/BSA (mL/m^2) | 59.1 ± 20.7 | 58.6 ± 20.6 | 62.7 ± 21.3 | 0.35 |  |
| LA EF (%) | 28.1 ± 17.9 | 29.3 ± 17.9 | 17.2 ± 14.1 | 0.001 |  |
| Maximal Wall Thickness (mm) | | | | | |
| Basal septal wall | 12.0 ± 2.3 | 11.8 ± 2.2 | 14.0 ± 2.1 | 0.002 |  |
| Midventricular septal wall | 9.3 ± 2.1 | 9.0 ± 1.8 | 11.2 ± 3.0 | 0.04 |  |
| Apical septal wall | 7.0 ± 1.5 | 6.9 ± 1.5 | 7.5 ± 1.8 | 0.15 |  |
| Basal lateral wall | 9.2 ± 1.6 | 9.1 ± 1.5 | 10.1 ± 1.9 | <0.001 |  |
| Midventricular lateral wall | 7.9 ± 1.4 | 7.8 ± 1.4 | 8.3 ± 1.5 | 0.02 |  |
| Apical lateral wall | 7.0 ± 1.4 | 6.9 ± 1.4 | 7.2 ± 1.6 | 0.08 |  |
| Global Strain (%) | | | | | |
| LV GLS | -14.3 ± 4.7 | -14.7 ± 4.8 | -11.2 ± 3.2 | <0.001 |  |
| LV GRS | 49.5 ± 25.4 | 50.2 ± 25.8 | 42.9 ± 21.0 | 0.16 |  |
| LV GCS | -17.6 ± 6.3 | -17.9 ± 6.4 | -15.3 ± 5.2 | 0.04 |  |
| RV GLS | -18.4 ± 7.2 | -18.7 ± 7.2 | -16.2 ± 6.9 | 0.09 |  |
| LA GLS | 14.2 ± 9.9 | 14.9 ± 10 | 8.3 ± 7.2 | 0.001 |  |
| Relative Apical Strain | | | | | |
| Longitudinal | 1.39 ± 0.38 | 1.37 ± 0.36 | 1.62 ± 0.46 | 0.002 |  |
| Radial | 1.39 ± 1.40 | 1.36 ± 1.37 | 1.66 ± 1.45 | 0.28 |  |
| Circumferential | 1.43 ± 0.53 | 1.41 ± 0.51 | 1.65 ± 0.67 | 0.03 |  |

| Modality | Strategy | FS | Classifier | AUC | Sensitivity | Specificity | Accuracy |
| --- | --- | --- | --- | --- | --- | --- | --- |
| Clinical | Strat. 1 | RFE | LR | 0.70±0.09 | 0.81±0.18 | 0.63±0.18 | 0.72±0.06 |
| Laboratory | Strat. 2 | UniVa | LR | 0.76±0.07 | 0.82±0.13 | 0.72±0.11 | 0.77±0.06 |
| ECG | Strat. 1 | RFE | AdaBost | 0.67±0.08 | 0.45±0.16 | 0.89±0.04 | 0.67±0.08 |
| Echo | Strat. 2 | UniVa | SVM | 0.79±0.09 | 0.80±0.15 | 0.78±0.11 | 0.79±0.07 |
| Invasive Cath | Strat. 3 | MRMR | LR | 0.61±0.09 | 0.64±0.25 | 0.67±0.28 | 0.66±0.06 |
| Interventional Imaging | Strat. 2 | UniVa | LR | 0.70±0.08 | 0.79±0.18 | 0.63±0.19 | 0.71±0.05 |
| CT Strain | Strat. 1 | RFE | LR | 0.85±0.05 | 0.90±0.11 | 0.74±0.11 | 0.82±0.05 |
| CT Non-Contrast Radiomics | Strat. 1 | RFE | LR | 0.68±0.11 | 0.76±0.19 | 0.66±0.21 | 0.71±0.08 |
| CT Diastolic Radiomics | Strat. 2 | UniVa | LR | 0.74±0.08 | 0.78±0.19 | 0.69±0.18 | 0.74±0.05 |
| CT Systolic Radiomics | Strat. 2 | UniVa | LR | 0.74±0.07 | 0.76±0.16 | 0.7±0.16 | 0.73±0.06 |
| CT Delta Radiomics | Strat. 2 | UniVa | LR | 0.71±0.11 | 0.80±0.17 | 0.67±0.15 | 0.74±0.08 |
| CT All Radiomics | Strat. 2 | UniVa | LR | 0.74±0.07 | 0.81±0.14 | 0.68±0.13 | 0.75±0.05 |
| Multi-Modality | Strat. 1 | RFE | LR | 0.84±0.06 | 0.87±0.13 | 0.76±0.12 | 0.82±0.05 |

**Supplemental Table 2:** Comparative analysis of different metrics, including Accuracy, AUC, Sensitivity, and Specificity for the best-performing models in each modality, evaluated across 100 iterations. Clinical: RFE+LR, Laboratory: UniVa+LR, ECG: RFE+AdaBoost, Echo: UniVa+SVM, Invasive Cath: MRMR+LR, Interventional Imaging: UniVa+LR, CT Non-Contrast Radiomics: RFE+LR, CT Diastolic Radiomics: UniVa+LR, CT Systolic Radiomics: UniVa+LR, CT Delta Radiomics: UniVa+LR, CT All Radiomics: UniVa+LR, CT Strain: RFE+LR, Multi-Modality: RFE+LR.

**Supplemental Figures**


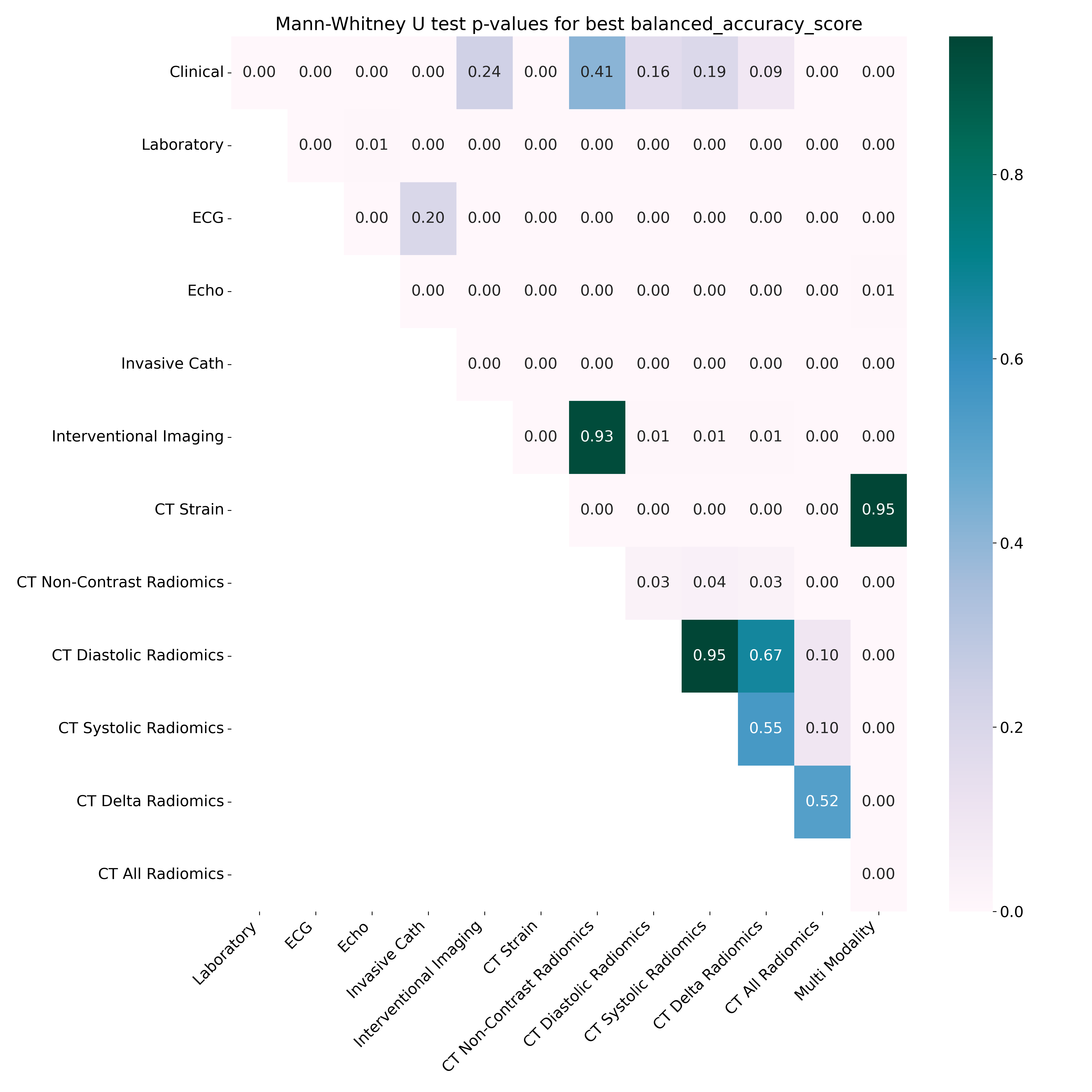


**Supplemental Figure 1:** Heat map visualizing the Mann-Whitney U-test results for comparing the accuracy of different modalities.


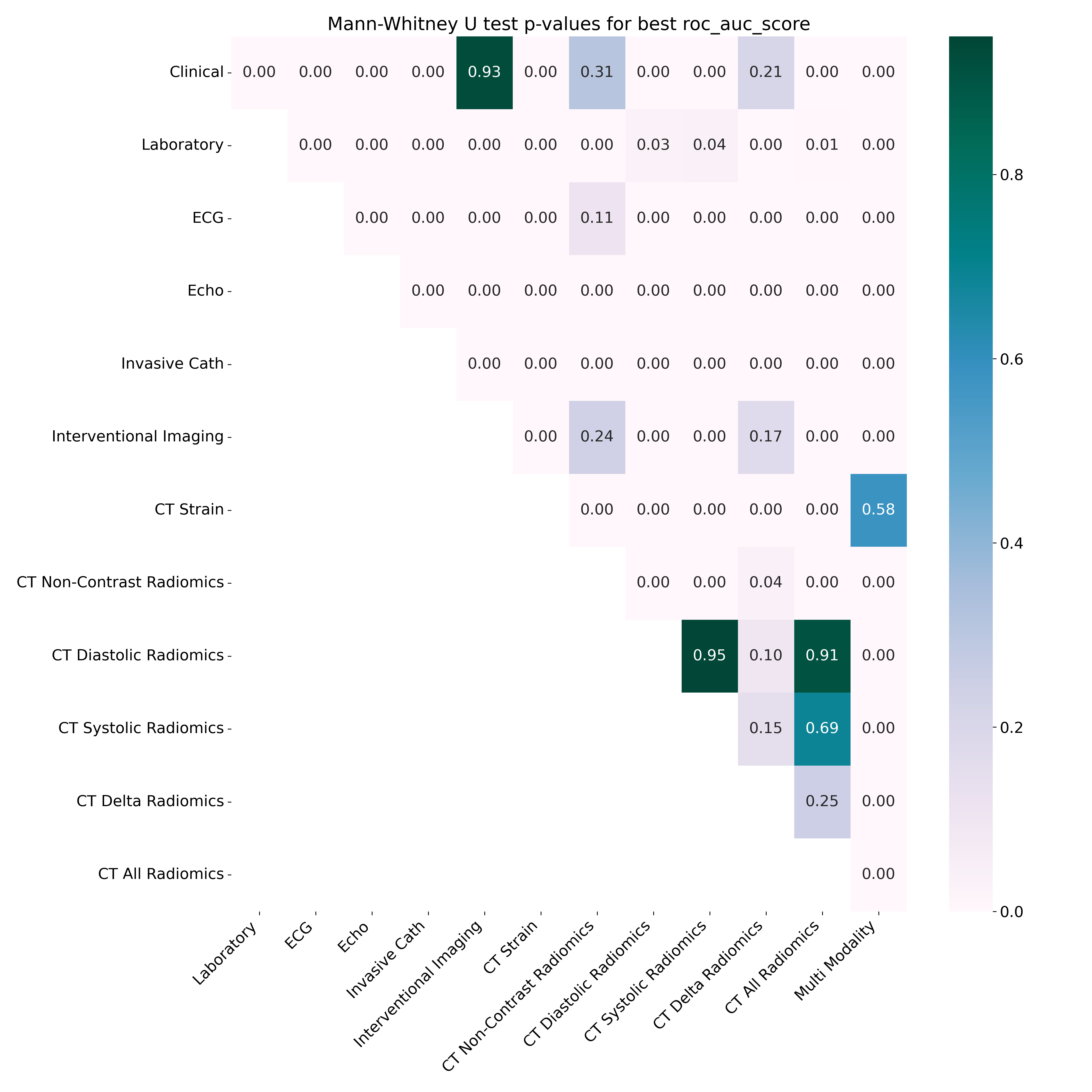


**Supplemental Figure 2:** Heat map visualizing the Mann-Whitney U-test results for comparing the AUC of different modalities.


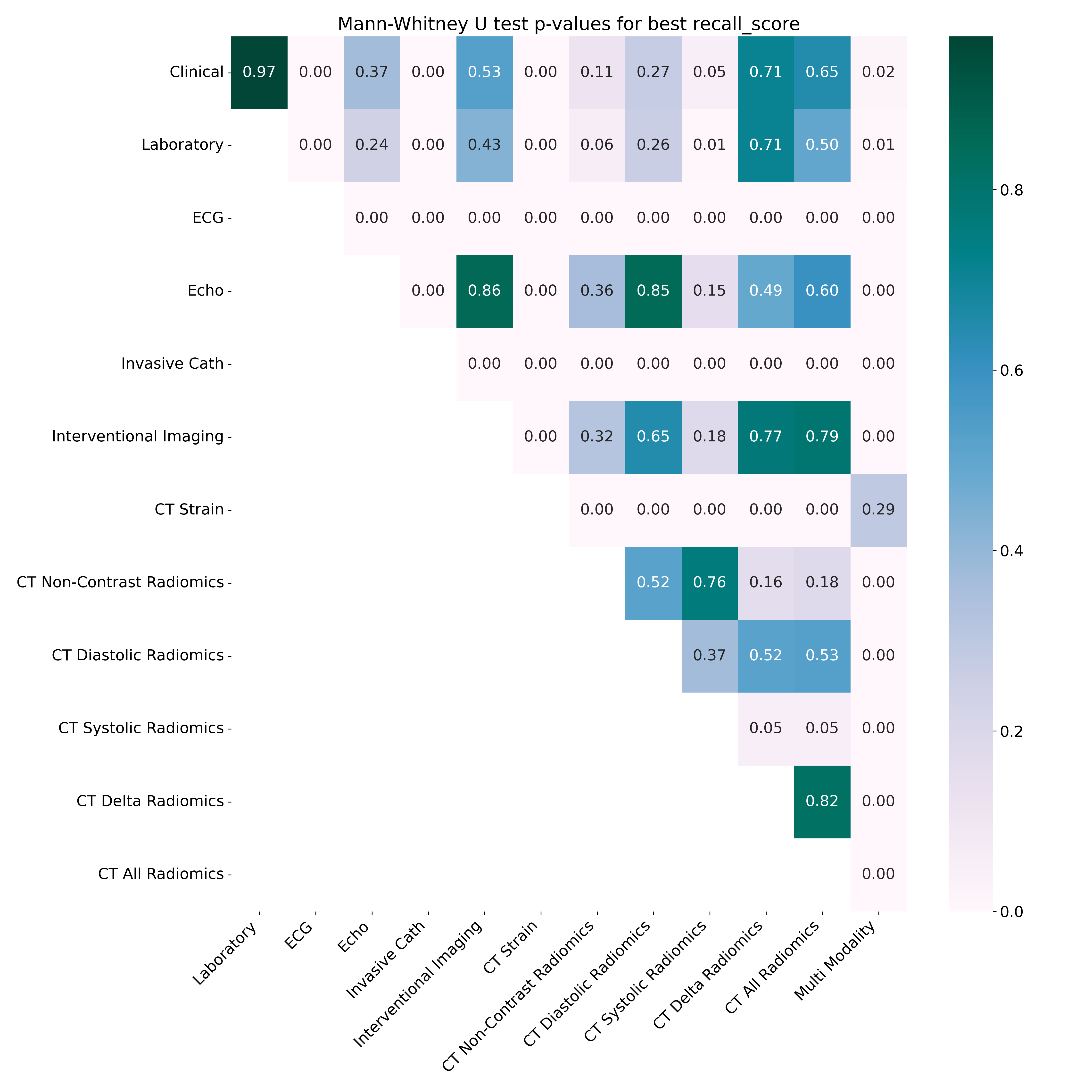
 **Supplemental Figure 3:** Heat map visualizing the Mann-Whitney U-test results for comparing the sensitivity of different modalities.


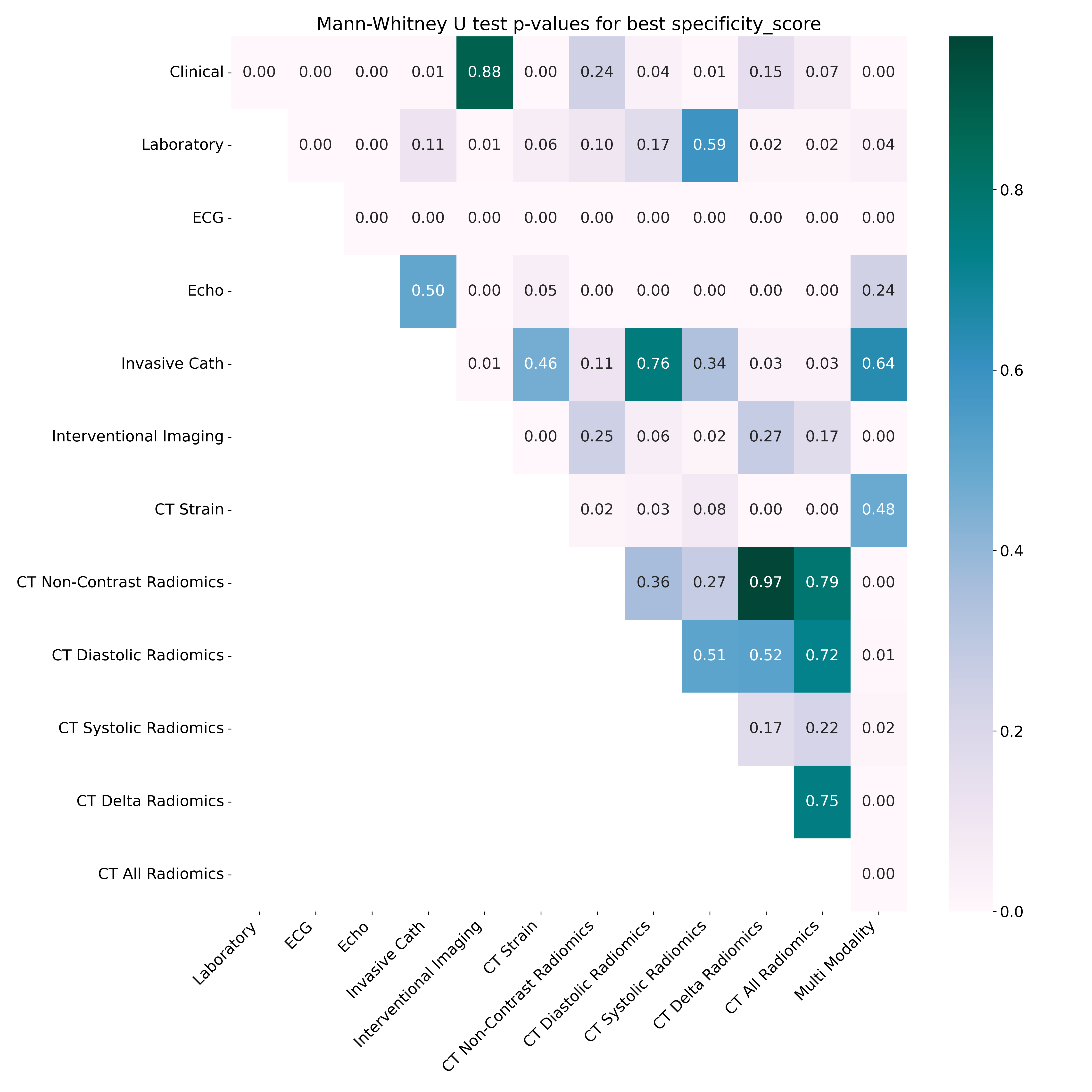
 **Supplemental Figure 4:** Heat map visualizing the Mann-Whitney U-test results for comparing the specificity of different modalities.


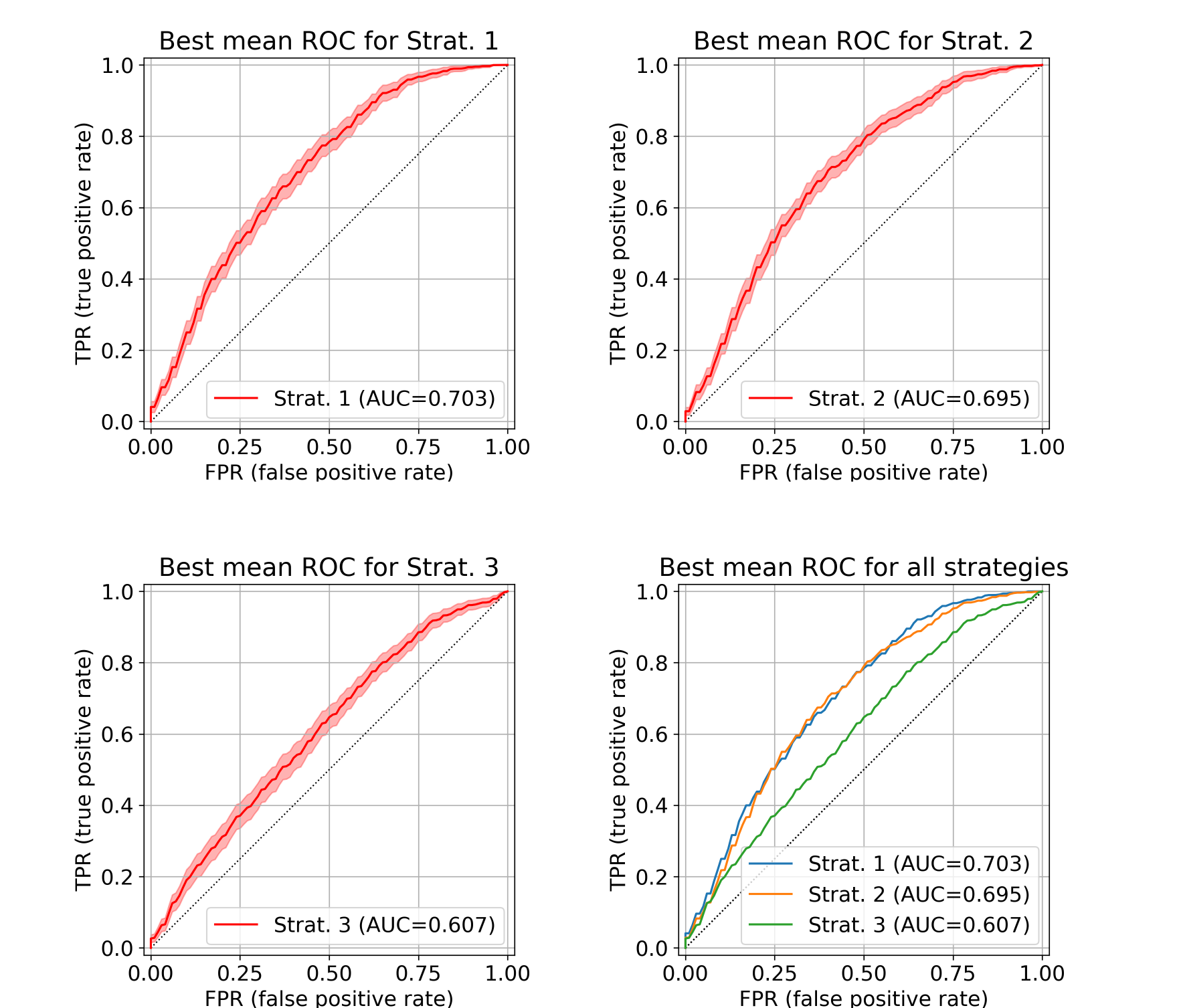


**Supplemental Figure 5:** ROC curve of top models in clinical data. Strat. 1 (RFE+LR), Strat. 2 (UniVa+LR), Strat. 3 (MRMR+LR)


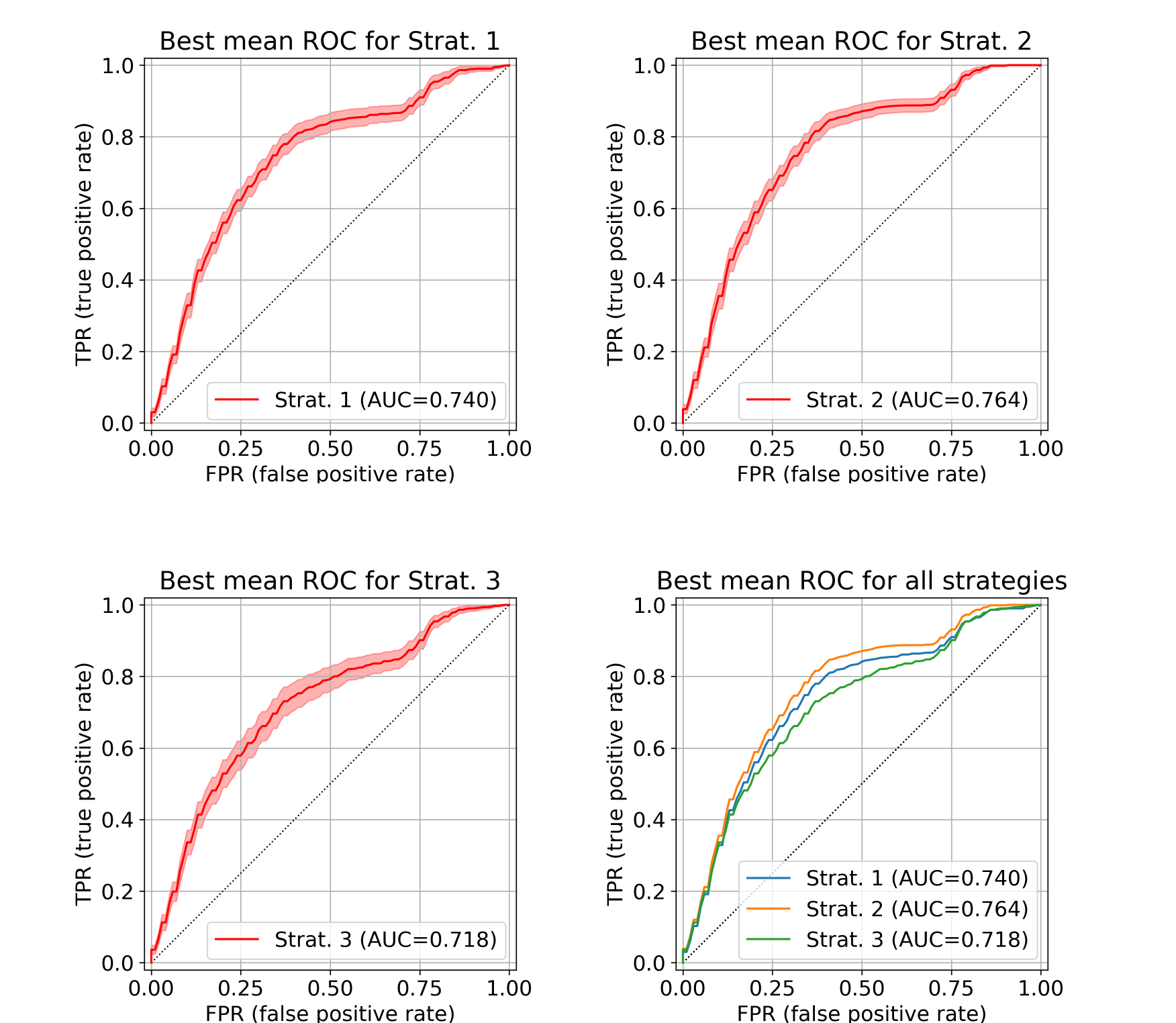


**Supplemental Figure 6:** ROC curve of top models in laboratory data. Strat. 1 (RFE+LR), Strat. 2 (UniVa+LR)


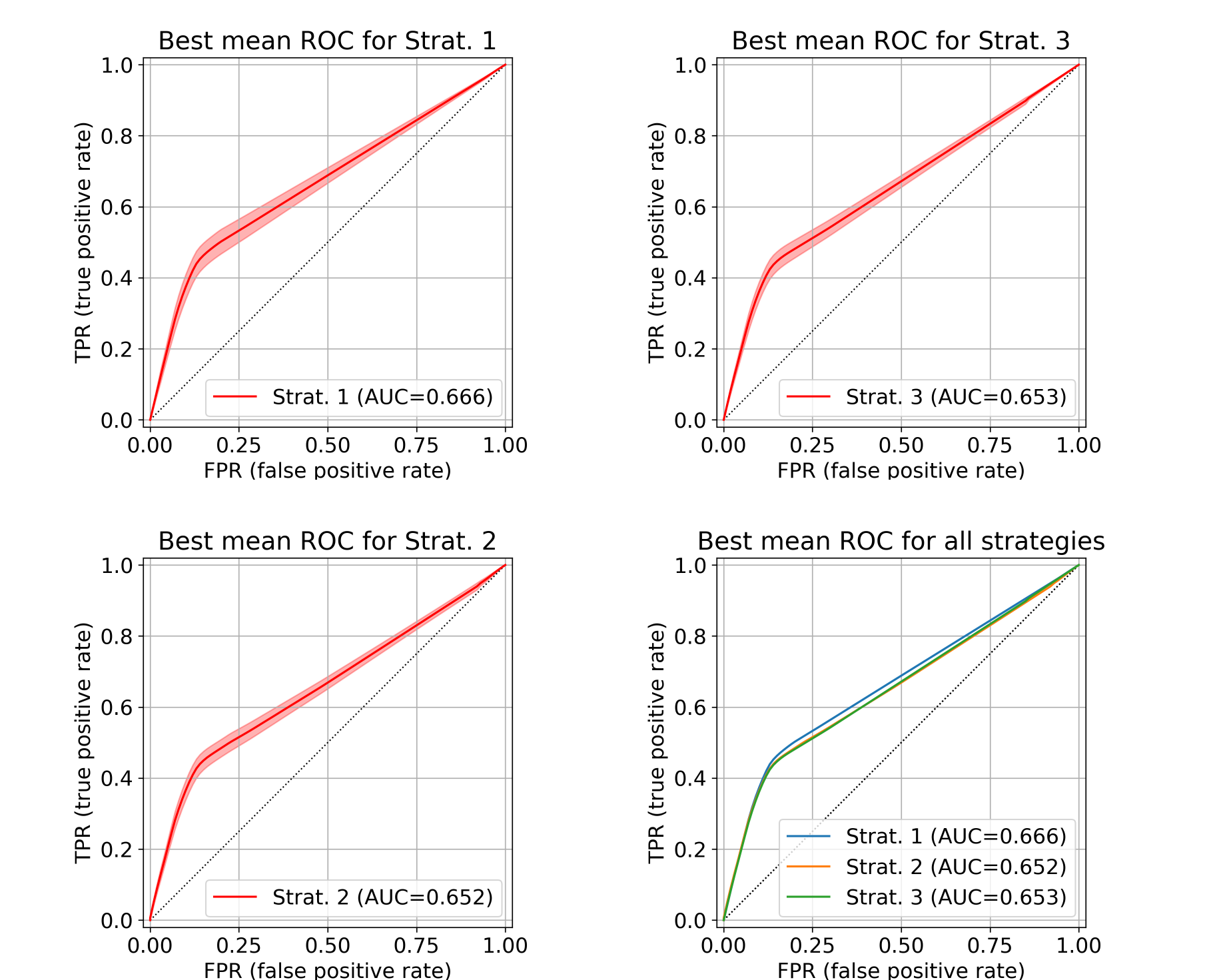


**Supplemental Figure 7:** ROC curve of top models in ECG data. Strat. 1 (RFE+AdaBo), Strat. 2 (UniVa+ AdaBo), Strat. 3 (MRMR+ AdaBo)


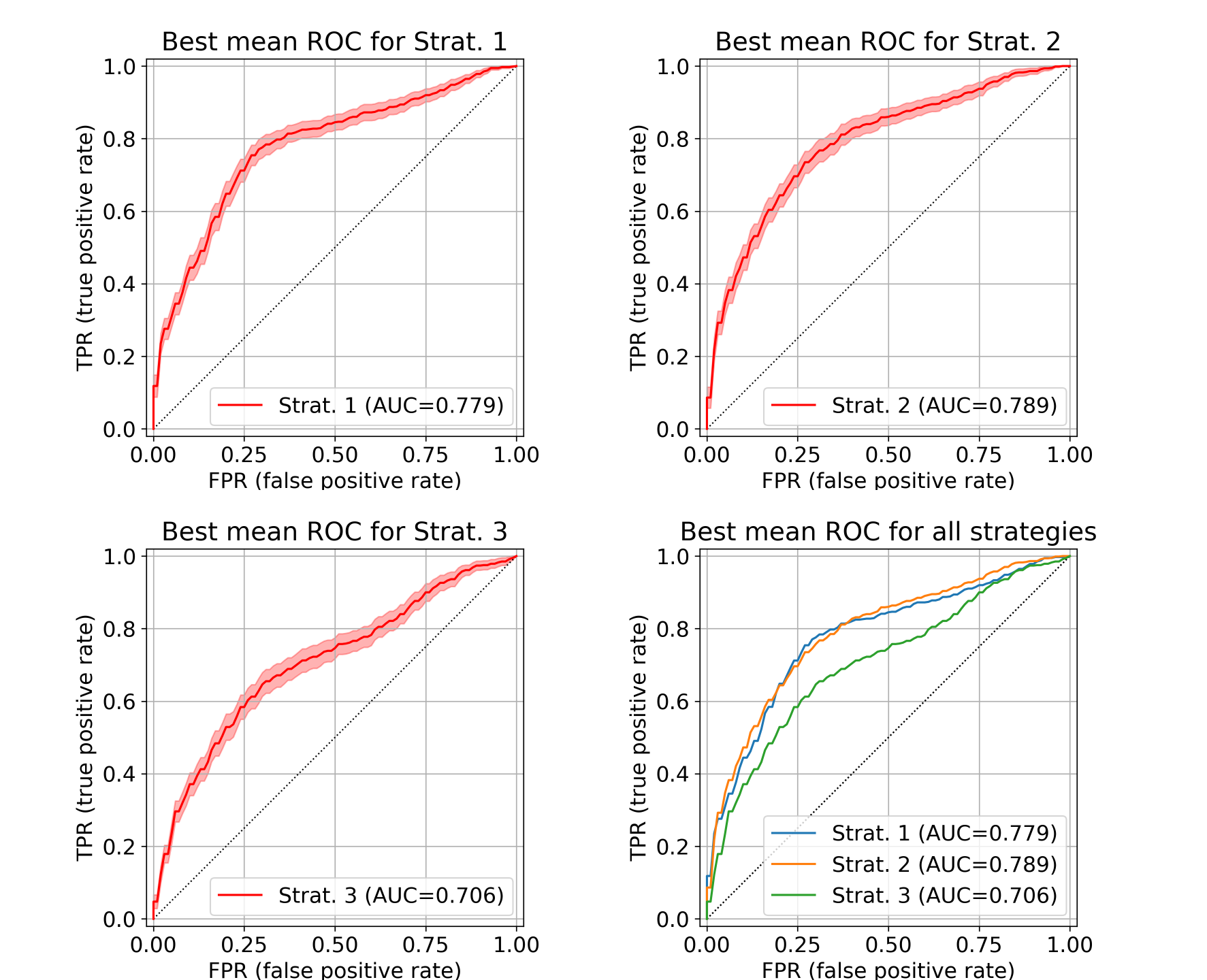


**Supplemental Figure 8:** ROC curve of top models in Echo data. Strat. 1 (RFE+LR), Strat. 2 (UniVa+SVM), Strat. 3 (MRMR+LR)


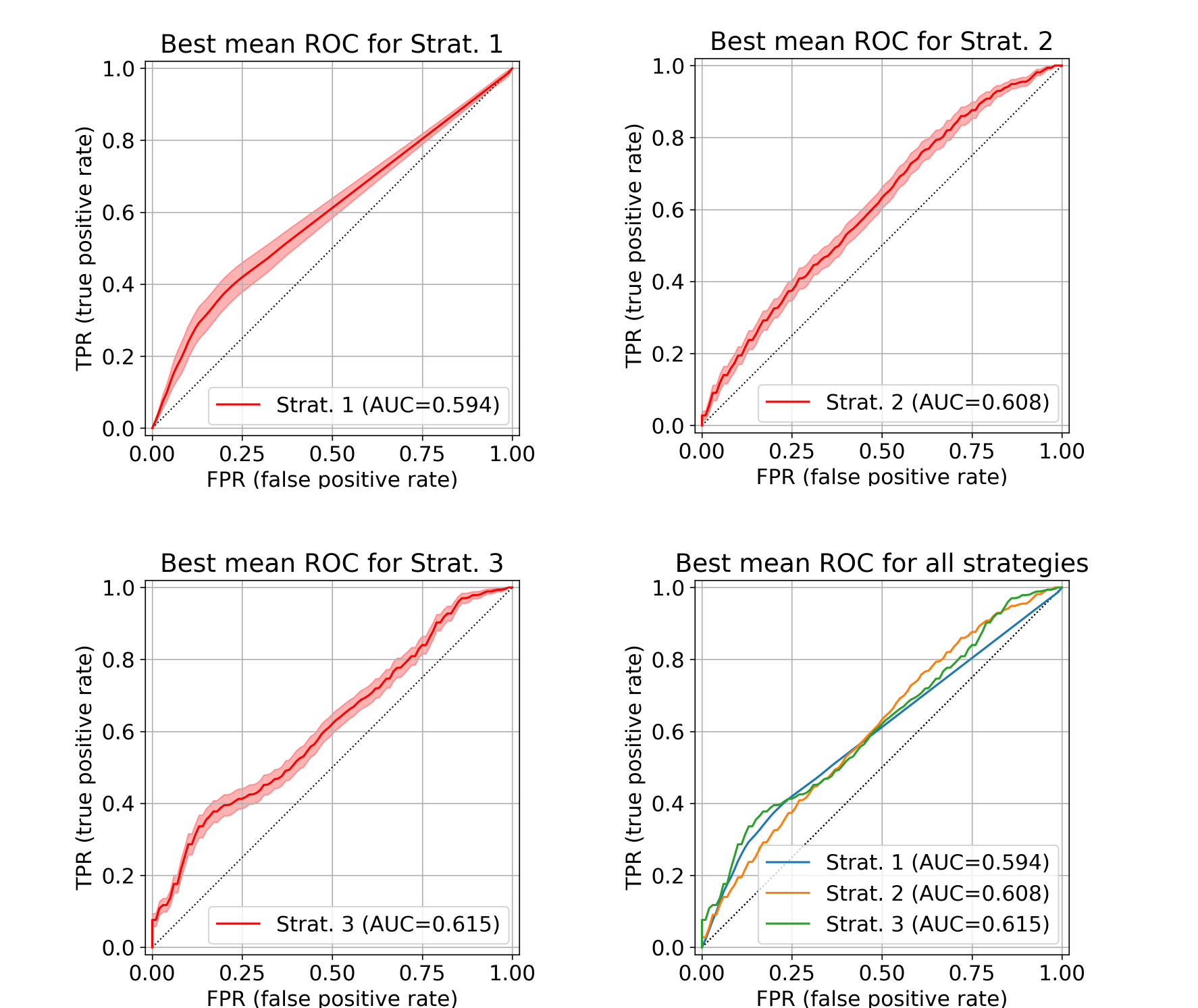


**Supplemental Figure 9:** ROC curve of top models in invasive cath data. Strat. 1 (RFE+AdaBo), Strat. 2 (UniVa+LR), Strat. 3 (MRMR+LR)


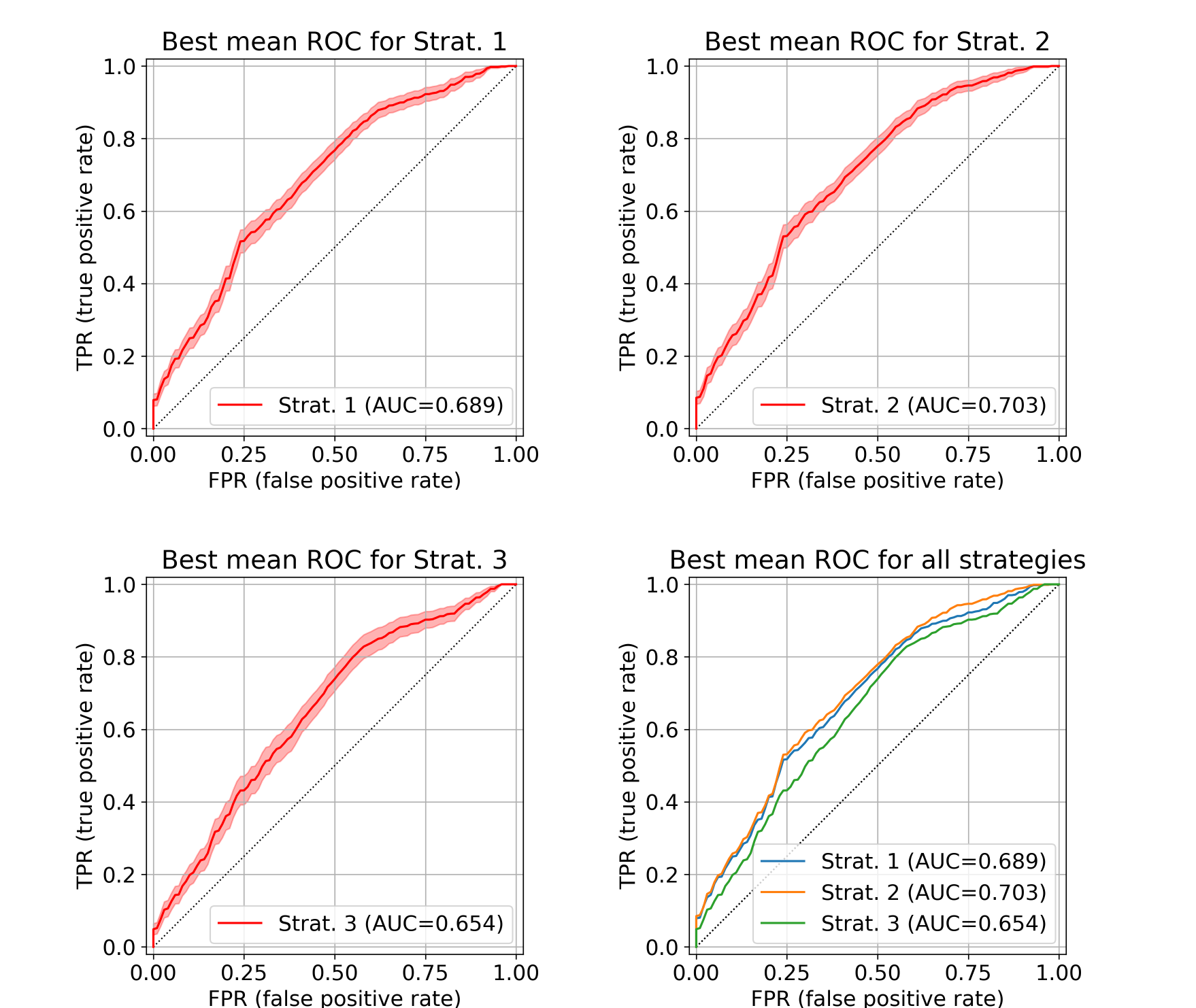


**Supplemental Figure 10:** ROC curve of top models in interventional imaging data. Strat. 1 (RFE+LR), Strat. 2 (UniVa+LR), Strat. 3 (MRMR+LR)


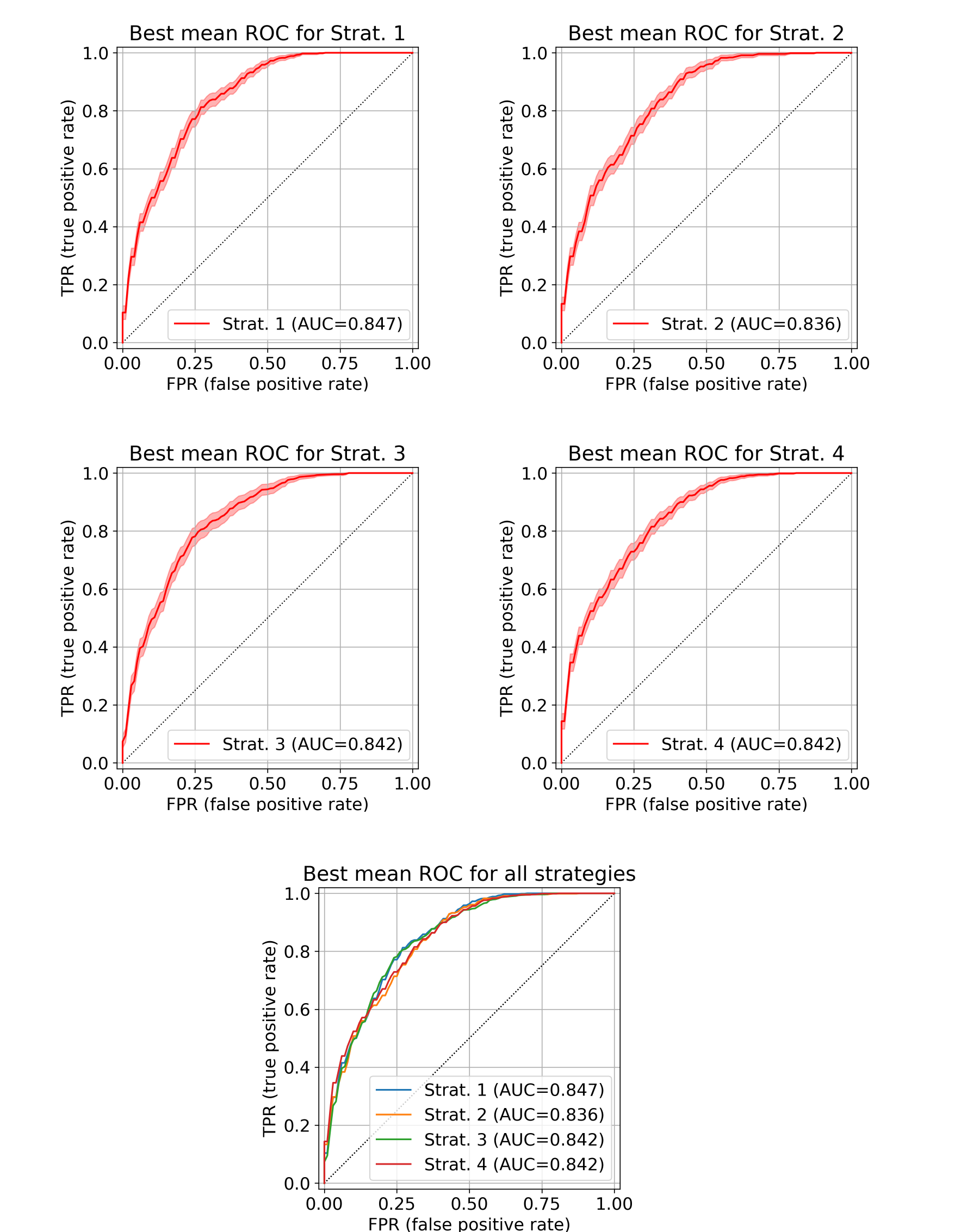


**Supplemental Figure 11:** ROC curve of top models in CT strain data. Strat. 1 (Manual+LR), Strat. 2 (RFE+LR), Strat. 3 (UniVa+AdaBo) Strat. 4 (MRMR+SVM


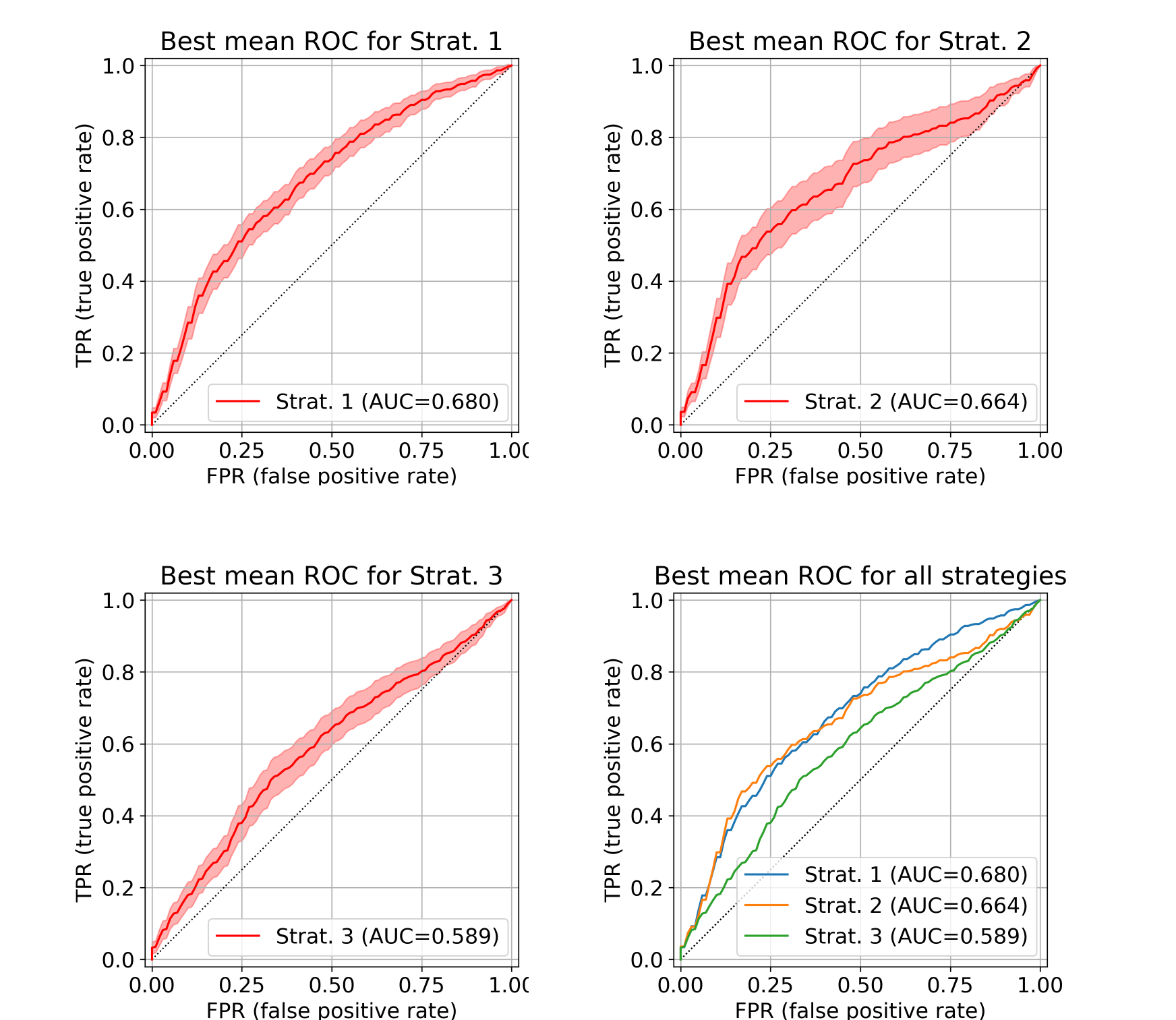


**Supplemental Figure 12:** ROC curve of top models in non-contrast CT Radiomics data. Strat. 1 (RFE+LR), Strat. 2 (UniVa+LR), Strat. 3 (MRMR+LR)


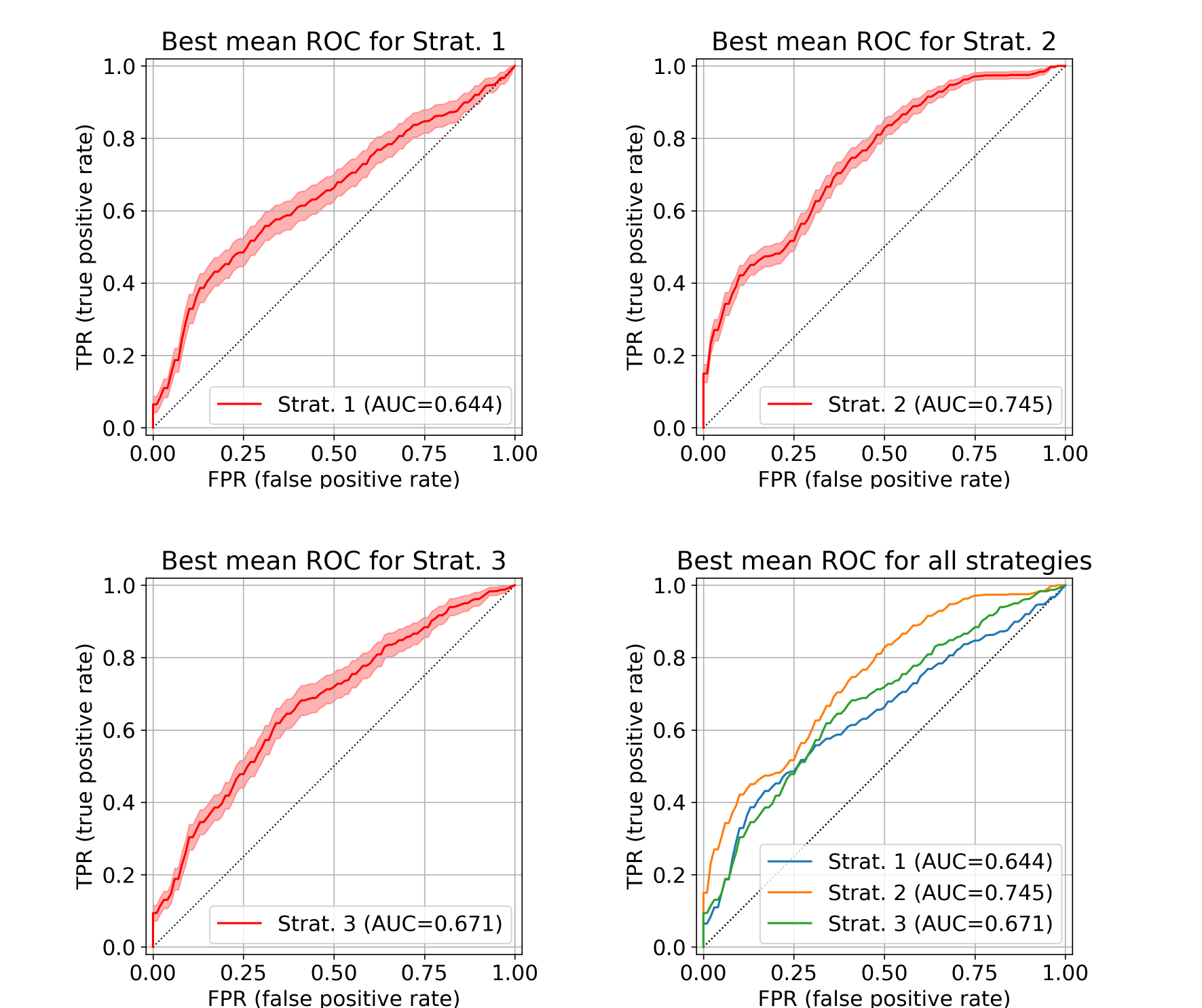


**Supplemental Figure 13:** ROC curve of top models in diastolic CT Radiomics data. Strat. 1 (RFE+LR), Strat. 2 (UniVa+LR), Strat. 3 (MRMR+LR)


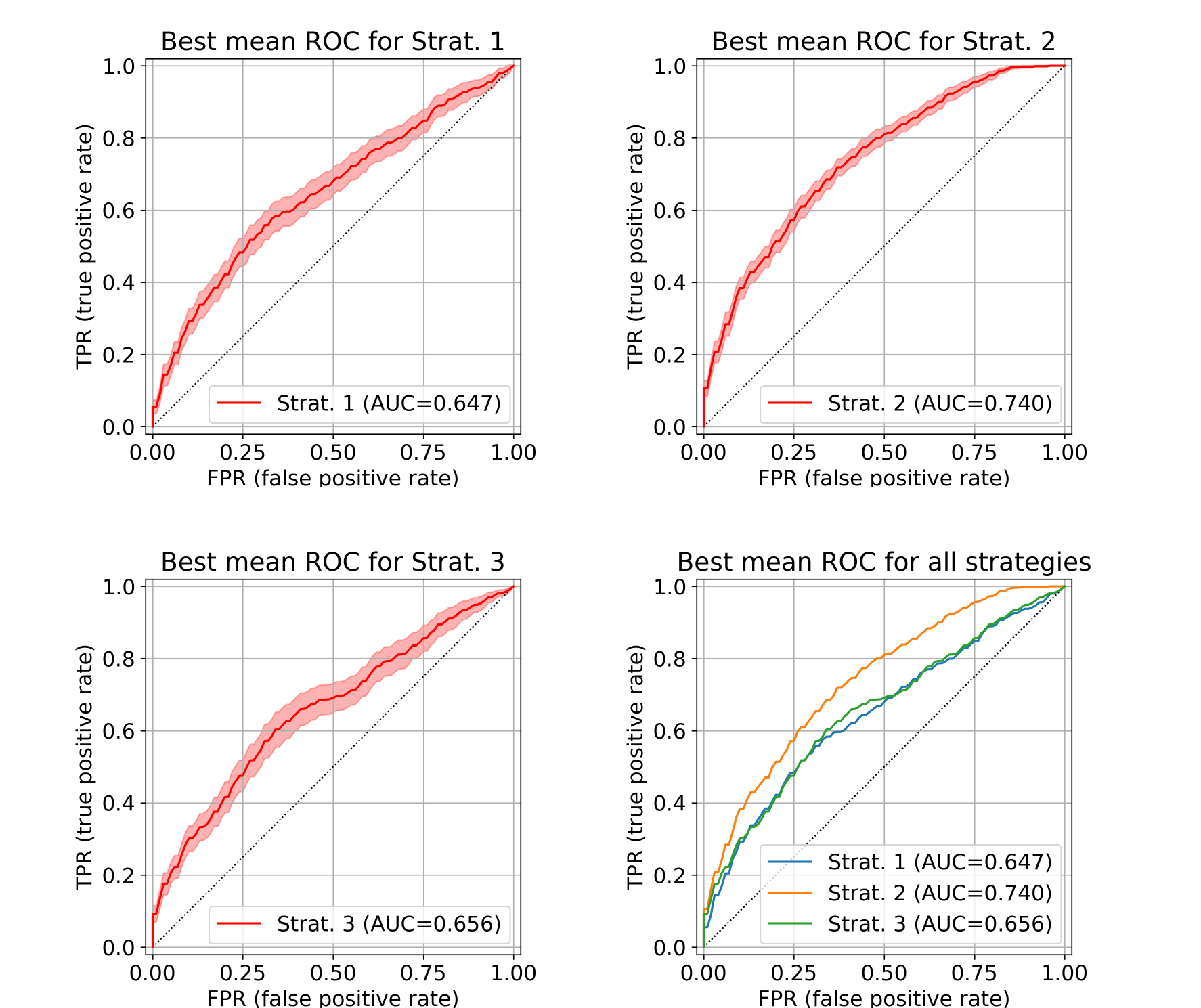


**Supplemental Figure 14:** ROC curve of top models in systolic CT Radiomics data. Strat. 1 (RFE+LR), Strat. 2 (UniVa+LR), Strat. 3 (MRMR+LR)


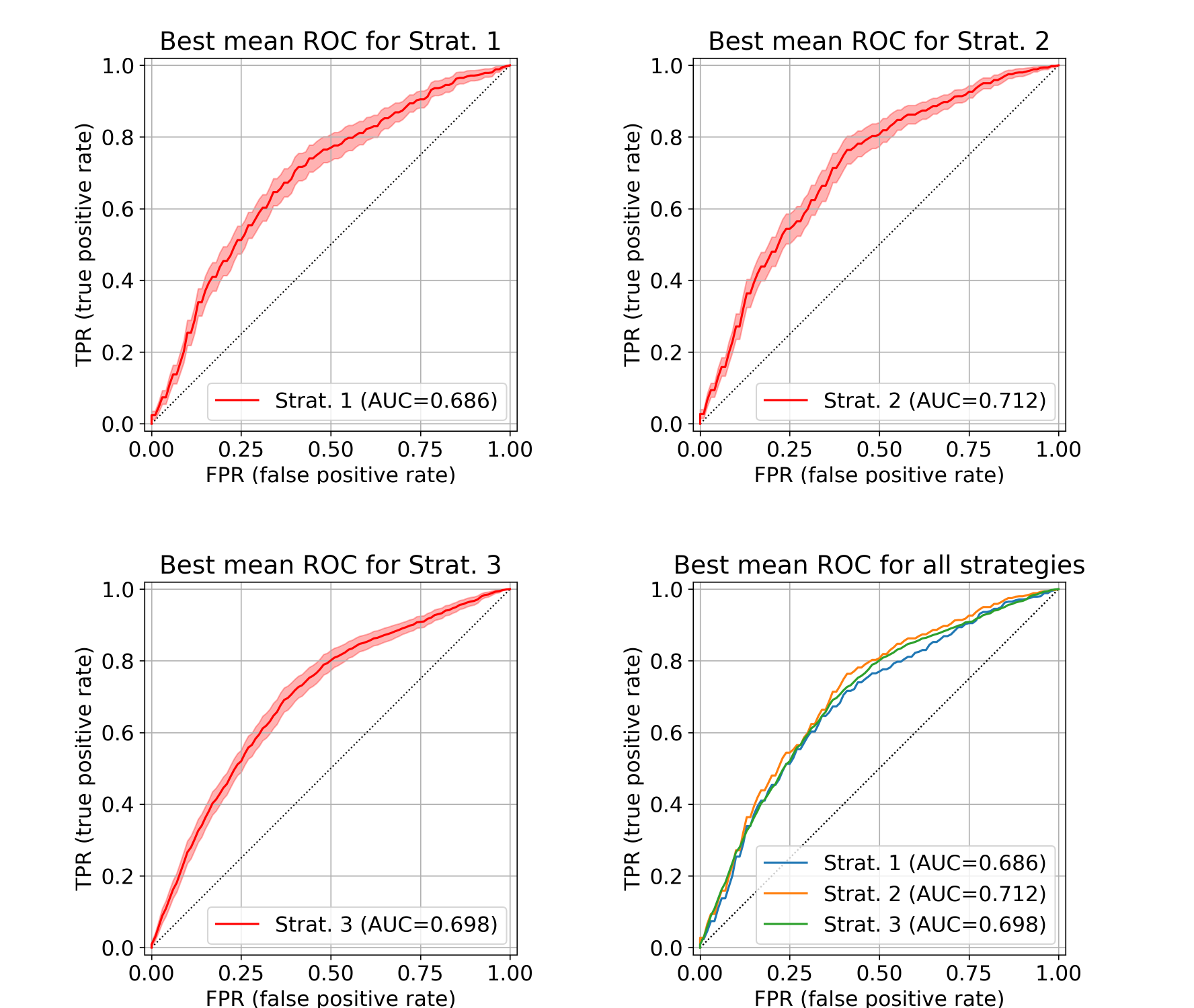


**Supplemental Figure 15:** ROC curve of top models in delta CT Radiomics data. Strat. 1 (RFE+LR), Strat. 2 (UniVa+LR), Strat. 3 (MRMR+AdaBo)


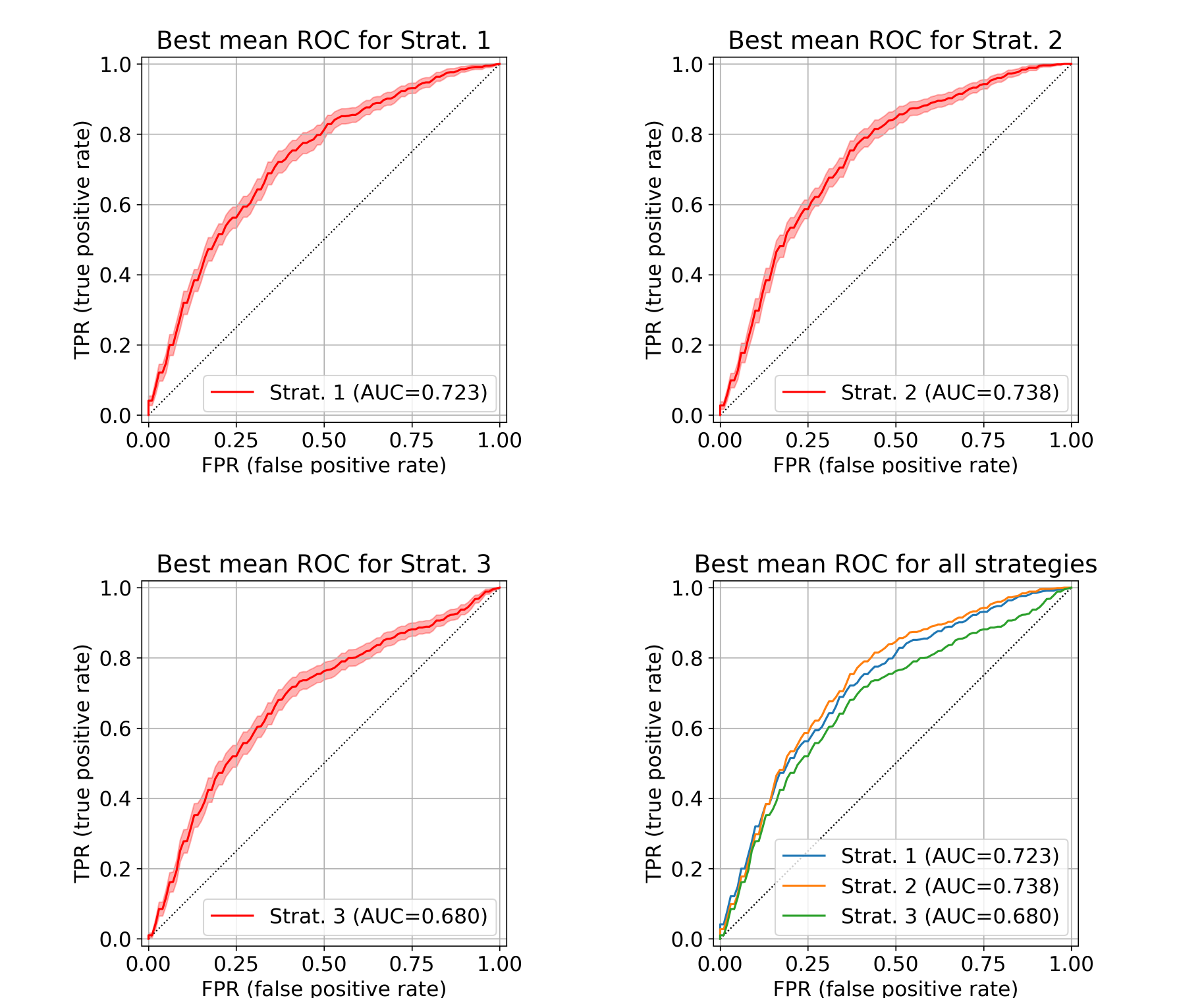


**Supplemental Figure 16:** ROC curve of top models in all CT Radiomics data. Strat. 1 (RFE+LR), Strat. 2 (UniVa+LR), Strat. 3 (MRMR+LR)


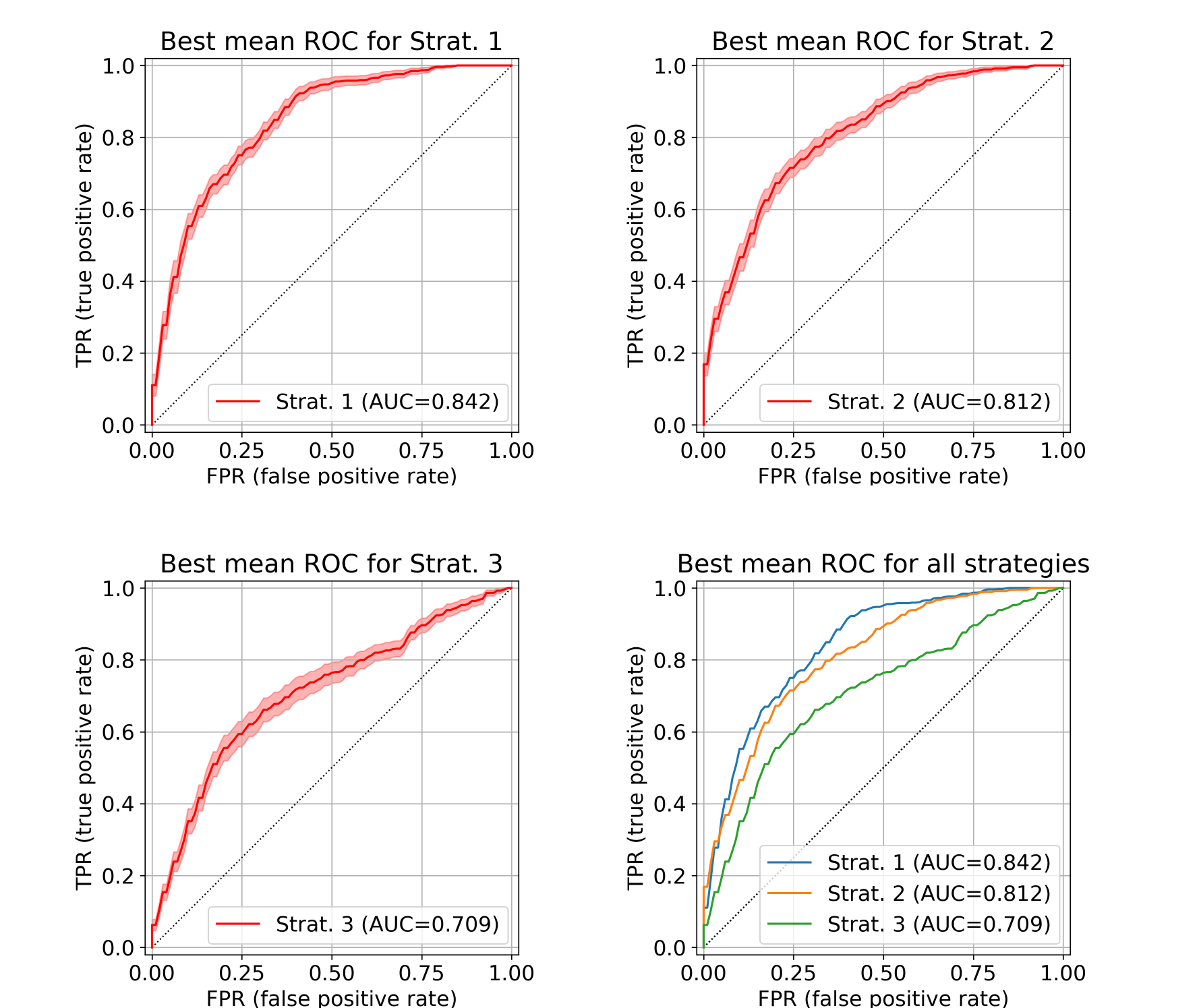


**Supplemental Figure 17:** ROC curve of top models in mulit-modality data. Strat. 1 (RFE+LR), Strat. 2 (UniVa+LR), Strat. 3 (MRMR+LR)


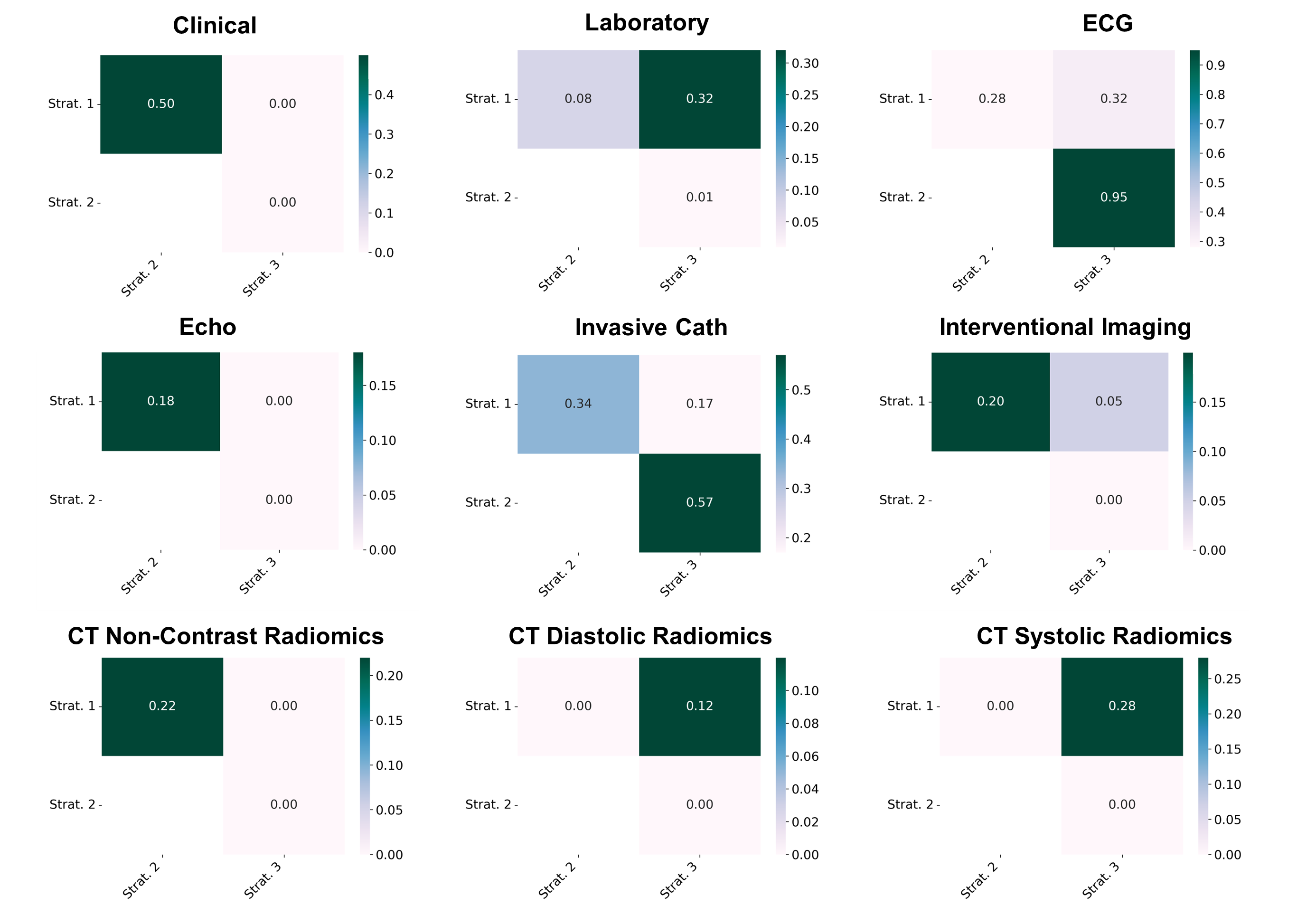


**Supplemental Figure 18:** Heat map visualizing the Mann-Whitney U-test results for comparing AUC across different models in various modalities. Clinical: Strat. 1 (RFE+LR), Strat. 2 (UniVa+LR), Strat. 3 (MRMR+LR); Laboratory: Strat. 1 (RFE+LR), Strat. 2 (UniVa+LR), Strat. 3 (MRMR+LR); ECG: Strat. 1 (RFE+AdaBo), Strat. 2 (UniVa+ AdaBo), Strat. 3 (MRMR+ AdaBo); Echo: Strat. 1 (RFE+LR), Strat. 2 (UniVa+SVM), Strat. 3 (MRMR+LR); Invasive Cath: Strat. 1 (RFE+AdaBo), Strat. 2 (UniVa+LR), Strat. 3 (MRMR+LR); Interventional Imaging: Strat. 1 (RFE+LR), Strat. 2 (UniVa+LR), Strat. 3 (MRMR+LR); CT Non-Contrast Radiomics: Strat. 1 (RFE+LR), Strat. 2 (UniVa+LR), Strat. 3 (MRMR+LR); CT Diastolic Radiomics: Strat. 1 (RFE+LR), Strat. 2 (UniVa+LR), Strat. 3 (MRMR+LR); CT Systolic Radiomics: Strat. 1 (RFE+LR), Strat. 2 (UniVa+LR), Strat. 3 (MRMR+LR).


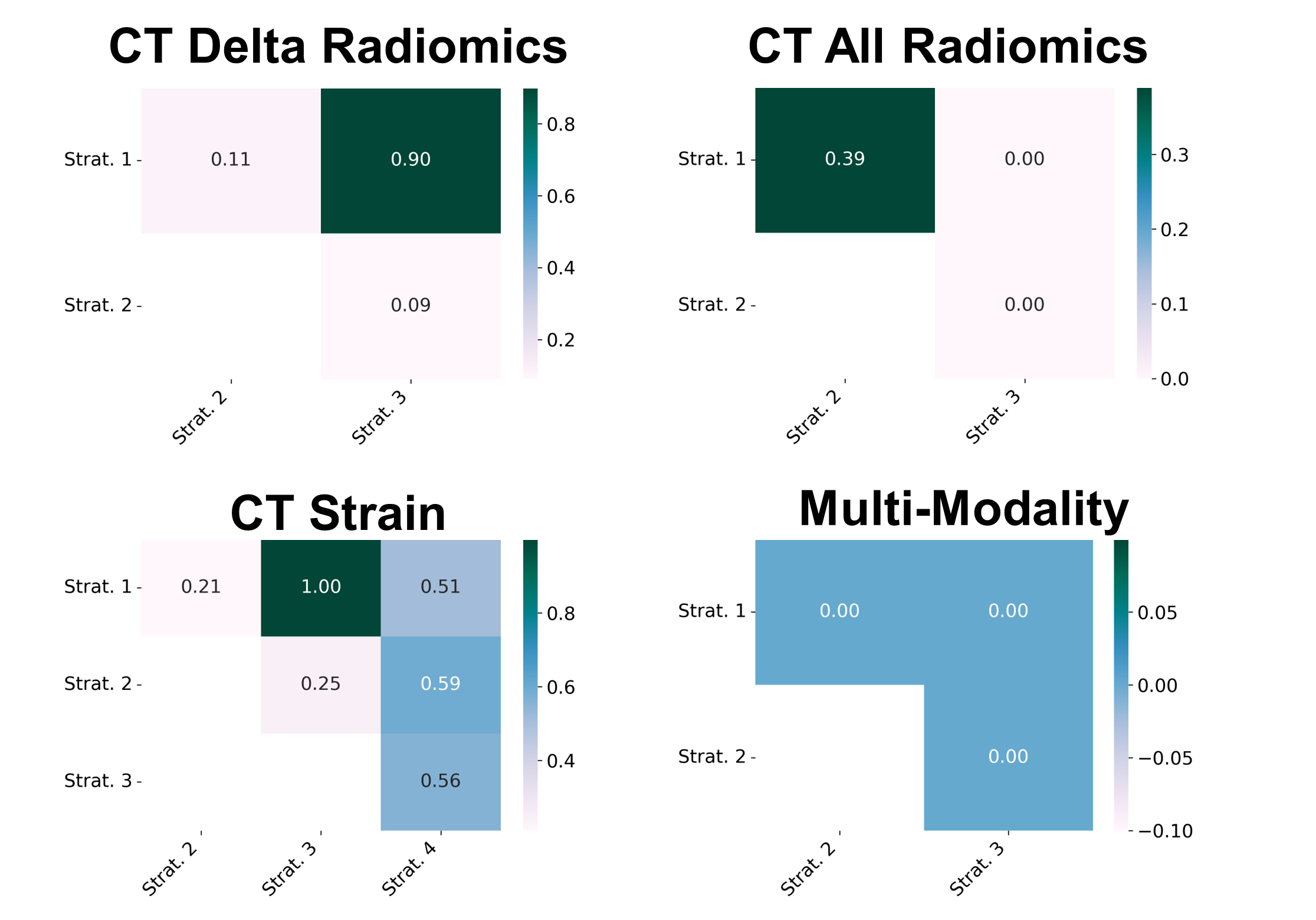


**Supplemental Figure 19:** Heat map visualizing the Mann-Whitney U-test results for comparing AUC across different models in various modalities. CT Delta Radiomics: Strat. 1 (RFE+LR), Strat. 2 (UniVa+LR), Strat. 3 (MRMR+AdaBo); CT All Radiomics: Strat. 1 (RFE+LR), Strat. 2 (UniVa+LR), Strat. 3 (MRMR+LR); CT Strain: Strat. 1 (Manual+LR), Strat. 2 (RFE+LR), Strat. 3 (UniVa+AdaBo) Strat. 4 (MRMR+SVM); Multi-Modality: Strat. 1 (RFE+LR), Strat. 2 (UniVa+LR), Strat. 3 (MRMR+LR)


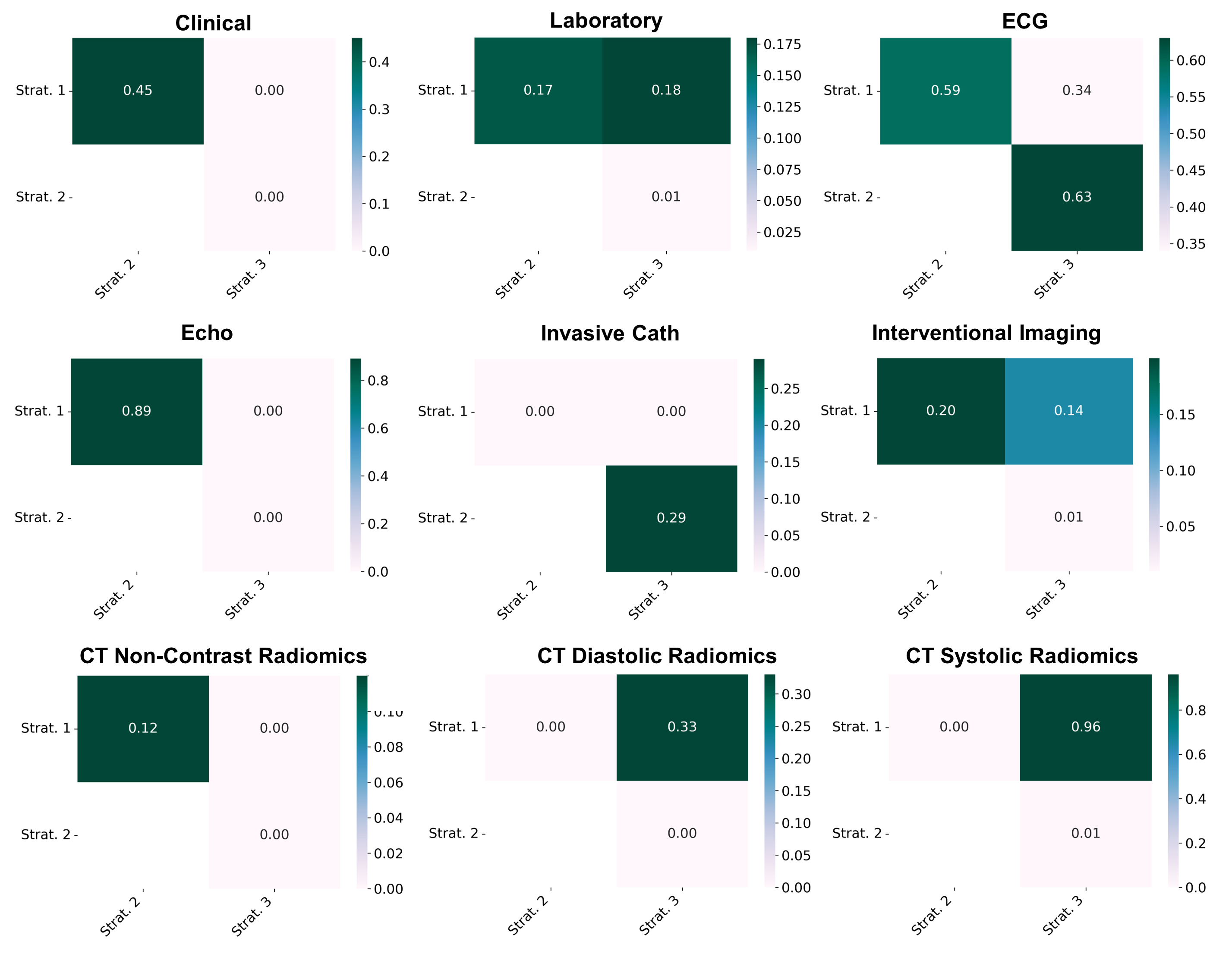


**Supplemental Figure 20:** Heat map visualizing the Mann-Whitney U-test results for comparing accuracy across different models in various modalities. Clinical: Strat. 1 (RFE+LR), Strat. 2 (UniVa+LR), Strat. 3 (MRMR+LR); Laboratory: Strat. 1 (RFE+LR), Strat. 2 (UniVa+LR), Strat. 3 (MRMR+LR); ECG: Strat. 1 (RFE+AdaBo), Strat. 2 (UniVa+ AdaBo), Strat. 3 (MRMR+ AdaBo); Echo: Strat. 1 (RFE+LR), Strat. 2 (UniVa+SVM), Strat. 3 (MRMR+LR); Invasive Cath: Strat. 1 (RFE+AdaBo), Strat. 2 (UniVa+LR), Strat. 3 (MRMR+LR); Interventional Imaging: Strat. 1 (RFE+LR), Strat. 2 (UniVa+LR), Strat. 3 (MRMR+LR); CT Non-Contrast Radiomics: Strat. 1 (RFE+LR), Strat. 2 (UniVa+LR), Strat. 3 (MRMR+LR); CT Diastolic Radiomics: Strat. 1 (RFE+LR), Strat. 2 (UniVa+LR), Strat. 3 (MRMR+LR); CT Systolic Radiomics: Strat. 1 (RFE+LR), Strat. 2 (UniVa+LR), Strat. 3 (MRMR+LR).


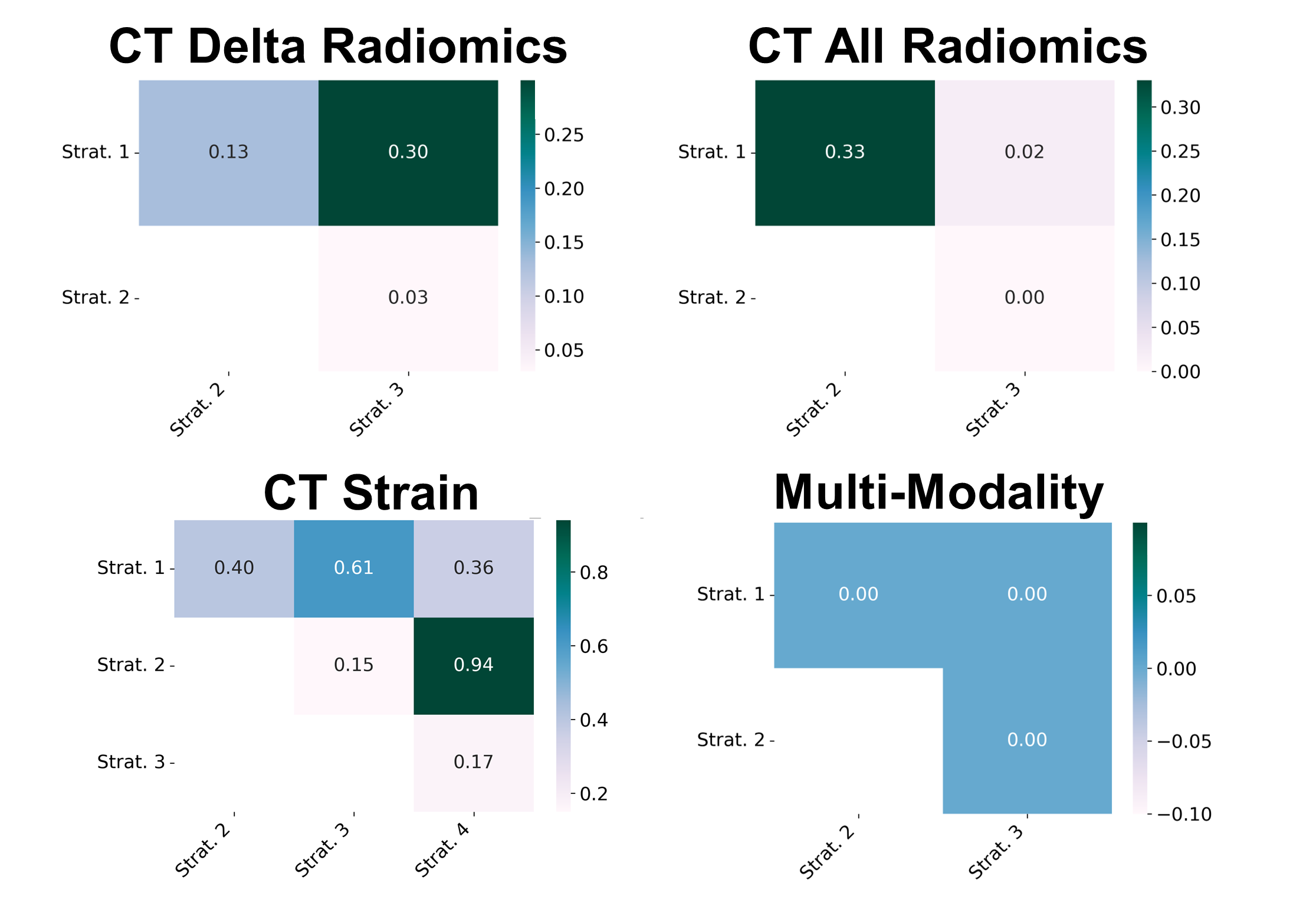


**Supplemental Figure 21:** Heat map visualizing the Mann-Whitney U-test results for comparing accuracy across different models in various modalities. CT Delta Radiomics: Strat. 1 (RFE+LR), Strat. 2 (UniVa+LR), Strat. 3 (MRMR+AdaBo); CT All Radiomics: Strat. 1 (RFE+LR), Strat. 2 (UniVa+LR), Strat. 3 (MRMR+LR); CT Strain: Strat. 1 (Manual+LR), Strat. 2 (RFE+LR), Strat. 3 (UniVa+AdaBo) Strat. 4 (MRMR+SVM); Multi-Modality: Strat. 1 (RFE+LR), Strat. 2 (UniVa+LR), Strat. 3 (MRMR+LR)


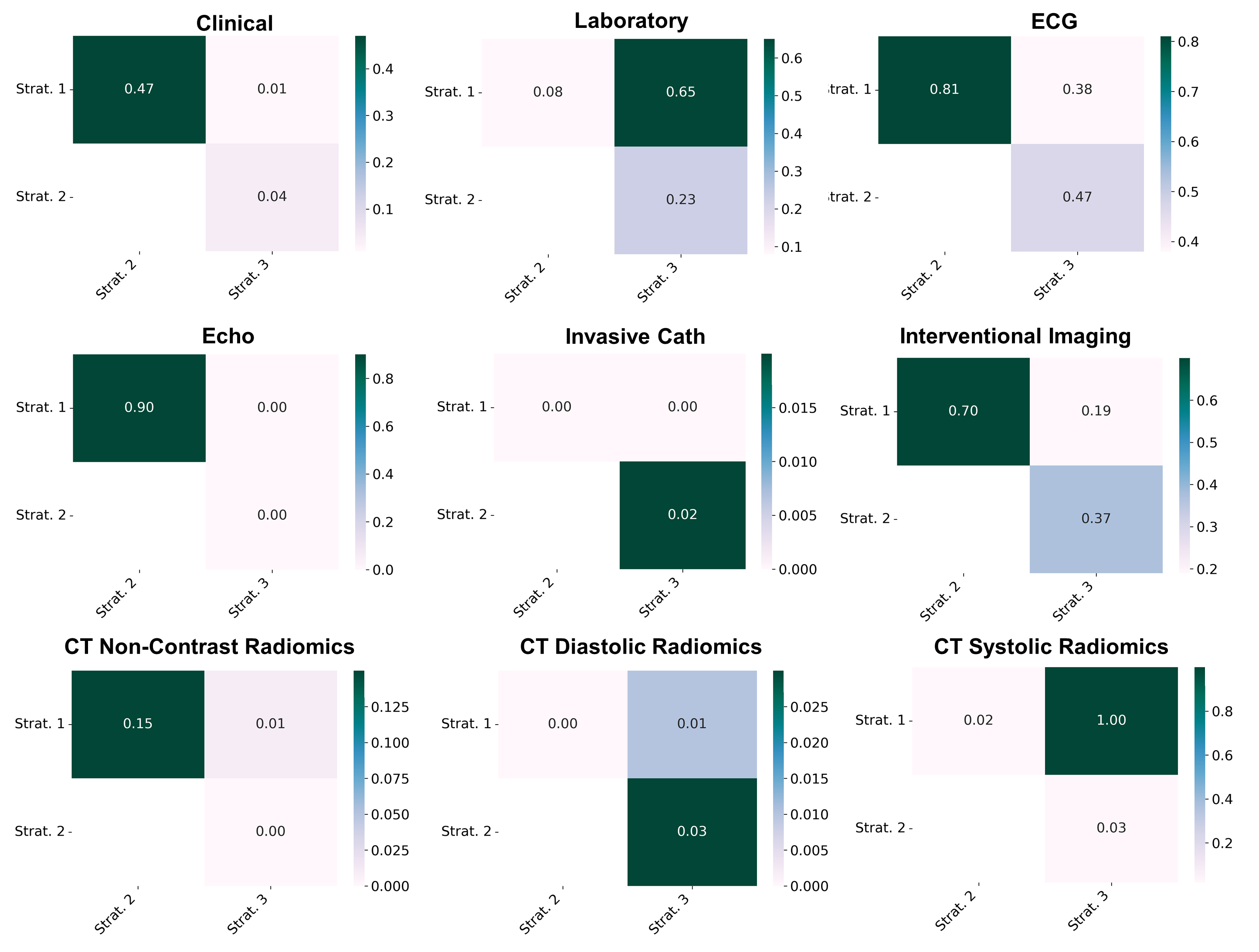


**Supplemental Figure 22:** Heat map visualizing the Mann-Whitney U-test results for comparing sensitivity across different models in various modalities. Clinical: Strat. 1 (RFE+LR), Strat. 2 (UniVa+LR), Strat. 3 (MRMR+LR); Laboratory: Strat. 1 (RFE+LR), Strat. 2 (UniVa+LR), Strat. 3 (MRMR+LR); ECG: Strat. 1 (RFE+AdaBo), Strat. 2 (UniVa+ AdaBo), Strat. 3 (MRMR+ AdaBo); Echo: Strat. 1 (RFE+LR), Strat. 2 (UniVa+SVM), Strat. 3 (MRMR+LR); Invasive Cath: Strat. 1 (RFE+AdaBo), Strat. 2 (UniVa+LR), Strat. 3 (MRMR+LR); Interventional Imaging: Strat. 1 (RFE+LR), Strat. 2 (UniVa+LR), Strat. 3 (MRMR+LR); CT Non-Contrast Radiomics: Strat. 1 (RFE+LR), Strat. 2 (UniVa+LR), Strat. 3 (MRMR+LR); CT Diastolic Radiomics: Strat. 1 (RFE+LR), Strat. 2 (UniVa+LR), Strat. 3 (MRMR+LR); CT Systolic Radiomics: Strat. 1 (RFE+LR), Strat. 2 (UniVa+LR), Strat. 3 (MRMR+LR).


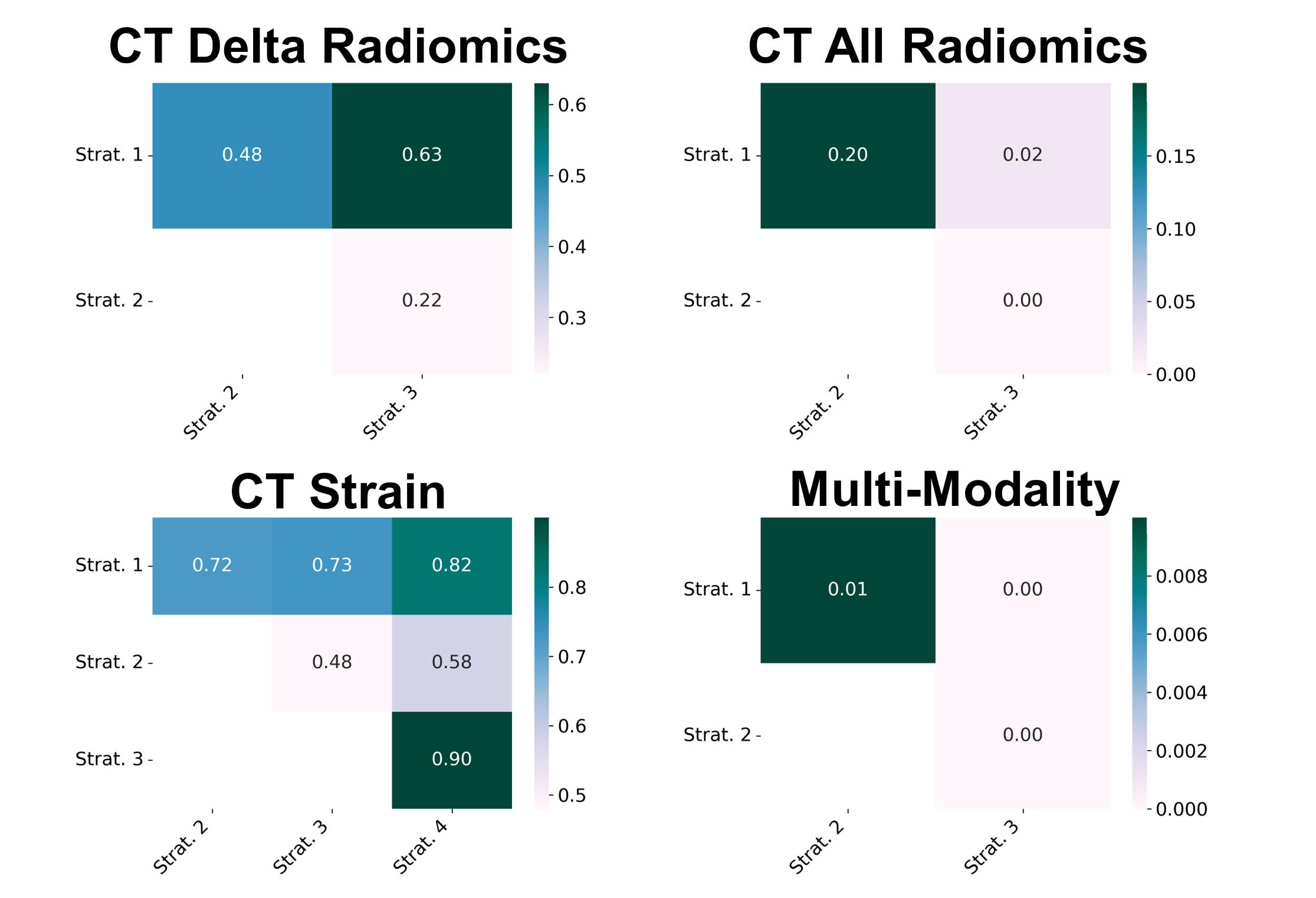


**Supplemental Figure 23:** Heat map visualizing the Mann-Whitney U-test results for comparing sensitivity across different models in various modalities. CT Delta Radiomics: Strat. 1 (RFE+LR), Strat. 2 (UniVa+LR), Strat. 3 (MRMR+AdaBo); CT All Radiomics: Strat. 1 (RFE+LR), Strat. 2 (UniVa+LR), Strat. 3 (MRMR+LR); CT Strain: Strat. 1 (Manual+LR), Strat. 2 (RFE+LR), Strat. 3 (UniVa+AdaBo) Strat. 4 (MRMR+SVM); Multi-Modality: Strat. 1 (RFE+LR), Strat. 2 (UniVa+LR), Strat. 3 (MRMR+LR)


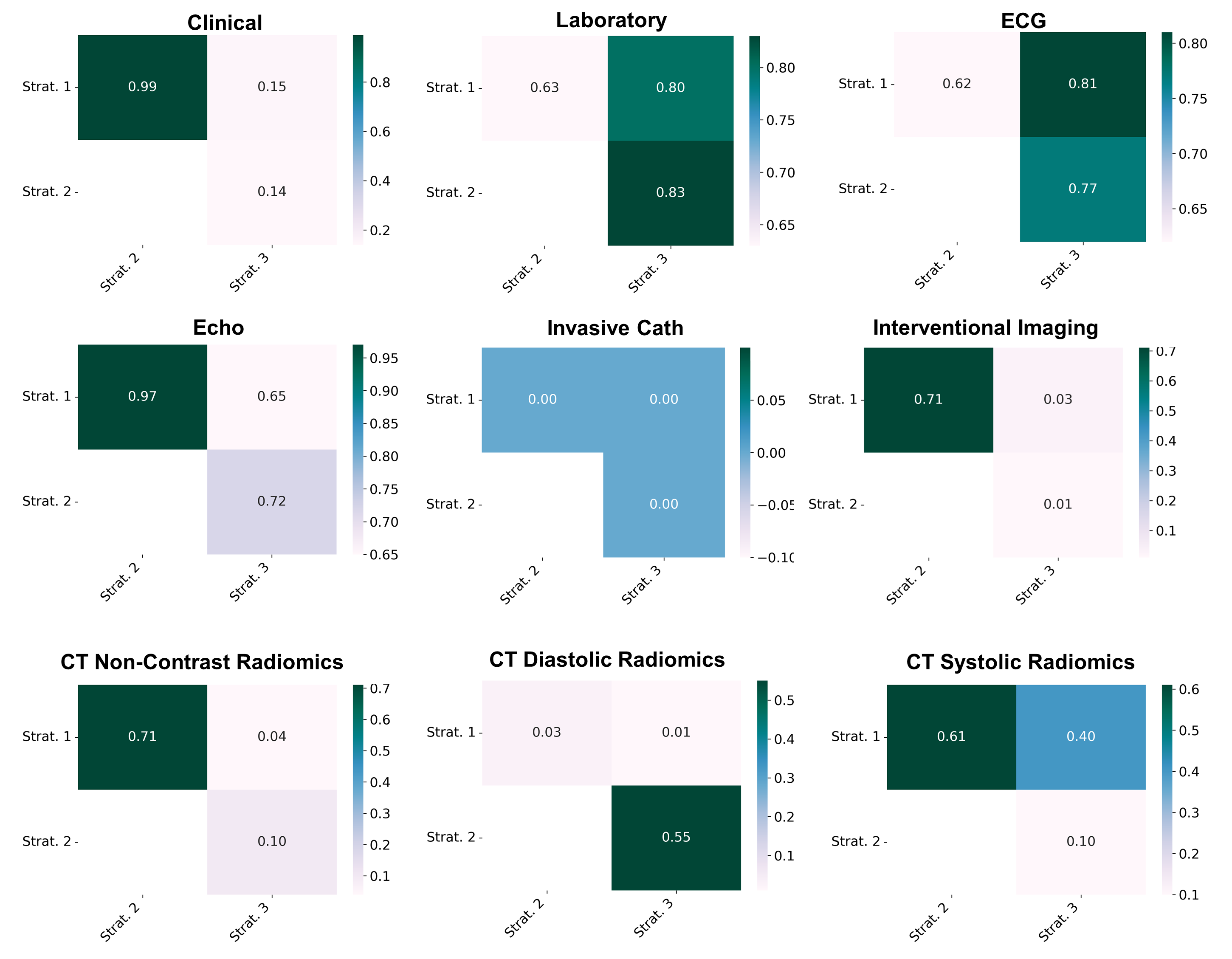


**Supplemental Figure 24:** Heat map visualizing the Mann-Whitney U-test results for comparing specificity across different models in various modalities. Clinical: Strat. 1 (RFE+LR), Strat. 2 (UniVa+LR), Strat. 3 (MRMR+LR); Laboratory: Strat. 1 (RFE+LR), Strat. 2 (UniVa+LR), Strat. 3 (MRMR+LR); ECG: Strat. 1 (RFE+AdaBo), Strat. 2 (UniVa+ AdaBo), Strat. 3 (MRMR+ AdaBo); Echo: Strat. 1 (RFE+LR), Strat. 2 (UniVa+SVM), Strat. 3 (MRMR+LR); Invasive Cath: Strat. 1 (RFE+AdaBo), Strat. 2 (UniVa+LR), Strat. 3 (MRMR+LR); Interventional Imaging: Strat. 1 (RFE+LR), Strat. 2 (UniVa+LR), Strat. 3 (MRMR+LR); CT Non-Contrast Radiomics: Strat. 1 (RFE+LR), Strat. 2 (UniVa+LR), Strat. 3 (MRMR+LR); CT Diastolic Radiomics: Strat. 1 (RFE+LR), Strat. 2 (UniVa+LR), Strat. 3 (MRMR+LR); CT Systolic Radiomics: Strat. 1 (RFE+LR), Strat. 2 (UniVa+LR), Strat. 3 (MRMR+LR).


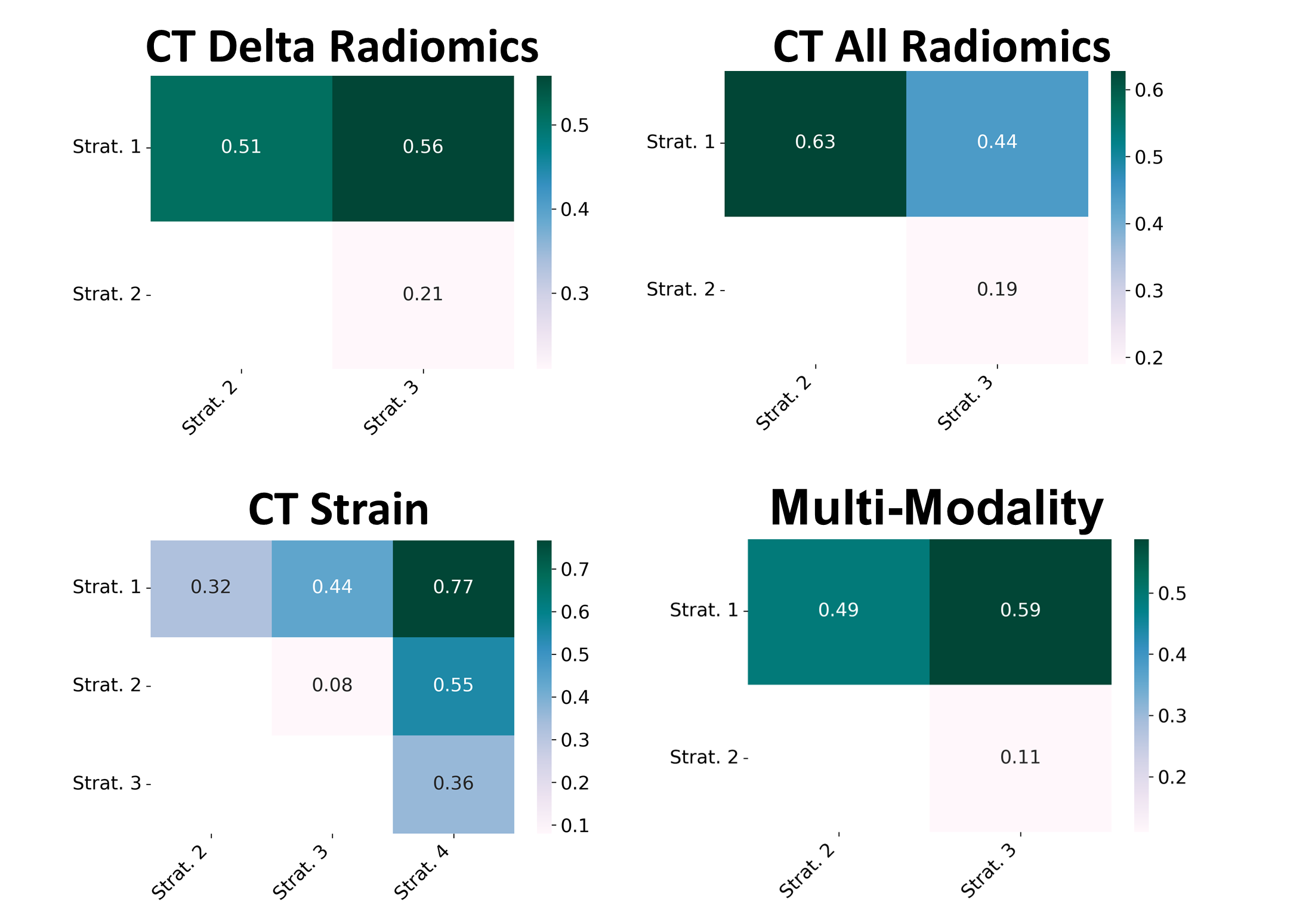


**Supplemental Figure 25:** Heat map visualizing the Mann-Whitney U-test results for comparing specificity across different models in various modalities. CT Delta Radiomics: Strat. 1 (RFE+LR), Strat. 2 (UniVa+LR), Strat. 3 (MRMR+AdaBo); CT All Radiomics: Strat. 1 (RFE+LR), Strat. 2 (UniVa+LR), Strat. 3 (MRMR+LR); CT Strain: Strat. 1 (Manual+LR), Strat. 2 (RFE+LR), Strat. 3 (UniVa+AdaBo) Strat. 4 (MRMR+SVM); Multi-Modality: Strat. 1 (RFE+LR), Strat. 2 (UniVa+LR), Strat. 3 (MRMR+LR)


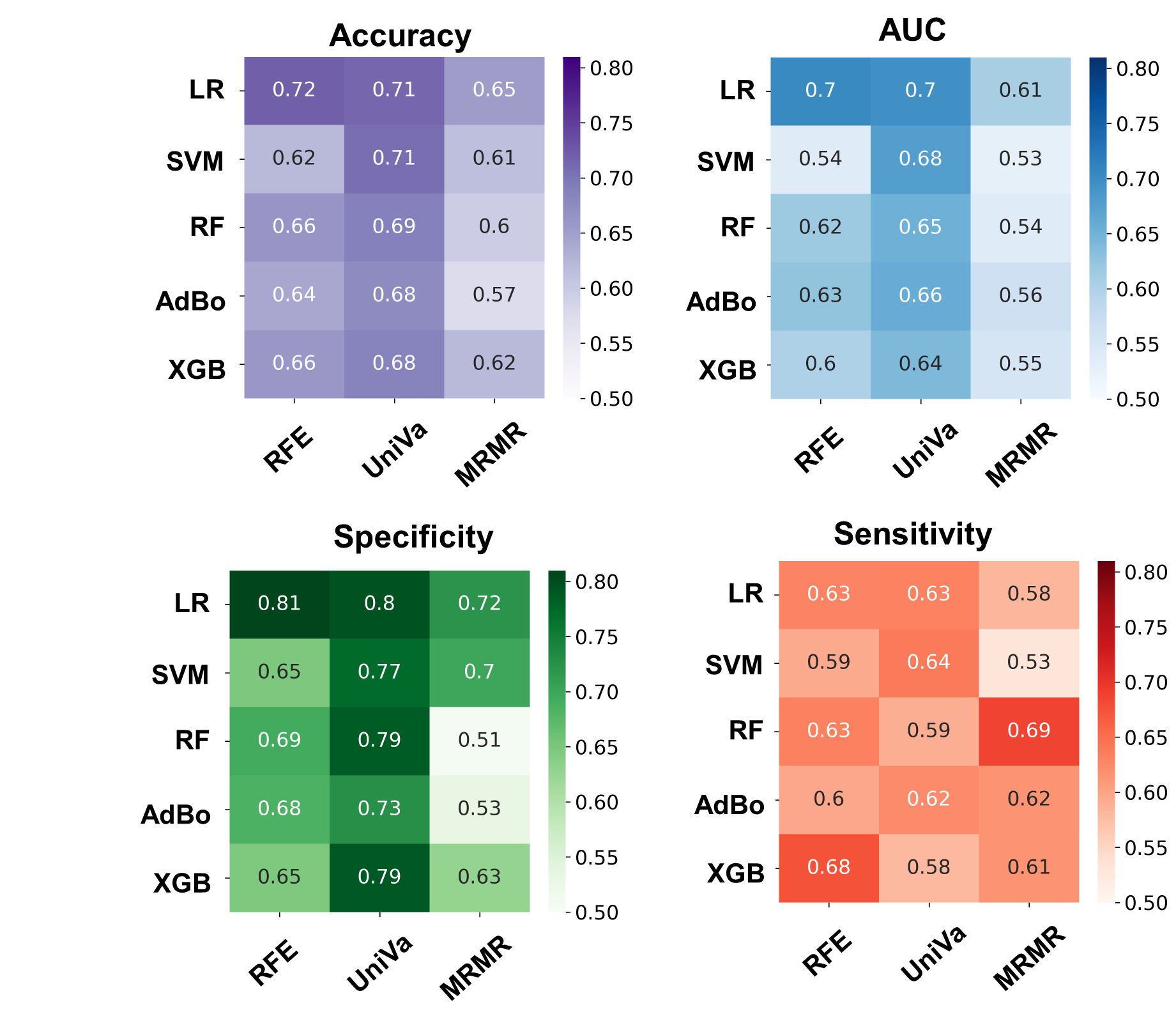


**Supplemental Figure 26:** Heat map displaying various metrics Clinical data. Classifiers include Logistic Regression (LR), Support Vector Machine (SVM), Random Forest (RF), AdaBoost (AdaBo), and eXtreme Gradient Boosting (XGB). Feature selection methods featured are Recursive Feature Elimination (RFE), Univariate Analysis (UniVa), and Minimum Redundancy Maximum Relevance (MRMR).


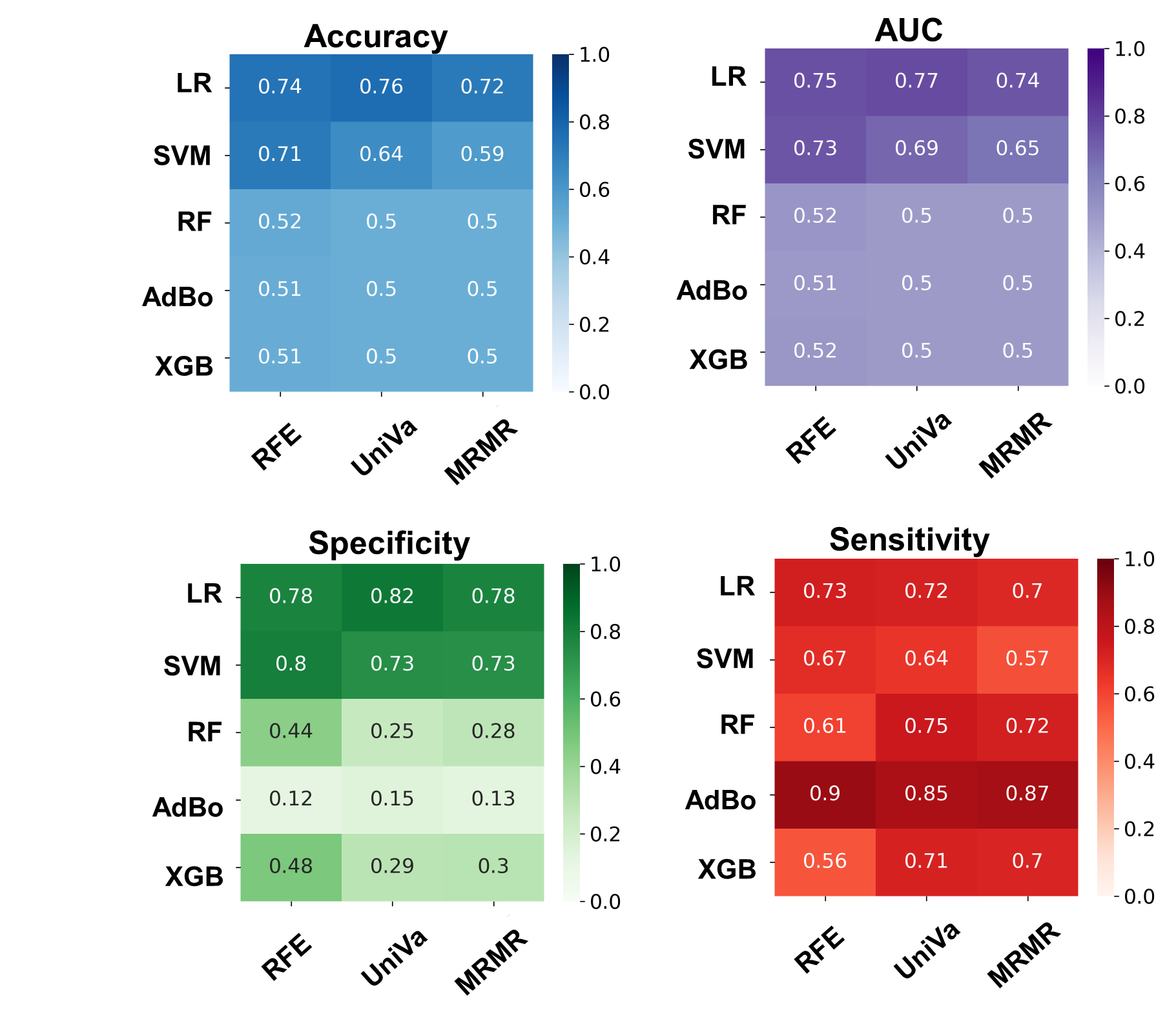


**Supplemental Figure 27:** Heat map displaying various metrics laboratory data. Classifiers include Logistic Regression (LR), Support Vector Machine (SVM), Random Forest (RF), AdaBoost (AdaBo), and eXtreme Gradient Boosting (XGB). Feature selection methods featured are Recursive Feature Elimination (RFE), Univariate Analysis (UniVa), and Minimum Redundancy Maximum Relevance (MRMR).


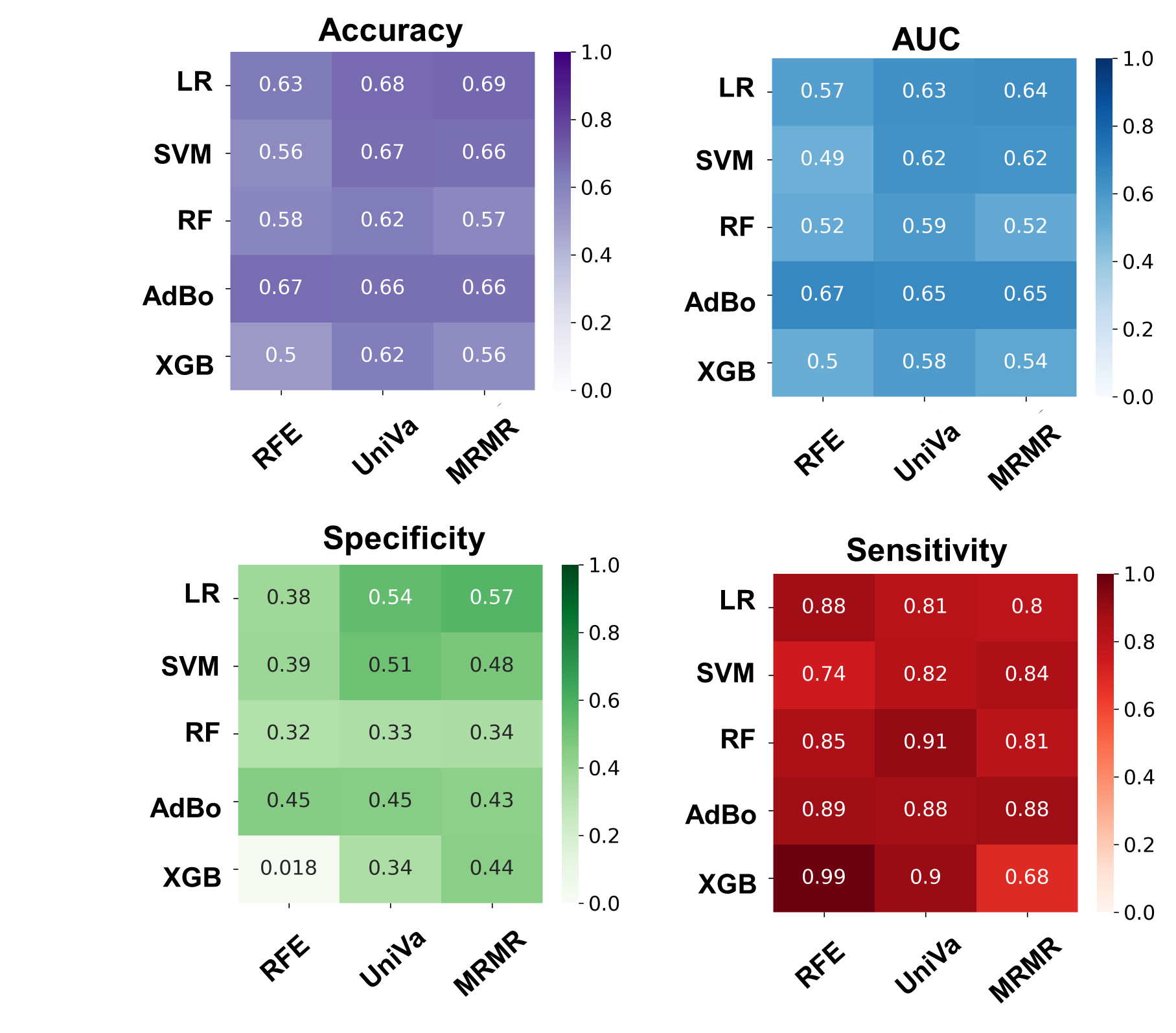


**Supplemental Figure 28:** Heat map displaying various metrics for ECG data. Classifiers include Logistic Regression (LR), Support Vector Machine (SVM), Random Forest (RF), AdaBoost (AdaBo), and eXtreme Gradient Boosting (XGB). Feature selection methods featured are Recursive Feature Elimination (RFE), Univariate Analysis (UniVa), and Minimum Redundancy Maximum Relevance (MRMR).


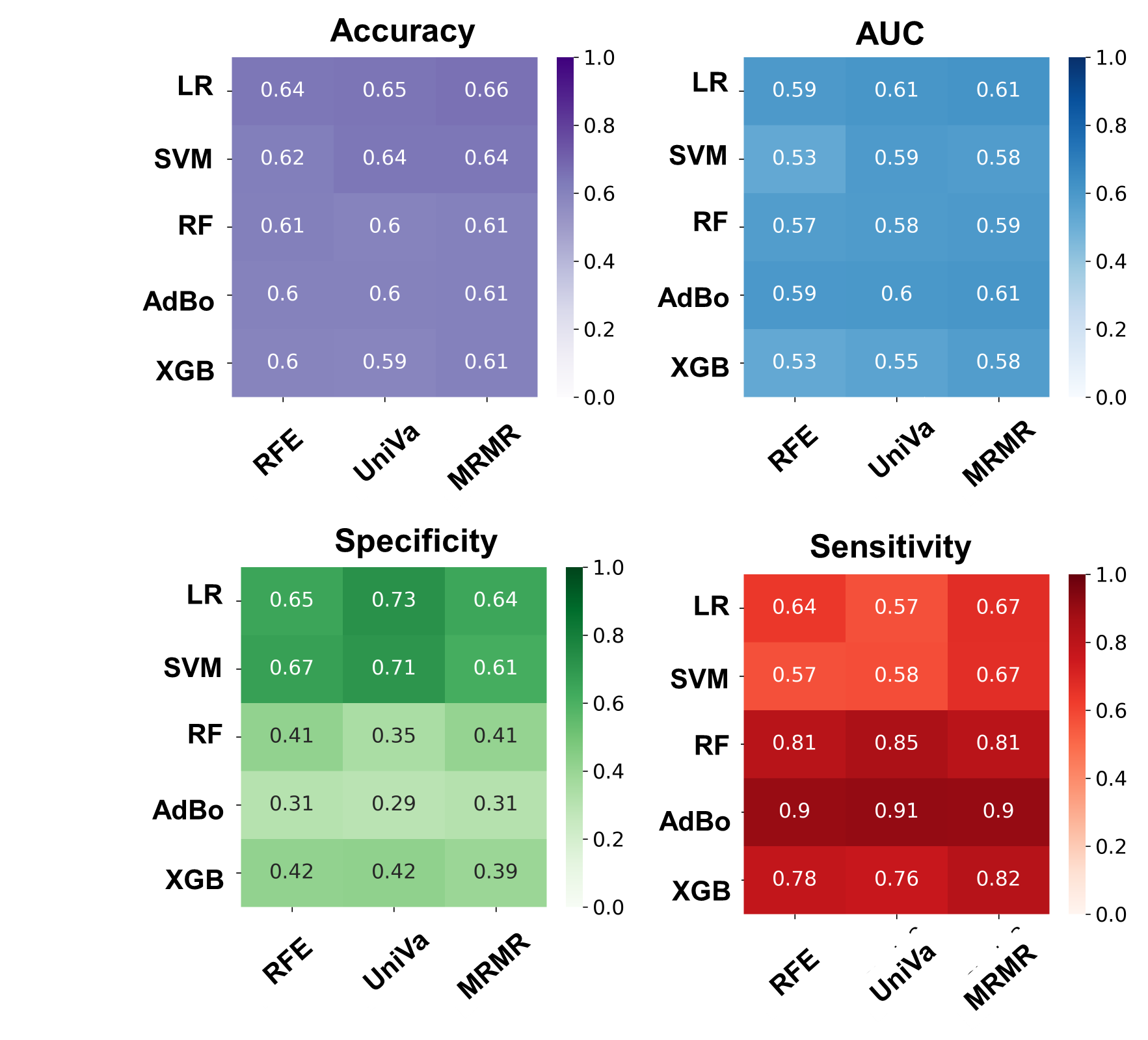


**Supplemental Figure 29:** Heat map displaying various metrics for invasive cath data. Classifiers include Logistic Regression (LR), Support Vector Machine (SVM), Random Forest (RF), AdaBoost (AdaBo), and eXtreme Gradient Boosting (XGB). Feature selection methods featured are Recursive Feature Elimination (RFE), Univariate Analysis (UniVa), and Minimum Redundancy Maximum Relevance (MRMR).


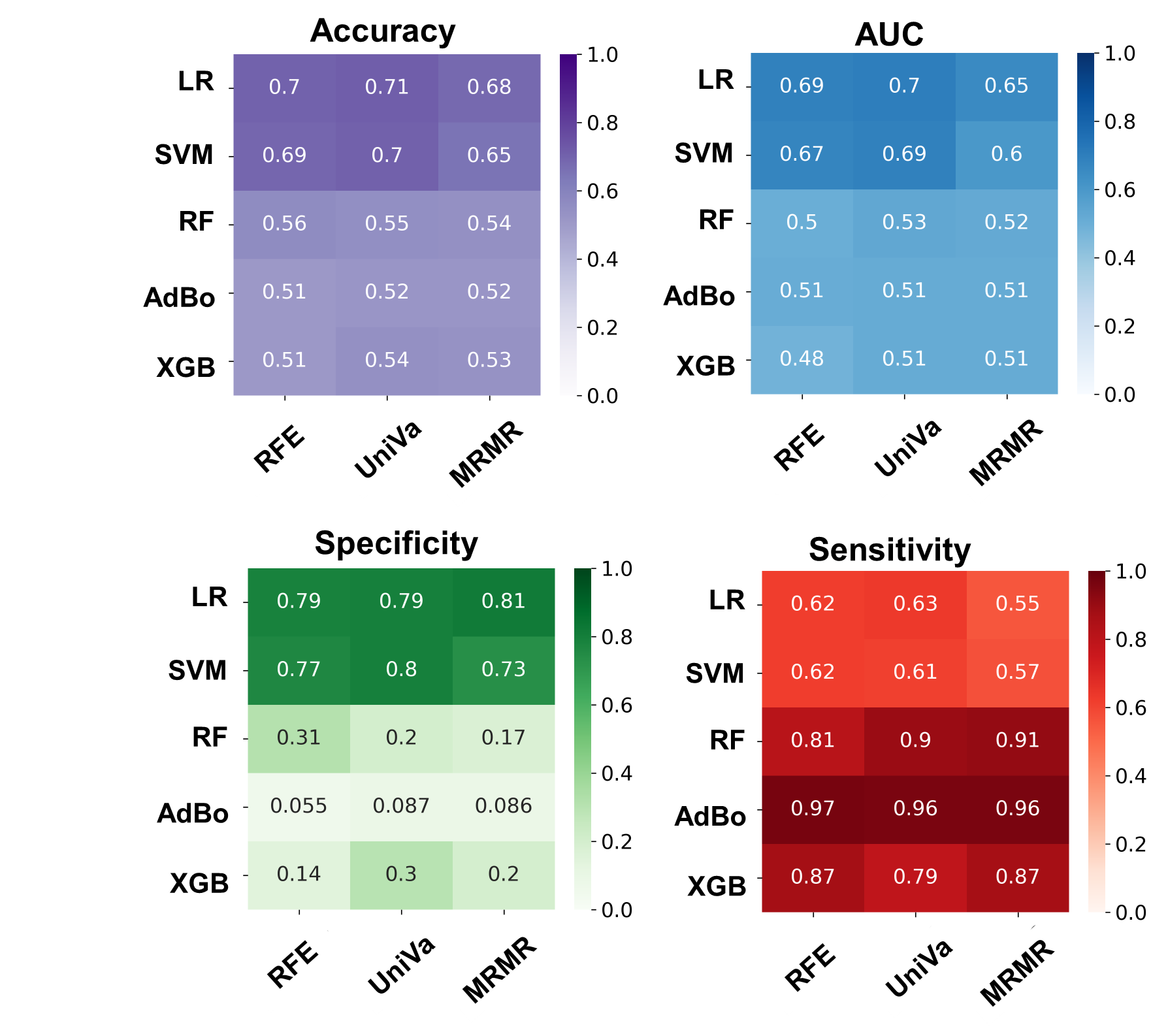


**Supplemental Figure 30:** Heat map displaying various metrics for interventional imaging data. Classifiers include Logistic Regression (LR), Support Vector Machine (SVM), Random Forest (RF), AdaBoost (AdaBo), and eXtreme Gradient Boosting (XGB). Feature selection methods featured are Recursive Feature Elimination (RFE), Univariate Analysis (UniVa), and Minimum Redundancy Maximum Relevance (MRMR).


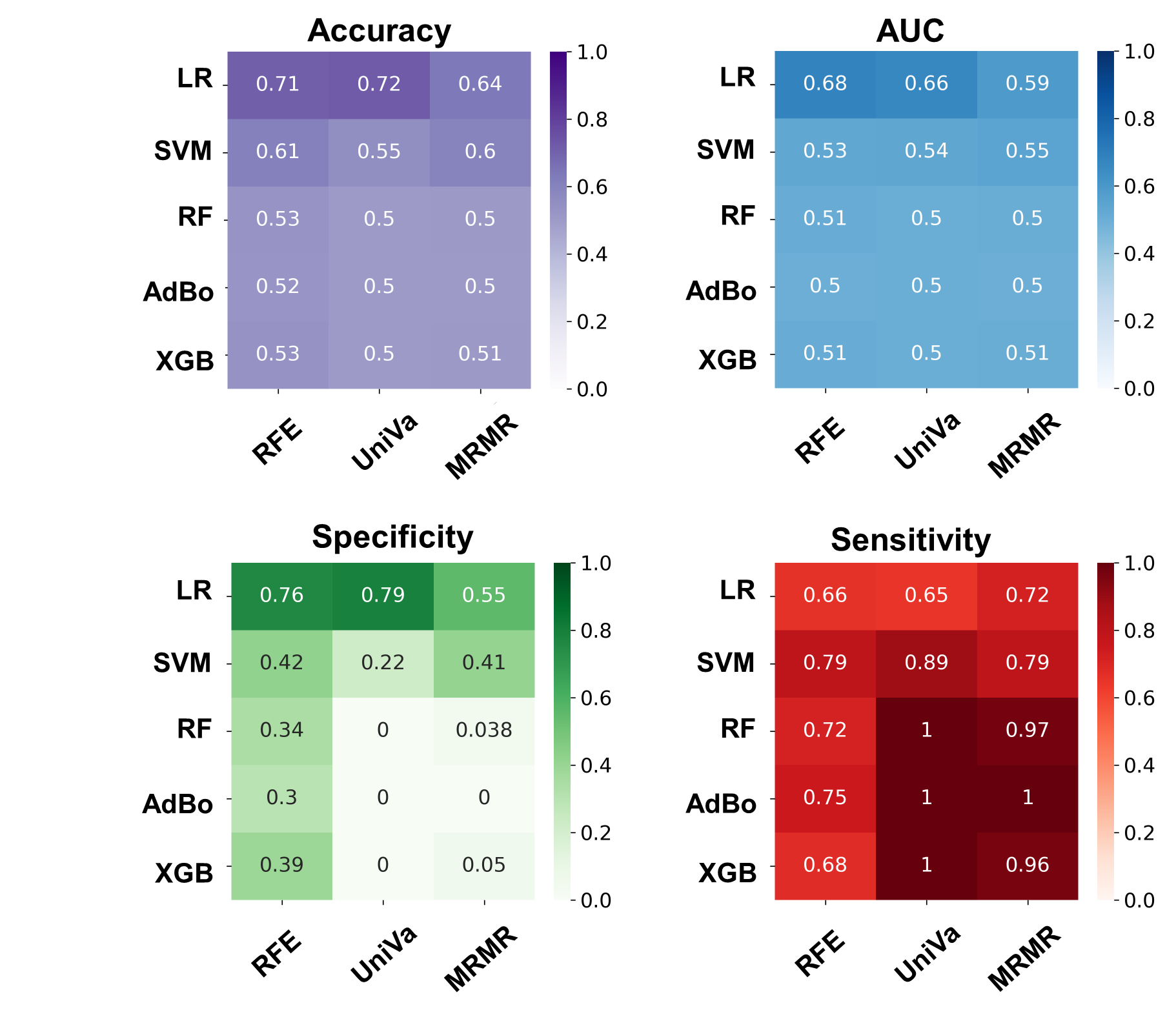


**Supplemental Figure 31:** Heat map displaying various metrics for CT non-contrast radiomics data. Classifiers include Logistic Regression (LR), Support Vector Machine (SVM), Random Forest (RF), AdaBoost (AdaBo), and eXtreme Gradient Boosting (XGB). Feature selection methods featured are Recursive Feature Elimination (RFE), Univariate Analysis (UniVa), and Minimum Redundancy Maximum Relevance (MRMR).


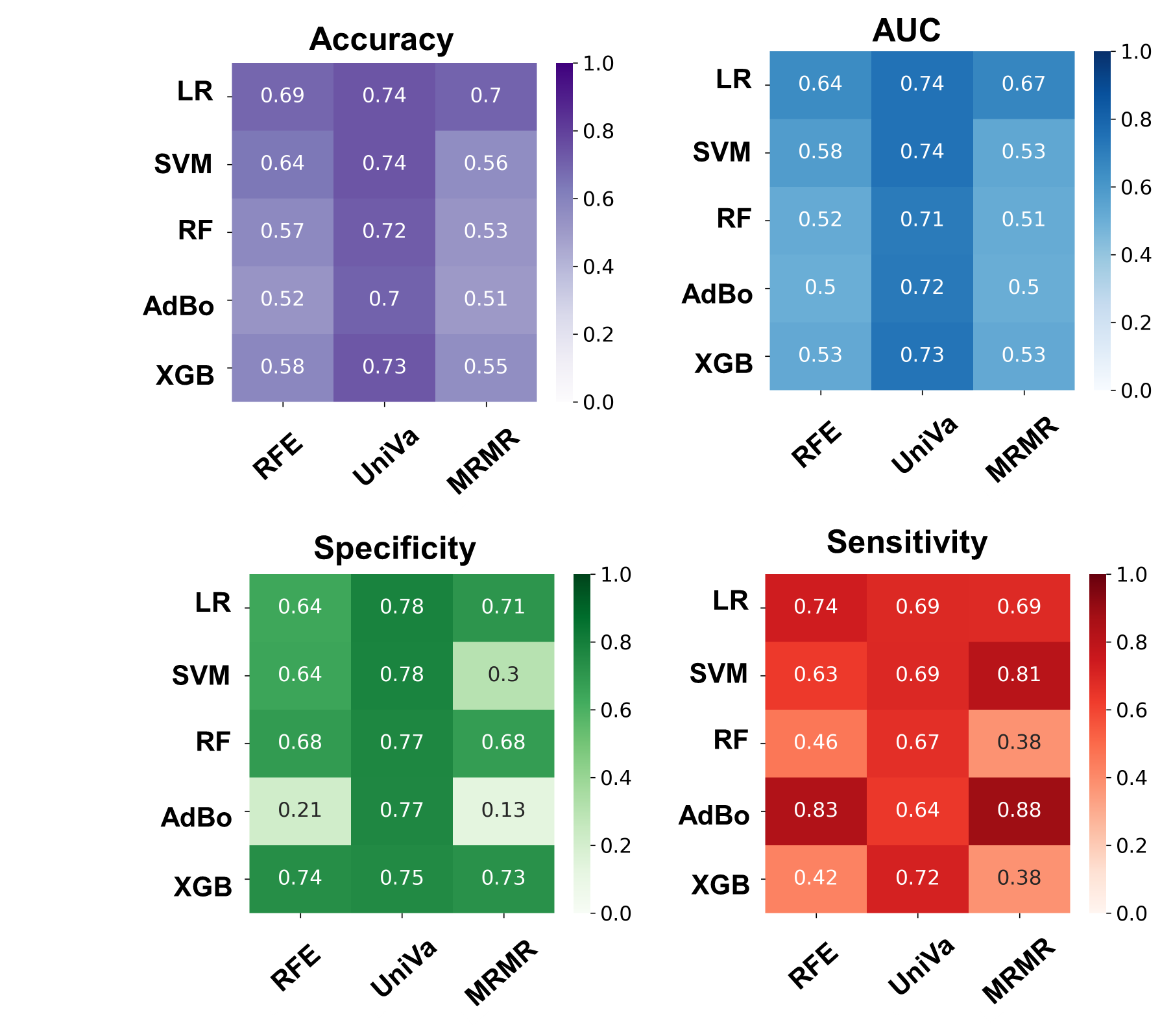


**Supplemental Figure 32:** Heat map displaying various metrics for CT diastolic radiomics data. Classifiers include Logistic Regression (LR), Support Vector Machine (SVM), Random Forest (RF), AdaBoost (AdaBo), and eXtreme Gradient Boosting (XGB). Feature selection methods featured are Recursive Feature Elimination (RFE), Univariate Analysis (UniVa), and Minimum Redundancy Maximum Relevance (MRMR).


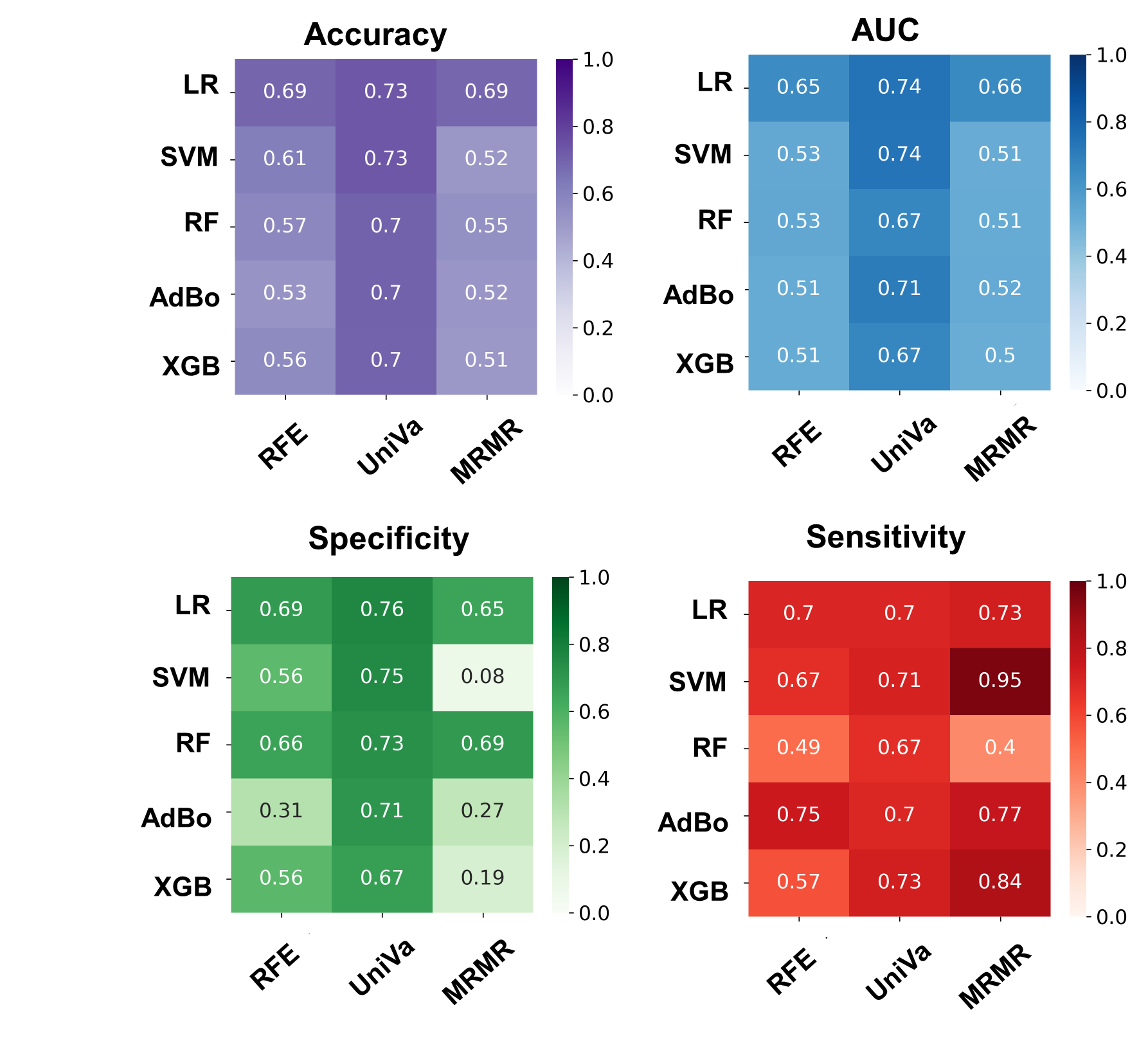


**Supplemental Figure 33:** Heat map displaying various metrics for CT systolic radiomics data. Classifiers include Logistic Regression (LR), Support Vector Machine (SVM), Random Forest (RF), AdaBoost (AdaBo), and eXtreme Gradient Boosting (XGB). Feature selection methods featured are Recursive Feature Elimination (RFE), Univariate Analysis (UniVa), and Minimum Redundancy Maximum Relevance (MRMR).


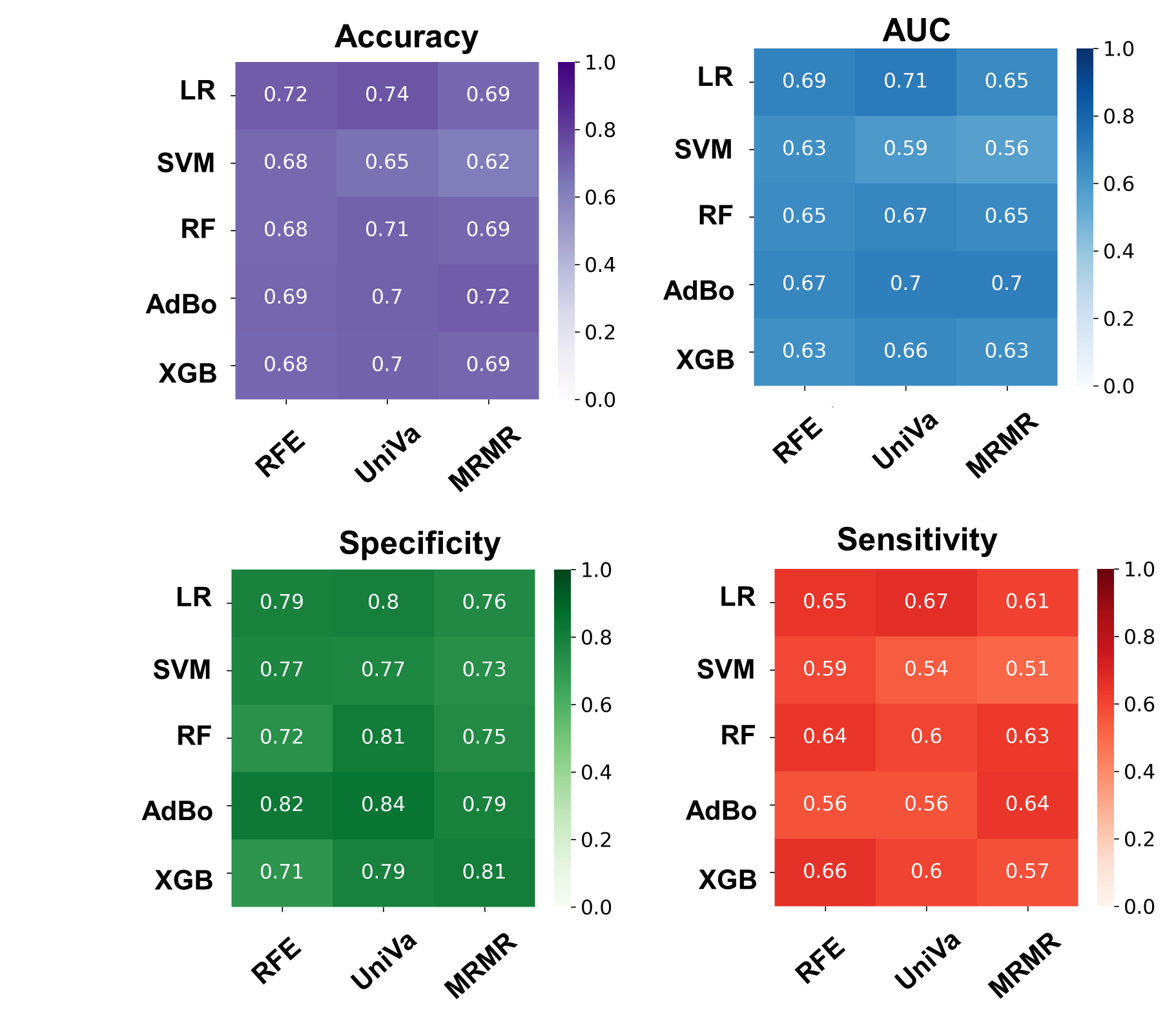


**Supplemental Figure 34:** Heat map displaying various metrics for CT delta Radiomics data. Classifiers include Logistic Regression (LR), Support Vector Machine (SVM), Random Forest (RF), AdaBoost (AdaBo), and eXtreme Gradient Boosting (XGB). Feature selection methods featured are Recursive Feature Elimination (RFE), Univariate Analysis (UniVa), and Minimum Redundancy Maximum Relevance (MRMR).


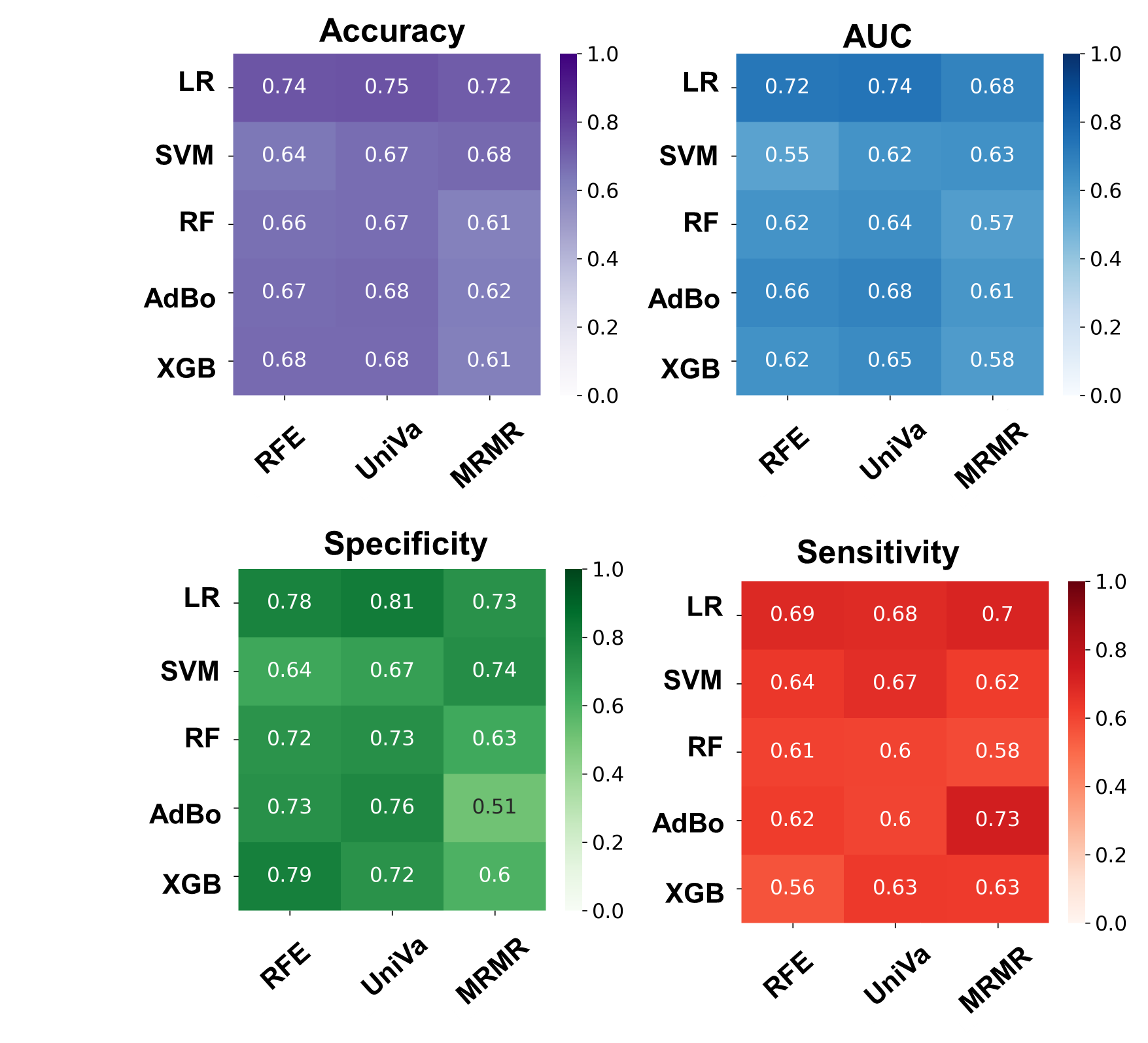


**Supplemental Figure 35:** Heat map displaying various metrics for CT all radiomics data. Classifiers include Logistic Regression (LR), Support Vector Machine (SVM), Random Forest (RF), AdaBoost (AdaBo), and eXtreme Gradient Boosting (XGB). Feature selection methods featured are Recursive Feature Elimination (RFE), Univariate Analysis (UniVa), and Minimum Redundancy Maximum Relevance (MRMR).


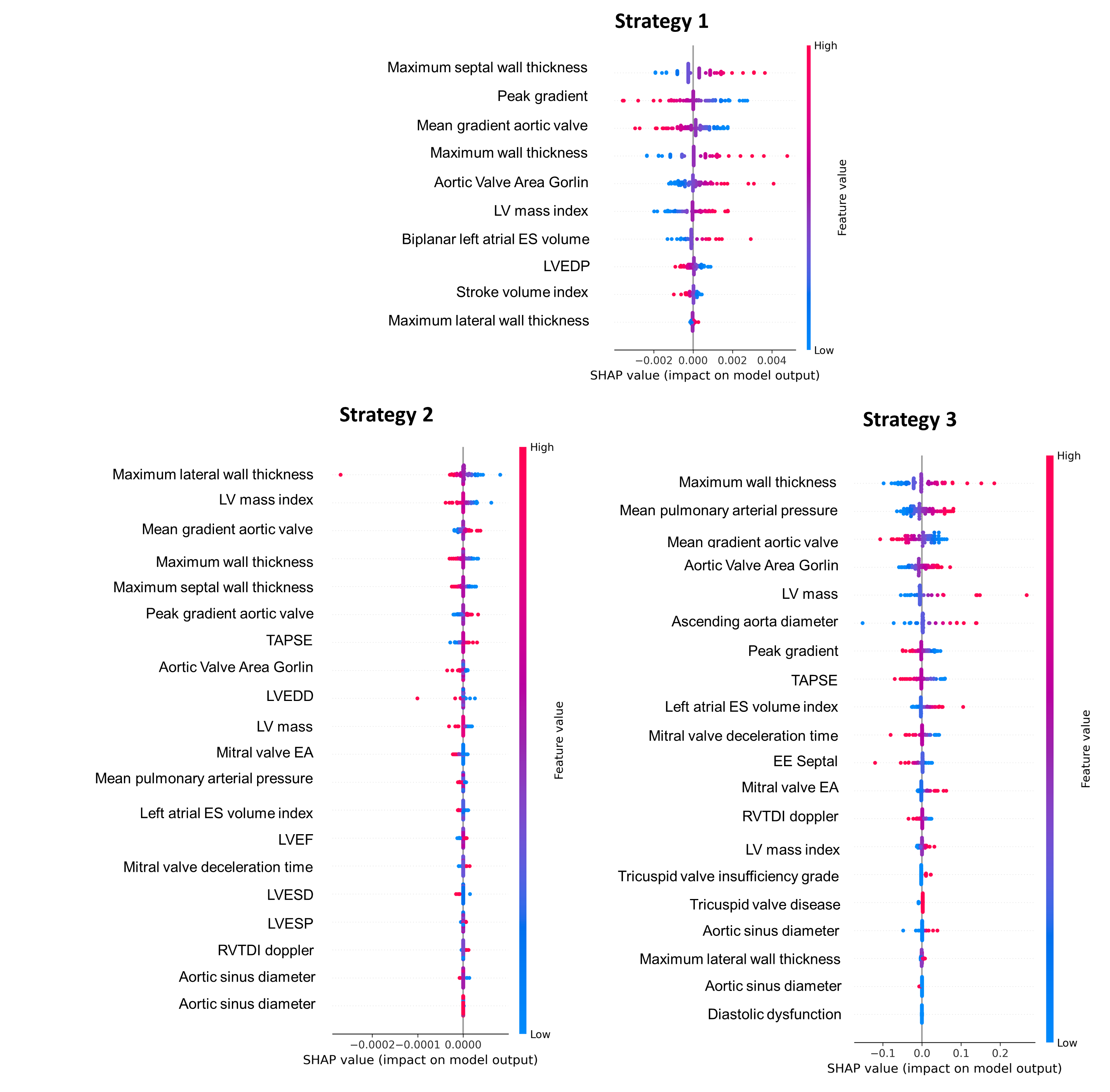


**Supplemental Figure 36:** SHAP (SHapley Additive exPlanations) summary plot displaying the impact of various features across top Echo models. This visualization highlights the contribution of individual features to the predictive performance of each model for ATTR-CM detection. Peak Gradient: Peak Gradientpeak pressure gradient of the aortic valve [mmHg], Mean Gradient: mean pressure gradient of the aortic valve [mmHg], Biplanar Left Atrial ES volume: Biplanar LAESVi [ml / BSA in m2], LV mass index: left ventricular mass index LVMi [g/m2], LVEDP: left ventricular end-diastolic pressure [mmHg], Maximum lateral wall thickness = maximum lateral wall thickness of the left ventricle [mm], Lvmassindexed: left ventricular mass index LVMi [g/m2], Maximum septal wallthickness: maximum septal wall thickness of the left ventricle [mm], TAPSE: Tricuspid Annular Plane Systolic Excursion [cm], Aortic valve area Gorlin = Estimated aortic valve area (AVA) [cm2] calculated by Gorlin equation, LVEDD: Left ventricular end-diastolic diameter [cm], Mitral valve EA: Ratio of the early to late diastolic filling (E/a) of the left ventricle, Left atrial end-systolic volume biplane indexed: Biplanar LAESVi [ml / BSA in m2], LVEFTTE: left ventricular ejection fraction [%], Mitral valve deceleration time: Deceleration Time of the mitral valve E-wave [ms], EE septal: Ratio of early diastolic filling (E-wave, pulswave doppler) and Mitral annular velocity (tissue wave doppler), RVTDI doppler: Tricuspid annular systolic velocity (Tissue Doppler). Aortic valve insufficiency grade: Grade of severity of aortic valve regurgitation,


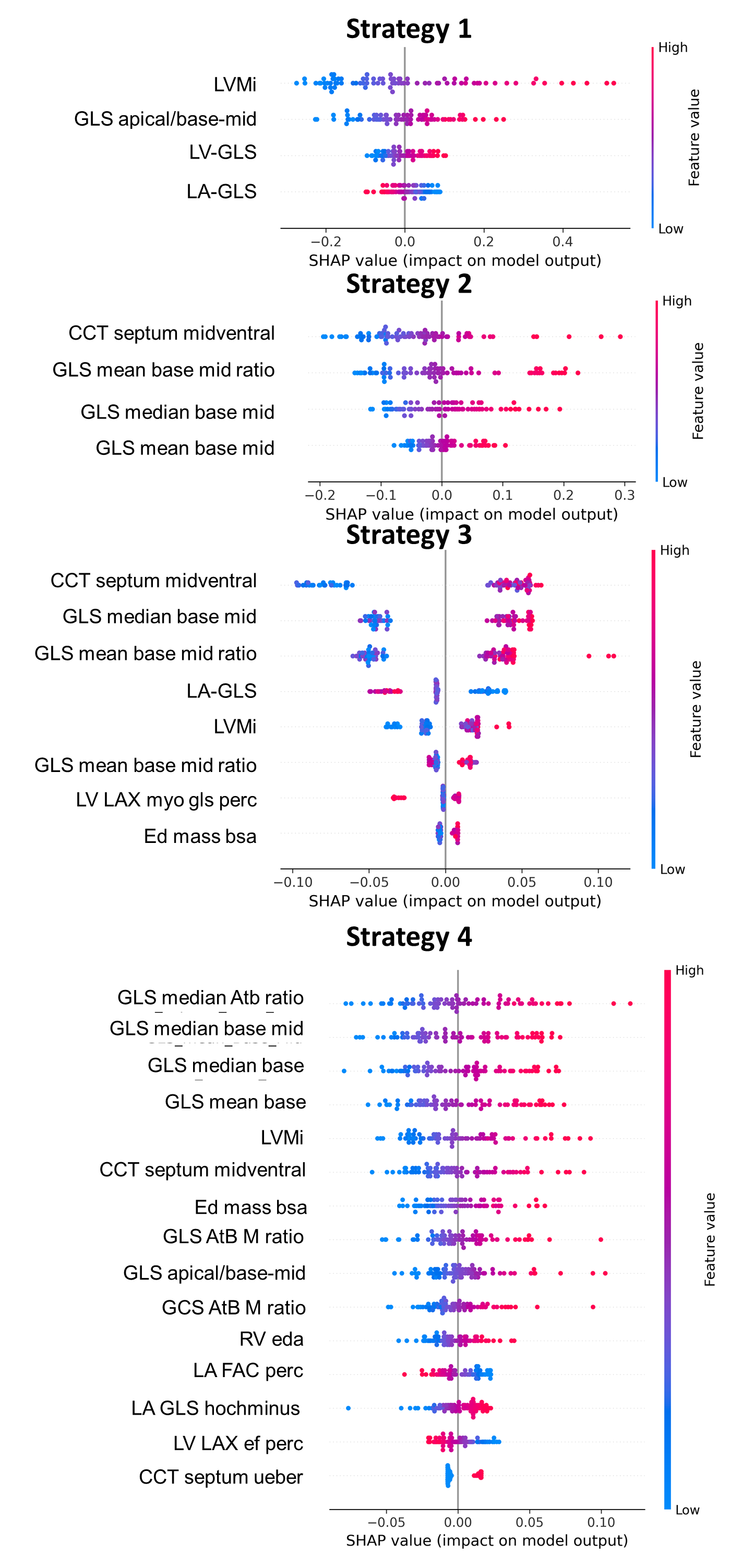


**Supplemental Figure 37:** SHAP (SHapley Additive exPlanations) summary plot displaying the impact of various features across top CT strain models. This visualization highlights the contribution of individual features to the predictive performance of each model for ATTR-CM deteciton. LVMi: Left ventricular mass (end-diastolic) index to gr/m2 , GLS apical/base-mid: Left ventricular global longitudinal strain apical /base and mid, LV-GLS: Left ventricular global longitudinal strain (%), LA-GLS: Left atrial Global longitudinal strain (%), CCT septum midventral: Midventricular septal wall thickness [mm] derived from cardiac CT, GLS mean base mid ratio: Ratio of mean left ventricular global longitudinal strain [%] bassal and mid, GLS median Base: Median left ventricular global longitudinal strain [%], Ed mass bsa: Left ventricular end-diastolic mass indexed to BSA [g/m2], LV LAX myo gls perc: Left ventricular global longitudinal strain in long axis [%], GLS median Atb ratio: left ventricular global longitudinal strain, GCS AtB M ratio: left ventricular global circumferential strain ratio Base/Mid, LA GLS hochminus: left atrial global longitudinal strain (rest needs to be clarified). LA FAC perc: Left atrial fractional area change [%], RV eda: Right ventricular end-diastolic area [cm2], CCT septum ueber= LV septal wall thickness >13mm, derived from cardiac CT, LV LAX ef perc=left ventricular ejection fraction long axis [%]

**
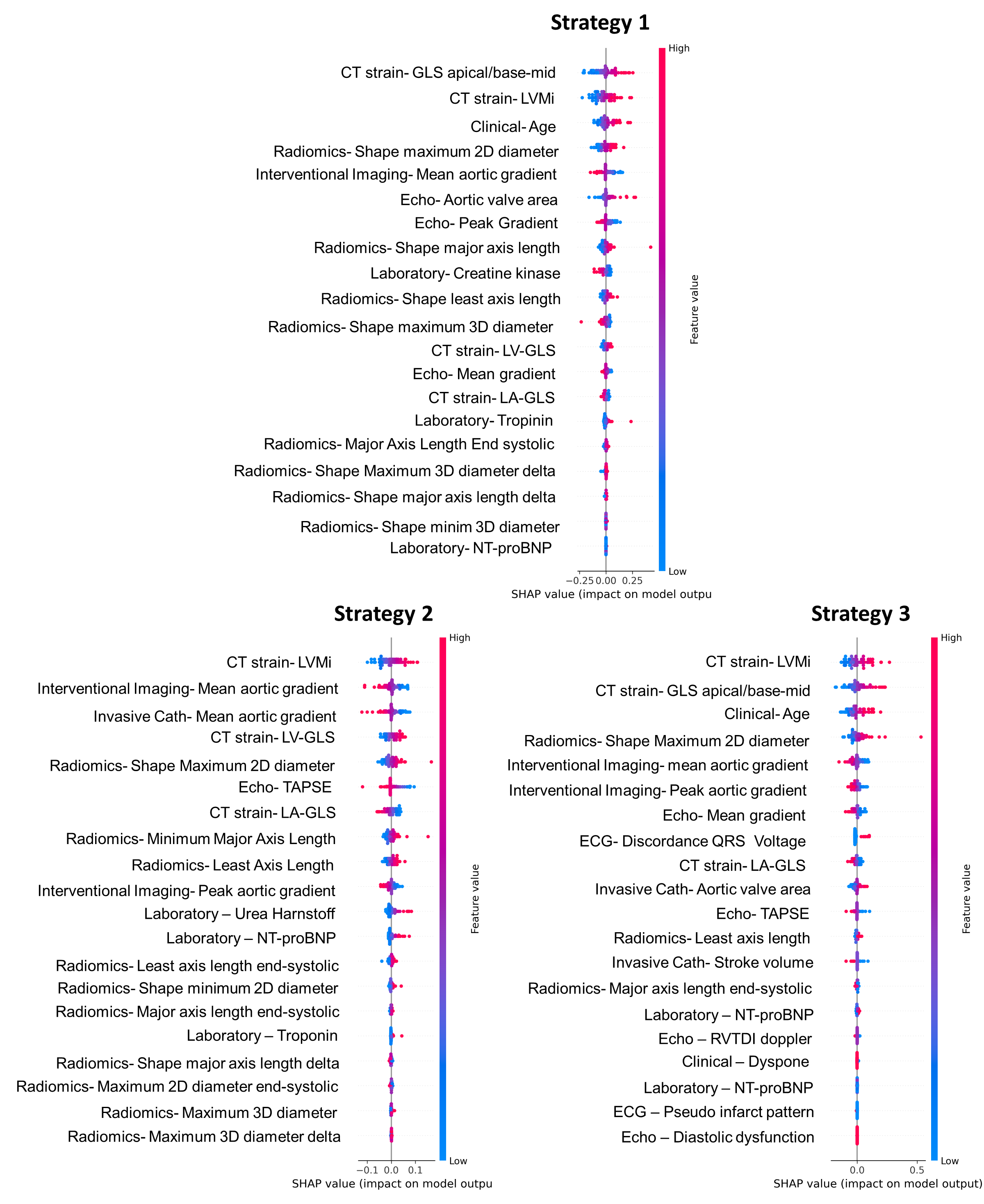
**

**Supplemental Figure 38:**  SHAP (SHapley Additive exPlanations) summary plot displaying the impact of various features across top miulit-modality models. This visualization highlights the contribution of individual features to the predictive performance of each model for ATTR-CM detection.


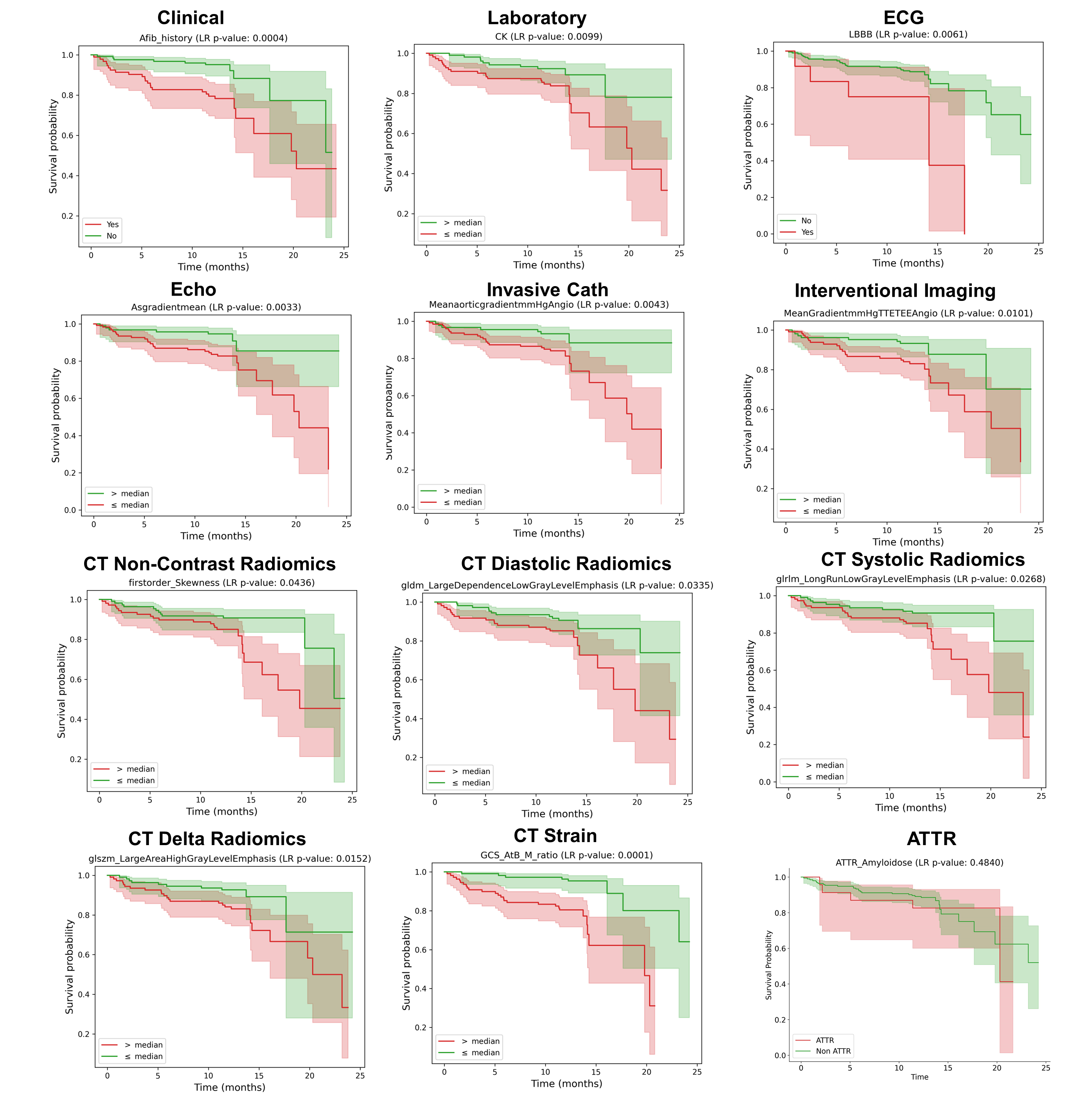


**Supplemental Figure 39:** Kaplan-Meier curves for all causes of mortality are stratified based on different features in each modality. The stratification categories are based on the median value of each feature, which illustrates survival probabilities over time for each group.


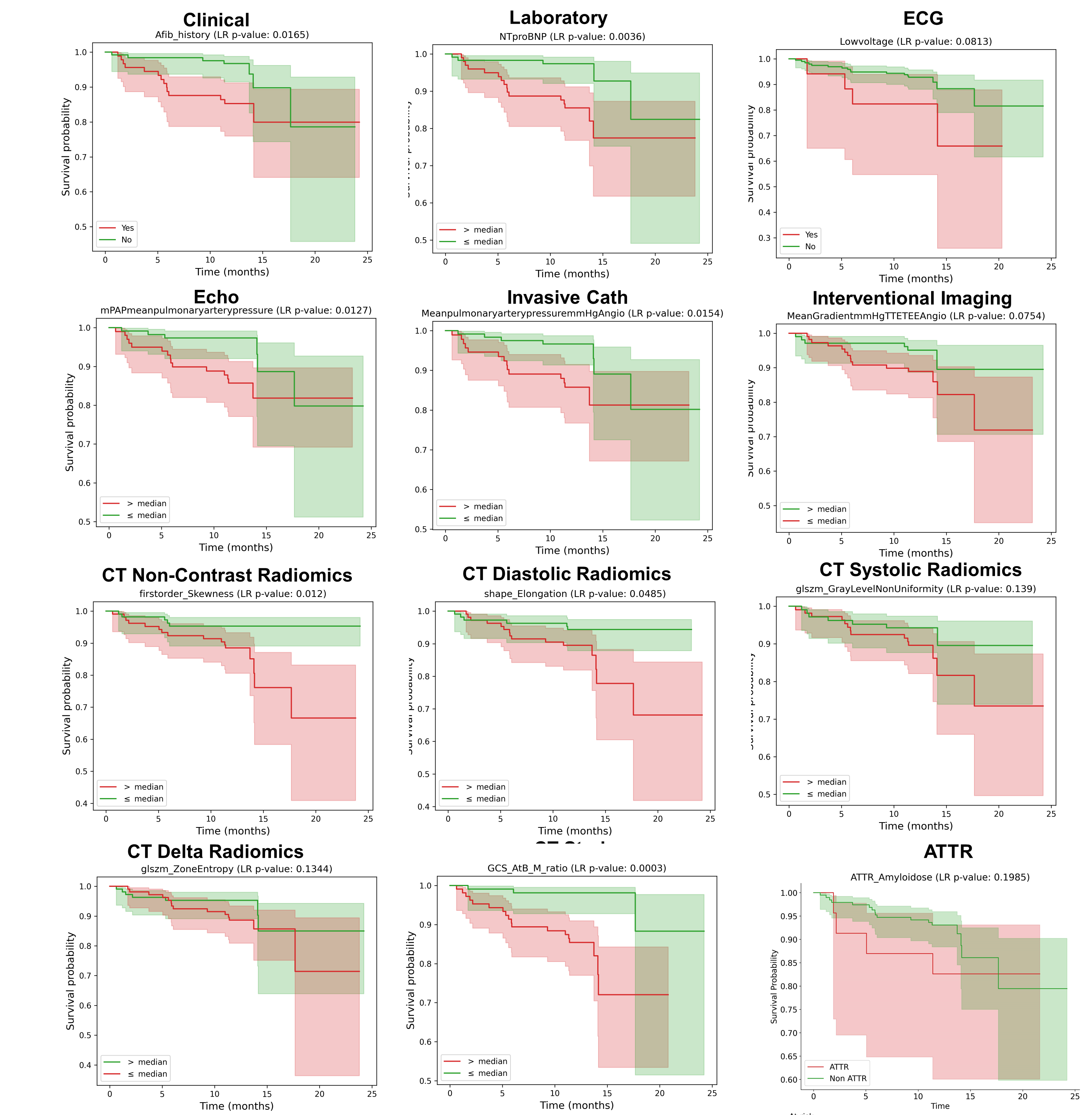


**Supplemental Figure 40:**  Kaplan-Meier curves for cardiovascular mortality are stratified based on different features in each modality. The stratification categories are based on the median value of each feature, which illustrates survival probabilities over time for each group.

**References**

1. Bernhard, B., et al., *Routine 4D Cardiac CT to Identify Concomitant Transthyretin Amyloid Cardiomyopathy in Older Adults with Severe Aortic Stenosis.* Radiology, 2023. **309**(3): p. e230425.
